# Gene Expression Alterations in the Cerebellum and Granule Neurons of *Cstb^−/−^* Mouse Are Associated with Early Synaptic Changes and Inflammation

**DOI:** 10.1371/journal.pone.0089321

**Published:** 2014-02-27

**Authors:** Tarja Joensuu, Saara Tegelberg, Eva Reinmaa, Mikael Segerstråle, Paula Hakala, Heidi Pehkonen, Esa R. Korpi, Jaana Tyynelä, Tomi Taira, Iiris Hovatta, Outi Kopra, Anna-Elina Lehesjoki

**Affiliations:** 1 Folkhälsan Institute of Genetics, Helsinki, Finland; 2 Department of Medical Genetics, Haartman Institute and Research Programs Unit, Molecular Neurology, University of Helsinki, Helsinki, Finland; 3 Neuroscience Center, University of Helsinki, Helsinki, Finland; 4 Department of Biosciences, Faculty of Biological and Environmental Sciences, University of Helsinki, Helsinki, Finland; 5 Department of Veterinary Biosciences, Faculty of Veterinary Medicine, University of Helsinki, Helsinki, Finland; 6 Institute of Biomedicine, Pharmacology, University of Helsinki, Helsinki, Finland; 7 Institute of Biomedicine, Biochemistry and Developmental Biology, University of Helsinki, Helsinki, Finland; 8 Mental Health and Substance Abuse Services, National Institute for Health and Welfare, Helsinki, Finland; University of Iowa Carver College of Medicine, United States of America

## Abstract

Progressive myoclonus epilepsy of Unverricht-Lundborg type (EPM1) is an autosomal recessively inherited neurodegenerative disease, manifesting with myoclonus, seizures and ataxia, caused by mutations in the cystatin B (*CSTB*) gene. With the aim of understanding the molecular basis of pathogenetic events in EPM1 we characterized gene expression changes in the cerebella of pre-symptomatic postnatal day 7 (P7) and symptomatic P30 cystatin B -deficient (*Cstb^−/−^*) mice, a model for the disease, and in cultured *Cstb^−/−^* cerebellar granule cells using a pathway-based approach. Differentially expressed genes in P7 cerebella were connected to synaptic function and plasticity, and in cultured cerebellar granule cells, to cell cycle, cytoskeleton, and intracellular transport. In particular, the gene expression data pinpointed alterations in GABAergic pathway. Electrophysiological recordings from *Cstb^−/−^* cerebellar Purkinje cells revealed a shift of the balance towards decreased inhibition, yet the amount of inhibitory interneurons was not declined in young animals. Instead, we found diminished number of GABAergic terminals and reduced ligand binding to GABA_A_ receptors in *Cstb^−/−^* cerebellum. These results suggest that alterations in GABAergic signaling could result in reduced inhibition in *Cstb^−/−^* cerebellum leading to the hyperexcitable phenotype of *Cstb^−/−^* mice. At P30, the microarray data revealed a marked upregulation of immune and defense response genes, compatible with the previously reported early glial activation that precedes neuronal degeneration. This further implies the role of early-onset neuroinflammation in the pathogenesis of EPM1.

## Introduction

Progressive myoclonus epilepsy of Unverricht-Lundborg type (EPM1, OMIM 254800) is an autosomal recessively inherited neurodegenerative disease characterized by stimulus-sensitive myoclonus, tonic-clonic seizures and ataxia with the disease onset at 6–16 years of age [1]. While epileptic seizures are usually well controlled with medication, myoclonus is resistant to treatment and severely incapacitating. EPM1 is caused by loss-of-function mutations in the cystatin B gene (*CSTB*), the most common of which is an expansion of a dodecamer repeat in the promoter region of the gene [2], [3]. A mouse model for the disease, the *Cstb*-deficient (*Cstb^−/−^*) mouse, presents with many of the clinical features of EPM1, especially myoclonus starting at the age of 1 month and progressive ataxia manifesting around 6 months of age [4]. One of the major neuropathological phenotypes in *Cstb^−/−^* mice is a severe loss of cerebellar granule neurons due to apoptotic death. Cerebella of *Cstb^−/−^* mice also show oxidative damage, reflected by depletion of antioxidants and increased lipid peroxidation [5]. Moreover, we recently reported striking, early microglial activation in *Cstb^−/−^* brain, which precedes the emergence of myoclonus and is followed by widespread astroglial activation and selective neuronal loss [6].

CSTB is a cysteine protease inhibitor that controls the activity of lysosomal cysteine cathepsins. Cathepsin activity has been found to be increased in EPM1 patient lymphoblastoid cells [7]. In *Cstb^−/−^* mice, cathepsin B has been shown to mediate the increased sensitivity to oxidative stress -induced cell death [5] and cathepsin removal or inhibition by other means to ameliorate the neurodegenerative phenotype of *Cstb^−/−^* mice [8], [9]. Yet, the molecular mechanisms leading to EPM1 are still largely unknown and it is possible that CSTB has functions independent of cathepsins.

The changes in gene expression patterns may reveal dysregulated pathways and functional cascades causative for pathological processes. A previous array-based approach to study differentially expressed genes in aged, fully symptomatic 8-month-old *Cstb^−/−^* mice revealed changes in genes related to glial activation and immunological pathways, reflecting the advanced pathological state of *Cstb^−/−^* brain at that age [10]. However, we recently reported pathological changes in *Cstb^−/−^* mice that begin with intense microglial activation already at 2 weeks of age, prior to the appearance of the first clinical symptom, myoclonus [6]. To further understand the molecular processes involved in EPM1, we used cerebellum as a model system and generated microarray-based gene expression patterns from immature pre-symptomatic and young symptomatic cerebella and cultured cerebellar granule cells of *Cstb^−/−^* mice. Gene Ontology (GO) analysis of expression changes in presymptomatic mice highlighted disruption in synaptogenesis and in synaptic function and maintenance, and in symptomatic mice, in immune and defense response genes. Consequences in synaptic function were characterized using electrophysiological and ligand-binding analyses along with immunohistological studies in neurons and cerebellar tissue of *Cstb^−/−^* and control mice. Conclusively, these experiments suggest that alterations in GABAergic signaling accompanied with early-onset neuroinflammation in *Cstb^−/−^* mice are essential contributors in the pathogenesis of EPM1.

## Methods

### Ethics statement

The Animal Ethics Committee of the State Provincial Office of Southern Finland approved all animal research protocols (decisions ESLH-2005-00507/Ym-23, ESLH-2007-05788/Ym-23, ESAVI-2010-07744/Ym-23).

### Animals

*Cstb^−/−^* mice were obtained from The Jackson Laboratory (Bar Harbor, ME; 129-*Cstb^tm1Rm^*/SvJ; stock #003486) [4]. Wild type littermates were used as controls.

### RNA isolation and microarray hybridization

RNA isolation for microarray hybridization and real-time quantitative PCR (qPCR) were done from cerebella of P7 (n = 5 per genotype for hybridization, n = 10 per genotype for qPCR, males and females) and P30 (n = 3 per genotype for hybridization, all males) *Cstb^−/−^* and control mice, as well as from cultured cerebellar granule neurons dissected from P5 *Cstb^−/−^* and control mice (n = 5 per genotype for hybridization, males and females). Mice were anesthetized with CO_2_ and sacrificed by decapitation. The dissected cerebella were homogenized using Lysing Matrix D tubes (Qbiogene, Carlsbad, CA, USA) and FastPrep® FP120 Instrument (Qbiogene, Carlsbad, CA, USA) with Proteinase K digestion (20 mg/ml, >30 U/mg, Finnzymes, Espoo, Finland) according to the manufacturer's instructions. Cerebellar granule cell cultures were prepared as described [11] and cultured for two days *in vitro* (2 DIV). Proliferation of glial cells was prevented by addition of 10 µM cytosine 1-β-d-arabinofuranoside (Sigma, St. Louis, MO, USA) 16–20 h after plating. Cultures were controlled for neuron health and morphology as well as for glial content. Total RNA was extracted using the ABI Prism 6100 Nucleic Acid PrepStation (Applied Biosystems, Foster City, CA, USA) from cerebella and PerfectPure RNA Cultured Cell kit (5 PRIME GmbH, Hamburg, Germany) from cerebellar granule cells according to the manufacturer's instructions. RNA purity was confirmed by spectrophotometer and integrity by Agilent 2100 Bioanalyzer (Agilent Technologies, Palo Alto, CA, USA). The subsequent sample preparation and hybridization of each cRNAs on GeneChip® Mouse Genome 430 2.0 arrays (Affymetrix, Santa Clara, CA, USA) were performed in Helsinki Biomedicum Biochip Center (Finland).

### Microarray data analysis

The quality control of each microarray was carried out by the Affymetrix GeneChip® Operating Software (GCOS) v1.4 (Affymetrix Inc., Santa Clara, CA, USA), and the raw data were imported to GeneSpring GX software (Silicon Genetics, Incorporated, Redwood City, CA, USA). Expression signal values were generated using the RMA (Robust Multiarray Average) algorithm [12] for background adjustment, quantile-normalization and summarization. For the normalization, the median expression level of a given gene across all samples (per gene normalization) was used. The data were interpreted from groups of replicate samples from the same experimental condition, and viewed in the log-of-ratio mode. Principal component analysis was performed as a second quality control at the sample level to evaluate grouping of the samples within the same genotype close to each other. All samples, except two from P7 mice (one from both genotypes) and one from P5 + 2 DIV *Cstb^−/−^* cerebellar granule cells, were used for the final data analysis. Filtering of the probe sets was based on the expression with lower and upper cutoff 20 and 100 percentile and on the fold-change (FC) with cutoff 1.3. In overall statistics of differentially expressed genes, t-test unpaired equal variance with corrected p<0.05 (cerebella) and p<0.01 (cerebellar granule cells) with Benjamini-Hochberg multiple testing correction was used. Hierarchical clustering analysis based on gender and genotype was performed for the differentially expressed genes of P7 *Cstb^−/−^* mice and P5 + 2 DIV *Cstb^−/−^* cerebellar granule cells. The genotype was the most descriptive, and the samples did not cluster according to gender (data not shown).

Web-based tool WebGestalt [13] (http://bioinfo.vanderbilt.edu/webgestalt/) and Ingenuity Systems Pathway Analysis (IPA) (Ingenuity System Incorporated, Redwood City, CA, USA; http://www.ingenuity.com/) were used in parallel to interpret the differentially expressed genes in the context of biological, molecular and cellular functions. Only gene ontology (GO) categories with at least two terms were considered and the significance of enrichment of genes in each category (p-value) was calculated using hypergeometric test adjusted for multiple testing using the Benjamini-Hochberg method. The microarray data are available in the Gene Expression Omnibus (GEO) database http://www.ncbi.nlm.nih.gov/geo/query/acc.cgi?acc=GSE47516.

### Real-time quantitative PCR

DNase (TURBO™; Ambion®, Austin, TX, USA) treated total RNA was reverse transcribed to cDNA (iScript cDNA Synthesis Kit; BioRad Laboratories, Berkeley, CA, USA) and quantified by qPCR on the ABI Prism 7000 Sequence Detection System (Applied Biosystems, Foster City, CA, USA) using Taqman® Gene Expression Assays (GABA_A_ receptor δ (*Gabrd)*, Mm01227754_m1, and GABA_A_ receptor *α6 (Gabra6)*, Mm01266203_g1) with Taqman® PCR master mix (Applied Biosystems, Foster City, CA, USA). TATA-box binding protein (*Tbp*, Mm00446973_m1) was used as an endogenous control. All reactions were prepared in duplicate and three separate runs were prepared for each sample. The mean expression level of samples was compared to mean expression level of control mice and calculated as FC of the controls (± SE). The data were calculated by standard-curve method with DataAssist software Version 3.01 (Applied Biosystems, Foster City, CA, USA) and Student's t-test with p<0.05 considered as statistically significant.

### Western blot analyses

Cerebella of P7 and P30 *Cstb^−/−^* and control mice (n = 3 per genotype) were lysed with 50 mM Tris (pH 8.0), 0.5% Nonidet P-40, 10% glycerol, 0.1 mM EDTA, 250 mM NaCl, 0.1 mM Na_3_VO_4_, 50 mM NaF, 4 mM dithiothreitol (DTT), 1× Protein inhibitor cocktail (Roche, Basel, Switzerland) using Lysing Matrix D tubes (Qbiogene, Carlsbad, CA, USA) and FastPrep® FP120 Instrument (Qbiogene, Carlsbad, CA, USA). Lysed proteins (15 µg) were separated with Protean TGX precast gels (Bio-Rad Laboratories, Hercules, CA, USA) and transferred on the nitrocellulose membrane. The primary antibodies used were rabbit anti-rat GABRA6 (1∶1000) (Synaptic Systems, Göttingen, Germany) and mouse anti-rat β-tubulin (1∶10 000) (Sigma, St. Louis, MO, USA), and the secondary antibodies used were anti-rabbit-IRDye 800CW (1∶10 000) (LI-COR Biosciences, Lincoln, NE, USA) and anti-mouse-Alexa Fluor 680 (1∶10 000) (Invitrogen®, Life Technologies, Carlsbad, CA, USA). The bands were detected with Odyssey infrared reader (LI-COR Biosciences, Lincoln, NE, USA). Signal intensities were detected with Image Studio 3.1 (LI-COR Biosciences, Lincoln, NE, USA) and normalized to the intensity of β-tubulin.

### Electrophysiology

Brains from P7 *Cstb^−/−^* and control mice (n = 7-10 per genotype) were dissected in ice-cold 124 mM NaCl, 3 mM KCl, 1.25 mM NaH_2_PO_4_, 10 mM MgSO_4_, 26 mM NaHCO_3_, 10–15 mM D-glucose, 1 mM CaCl_2_, saturated with 5% CO_2_/95% O_2_. Cerebellar slices (350 µm) were cut horizontally with a vibratome (Vibratome Co., St. Louis, MO, USA) in the above solution and stored at room temperature in 124 mM NaCl, 3 mM KCl, 1.25 mM NaH_2_PO_4_, 4 mM MgSO_4_, 26 mM NaHCO_3_, 10–15 mM D-glucose, 1 mM CaCl_2_, saturated with 5% CO_2_/95% O_2_. The slices were used 1–4 h after cutting.

For electrophysiological recordings the slices were placed in a submerged chamber and superfused with artificial cerebrospinal fluid (ACSF): 124 mM NaCl, 3 mM KCl, 1.25 mM NaH_2_PO_4_, 1 mM MgSO_4_, 26 mM NaHCO_3_, 15 mM D-glucose, 2 mM CaCl_2_, saturated with 5% CO_2_/95% O_2_, at a rate of 2–3 ml/min (32°C). Whole-cell recordings were obtained from Purkinje cells using the Multiclamp 700B amplifier (Molecular Devices, Sunnyvale, CA, USA). Cells were voltage-clamped at 0 mV with 4–5 MΩ pipettes filled with 135 mM CsMeSO_4_, 10 mM Hepes, 0.5 mM EGTA, 4 mM Mg-ATP, 0.3 mM Na-GTP and 2 mM NaCl (285 mOsm), pH 7.2. At 0 mV GABAergic currents were seen as outward and glutamatergic currents as inward. Recordings where access resistance was higher than 25 MΩ were discarded.

Axoscope 10.2 (Molecular Devices, Sunnyvale, CA, USA) was used for data acquisition. Offline analysis was done using MiniAnalysis 6.0.7 program (Synaptosoft, GA, USA). Spontaneous events were detected using peak detector algorithm, and all events were confirmed visually. The chi-square test and two-tailed Student's t-test were used for statistical analysis (GraphPad Prism 5.0c; GraphPad Software, La Jolla, CA, USA) with p<0.05 considered as statistically significant.

### Immunohistochemistry

Under terminal anesthesia induced with pentobarbital (80–150 mg/kg, Mebunat Vet, Orion, Finland) the mice (P7, P14, P20 and P30, n = 4–5 per genotype) were immersiofixed (P7) or intracardially perfusion fixed (P14, P20, P30) with 4% paraformaldehyde. Brains were dissected and postfixed with 4% paraformaldehyde for one week. Paraffin sections (8 µm) were dewaxed with xylene and descending series of alcohol. Antigen retrieval was performed by lightly boiling sections in 10 mM sodium citrate, 0.05% Tween-20, pH 6.0 for 20 min, followed by cooling at RT for 30 min. Sections were blocked with 5% FCS, 0.1% Triton X-100 in PBS and primary antibody diluted to 1% FCS in PBS was incubated overnight.

Cultured cerebellar granule cells growing on coverslips were fixed with 4% paraformaldehyde for 15 min and permeabilized with 0.1% Triton X-100 in PBS for 10 min. Coverslips were blocked with 10% FCS in PBS and primary antibody diluted to 1% FCS in PBS was incubated overnight.

Secondary antibodies were diluted to 1% FCS in PBS and incubated for 30 min. Nuclei were counter stained with 1 ng/ml Hoechst 33258 (Molecular Probes®, Life Technologies, Carlsbad, CA, USA).

For stereological analysis of interneurons, mice were euthanized at P14 (n = 3 per genotype) and P30 (n = 6 per genotype). Brains were removed, bisected along the midline and immersion fixed in 4% paraformaldehyde for one week before cryoprotection in 30% sucrose in TBS containing 0.05% sodium azide. Frozen sagittal sections of 40 µm were cut through the brains and collected as series in cryoprotectant solution (30% ethylene glycol, 15% sucrose, 0.05% sodium azide in TBS). One-in-six series of free-floating sections were immunostained with anti-parvalbumin (P14) or Nissl stained (P30) as described previously [14].

The specificity of all immunostainings was verified by controls in which the primary antibody was omitted.

### Antibodies for immunohistochemistry

Mouse anti-carp parvalbumin (1∶5000) (Swant, Marly, Switzerland); rabbit-anti-GABA (1∶250) (Sigma, St. Louis, MO, USA); rabbit anti-human synapsin 1 (1∶200) (Cell Signaling Technology, Danvers, MA, USA); mouse anti-rat vesicular GABA transporter (VGAT, 1∶500), rabbit anti-rat gephyrin (1∶500), rabbit anti-rat GABRA6 (1∶500), rabbit anti-rat vesicular glutamate transporters 1 (VGLUT1, 1∶250) and 2 (VGLUT2, 1∶250) (Synaptic Systems, Göttingen, Germany).

Secondary antibodies were as follows: anti-mouse-Alexa Fluor 564, anti-rabbit-Alexa Fluor 564, anti-mouse-Alexa Fluor 488, anti-rabbit-Alexa Fluor 488 (1∶200) (Invitrogen®, Life Technologies, Carlsbad, CA, USA).

### Counts of interneuron number

StereoInvestigator software (Microbrightfield, Inc, Williston, VT, USA) was used to obtain unbiased optical fractionator estimates of interneuron number in molecular layer of cerebellum from parvalbumin (P14) and Nissl (P30) stained 40 µm sections. Parvalbumin was used for P14 as at this age molecular layer still contains migrating granule cells that are difficult to reliably differentiate from interneurons in Nissl stain. All neuronal cell bodies on molecular layer of P30 mice were considered to be interneurons in Nissl stained sections. Measurements were performed as described previously [15]. The cut section thickness was nominally 40 µm, but after mounting and dehydration the actual thickness of sections was 15 µm. The optical fractionator was run using a guard height of 0.5 µm at both the top and bottom of the section, meaning that the actual depth of the fractionator was 14 µm. The following sampling scheme was used: grid area 30 625 µm^2^, frame area 2 400 µm^2^. The data were expressed as mean neuron number ± standard error (SE). The unpaired Student's t-test was used for statistical analysis (GraphPad Prism 5.0c; GraphPad Software, La Jolla, CA, USA)) with p<0.05 considered as statistically significant. The mean coefficient of error for all individual optical fractionator estimates was calculated according to the method of Gundersen and Jensen [16] and was less than 0.07 in all of the analyses.

### Analysis of synapses by puncta analyzer

Synapses were analyzed with Puncta analyser plug-in for ImageJ (1.28u; NIH, Bethesda, MD, USA) as described [17]. Maximum intensity projections of confocal microscopy images (Zeiss LSM 510 Meta/LSM 780, Oberkochen, Germany) from 5 areas per cerebellum, 4–5 animals per group were quantified for the number of synaptic puncta using markers VGAT, VGLUT1, VGLUT2, GABRA6, synapsin 1 and gephyrin. Colocalization of these puncta was also analyzed. The areas analyzed covered different lobules of the cerebellum and were the same in all analyses and animals. The data were expressed as mean puncta number compared to control ± SE. The unpaired Student's t-test was used for statistical analysis (GraphPad Prism 5.0c; GraphPad Software, La Jolla, CA, USA)) with p<0.05 considered as statistically significant.

### Autoradiography of GABA_A_ receptor ligand binding sites

Brains from P7 and P30 *Cstb^−/−^* and control mice (n = 3-4 per genotype) were dissected and frozen in ethanol-isopentane-bath. Sagittal 14 µm thick cryostat sections were mounted on gelatin-chrome alum coated slides. Autoradiographic procedures have been described previously in detail [18], [19].

For [^3^H]muscimol binding to GABA agonist sites, sections were preincubated for 15 min in an ice–water bath in 0.17 M Tris-HCl (pH 7.4). The final incubation in the same buffer was performed with 15 nM [^3^H]muscimol (Perkin-Elmer, Waltham, MA, USA) at 4°C for 30 min. Nonspecific binding was determined with 100 µM GABA (Sigma, St. Louis, MO, USA). After incubation, the sections were washed in ice-cold incubation buffer twice for 30 s, dipped in distilled water and dried in airflow at room temperature.

For [^3^H]Ro15-4513 binding to benzodiazepine sites of the GABA_A_ receptors, sections were preincubated for 15 min in an ice-water bath in 50 mM Tris–HCl, 120 mM NaCl (pH 7.4). The final incubation in the same buffer was performed with 15 nM [^3^H]Ro15-4513 (Perkin-Elmer, Waltham, MA, USA) at 4°C for 60 min. Nonspecific binding was determined with 10 µM Ro15-1788 (flumazenil; Tocris, Bristol, UK). To detect α-6 subunit containing GABA_A_ receptors [20], other GABA_A_ receptor subtypes were blocked with 10 µM diazepam (Sigma, St. Louis, MO, USA). After incubation, the sections were washed with ice-cold incubation buffer for 2×60 s, dipped in distilled water and dried in airflow at room temperature.

The sections were exposed with [^3^H]-plastic standards (GE Healthcare, Little Chalfont, UK) to Kodak Biomax MR films (Eastman Kodak, Rochester, NY, USA) for 2 months. For quantification of binding densities, the imaging plates were analyzed with ImageJ (1.44; NIH, Bethesda, MD, USA) and the data were expressed as mean radioactivity levels (nCi/mg) ± SE with reference to [^3^H]-standards. The unpaired Student's t-test was used for statistical analysis (GraphPad Prism 5.0c; GraphPad Software, La Jolla, CA, USA)) with p<0.05 considered as statistically significant.

## Results

### Altered expression of synaptic genes in Cstb^−/−^ mouse cerebellum at postnatal day 7

The genomewide microarray analysis of P7 cerebella from *Cstb^−/−^* mice revealed 82 differentially expressed probe sets within 61 genes, of which 30 probe sets were upregulated and 52 were downregulated (Table S1 in File S1). The over-represented GO categories are summarized in Table 1 (see also Table S2 in File S1 for complete gene lists within the GO categories). The analyses of molecular and biological function revealed altered expression of genes associated with ion homeostasis, neural networks, neural system development, synaptic function and plasticity, of which GABA_A_ receptor subunits *Gabrd* and *Gabra6* were upregulated. The GO analyses also indicated subtle expression changes of other synaptic genes, such as downregulation of the axon guidance receptor, EPH receptor A7 (*Epha7*), and the cue, slit homolog 2 (*Drosophila*) (*Slit2*). Furthermore, categories within biological processes indicated decreased expression of calcium-dependent synaptic protocadherin beta cluster genes (*Pcdhb22*, *Pcdhb16*, *Pcdhb17*). In GO classification of cellular components, the products of the differentially expressed genes encoded components of ion channel complexes at cell membranes, but also proteins localized to cellular projections and to transport vesicles.

**Table 1:** The GO terms of biological processes, molecular functions and cellular components in P7 *Cstb^−/−^* cerebellum.

**GO ID**	**BIOLOGICAL PROCESS**	**No. genes**	**P-value**
50919	Negative chemotaxis	2	0.0706
10763	Positive regulation of fibroblast migration	2	0.0706
10762	Regulation of fibroblast migration	2	0.0706
21954	Central nervous system neuron development	3	0.0706
22610	Biological adhesion	9	0.0706
21884	Forebrain neuron development	2	0.0706
7155	Cell adhesion	9	0.0706
31290	Retinal ganglion cell axon guidance	2	0.0755
16572	Histone phosphorylation	2	0.0769
42325	Regulation of phosphorylation	8	0.0769
**GO ID**	**MOLECULAR FUNCTION**		
45499	Chemorepellent activity	2	0.0067
16917	GABA receptor activity	2	0.0555
15662	ATPase activity, coupled to transmembrane movement of ions, phosphorylative mechanism	2	0.1391
4175	Endopeptidase activity	4	0.1391
15108	Chloride transmembrane transporter activity	2	0.1391
15399	Primary active transmembrane transporter activity	2	0.1391
4222	Metalloendopeptidase activity	2	0.1391
5254	Chloride channel activity	2	0.1391
**GO ID**	**CELLULAR COMPONENT**		
44297	Cell body	7	0.0279
42995	Cell projection	11	0.0341
30133	Transport vesicle	3	0.0341
71944	Cell periphery	19	0.0341
43005	Neuron projection	8	0.0341
5886	Plasma membrane	19	0.0341
97060	Synaptic membrane	4	0.0478
5657	Replication fork	2	0.0686
43025	Neuronal cell body	5	0.0734
42734	Presynaptic membrane	2	0.0760

### Altered expression of genes involved in cellular biogenesis in Cstb^−/−^ mouse cerebellar granule cells

To get insight into the gene expression changes in neurons, GO categories of 140 differentially expressed probe sets which correspond to 114 known genes were investigated from *Cstb^−/−^* cerebellar granule cells (Table 2, see also Table S3 in File S1 for complete gene lists within the GO categories). Of these, 128 probe sets were upregulated and 12 downregulated (Table S4 in File S1). Biological processes comprised a number of upregulated genes involved in cell cycle, division and growth, e.g. cell division cycle protein 20 homolog (*Cdc20*) and its post-translational modifier, mitotic checkpoint serine/threonine-protein kinase (*Bub1*). Differentially expressed genes linked to cellular architecture, such as genes of kinesin-like protein family (*Kif2c*, *Kif8a*, *Kif22*, *Kif11*) were also detected. Categories of molecular function further indicated an increased expression of growth factors of the CCN family proteins (*Cyr61*/*CCN1*, *Ctgf*/*CCN2*, *Wisp1*/*CCN4*), calcium-dependent annexins (*Anxa1*, *Anxa2*, *Anxa5*) and protease inhibitors (*Timp3*, *Serpine1*, *Serpinh1*), as well as upregulation of several collagen type genes (*Col1a2*, *Col3a1*, *Col4a2*, *Col6a3*). In concordance with the gene function, protein products of majority of the differentially expressed genes localized into nucleus, although gene products associated with cytoskeleton and extracellular matrix were also revealed.

**Table 2:** The GO terms of biological processes, molecular functions and cellular components in P5+2 DIV *Cstb^−/−^* cerebellar granule cells.

**GO ID**	**BIOLOGICAL PROCESS**	**No. genes**	**P-value**
51301	Cell division	23	7.85e-13
7049	Cell cycle	33	1.52e-12
278	Mitotic cell cycle	24	1.65e-12
280	Nuclear division	19	3.64e-12
7067	Mitosis	19	3.64e-12
87	M phase of mitotic cell cycle	19	4.37e-12
48285	Organelle fission	19	8.41e-12
22402	Cell cycle process	26	5.43e-11
22403	Cell cycle phase	23	8.04e-11
279	M phase	19	1.36e-09
**GO ID**	**MOLECULAR FUNCTION**		
5515	Protein binding	62	4.16e-06
5488	Binding	83	2.33e-05
19899	Enzyme binding	20	0.0003
61134	Peptidase regulator activity 3	8	0.0003
4857	Enzyme inhibitor activity	9	0.0005
5539	Glycosaminoglycan binding	7	0.0005
19838	Growth factor binding	6	0.0007
97367	Carbohydrate derivative binding	7	0.0008
5178	Integrin binding	5	0.0008
4866	Endopeptidase inhibitor activity	6	0.0014
**GO ID**	**CELLULAR COMPONENT**		
43232	Intracellular non-membrane-bounded organelle	45	5.62e-09
43228	Non-membrane-bounded organelle	45	5.62e-09
5819	Spindle	12	8.37e-09
780	Condensed nuclear chromosome, centromeric region	6	9.52e-09
31012	Extracellular matrix	15	2.22e-07
5578	Proteinaceous extracellular matrix	14	3.40e-07
44420	Extracellular matrix part	11	3.71e-07
779	Condensed chromosome, centromeric region	6	5.23e-07
778	Condensed nuclear chromosome kinetochore	4	7.38e-07
5581	Collagen	8	7.38e-07

### Altered expression of immune and defense system genes in Cstb^−/−^ mouse cerebellum at postnatal day 30

In P30 cerebella, a total of 82 probe sets corresponding to 67 known genes showed altered expression in *Cstb^−/−^* mice. Of these the majority, 77 probe sets were upregulated, and only five probe sets were downregulated (Table S5 in File S1). Functionally related genes belonging to over-represented GO categories are outlined in Table 3 (see also Table S6 in File S1 for complete gene lists within the GO categories). According to biological processes and molecular function, some of the gene expression patterns predicted changes that were proapoptotic, while others seemed to predict cell survival. Most of the upregulated genes were present in complement pathway and were related to immune and defense response, antigen processing and presentation, cellular stress, cytokine biosynthesis, cell-to-cell signaling and immune cell trafficking, as well as to receptor activity through polysaccharide-, lipid-, immunoglobulin- or protein complex binding. Examples of such genes are complement components and their receptors (*C1qa*, *C1qb*, *C1qc*, *C4b*, *C3ar1*), MHC class 1 molecule, β2-microglobulin (*B2m*), glial fibrillary acidic protein (*Gfap*), chemokine ligands (*Cxcl13*, *Ccl6*), immunoglobulin receptors (*Fcgr3*, *Fcer1g*), and cluster of differentiation antigens (*CD14*, *CD44*, *CD48*, *CD52*). Several proteins coded by the dysregulated genes resided in the integral part of the plasma membrane or were extracellular, while some of the upregulated gene products were prominent to lytic vacuoles, such as hexosaminidase B (*Hexb*), cathepsin D (*Ctsd*), cathepsin H (*Ctsh*), CD68 antigen (*Cd68*), and glucuronidase beta (*Gusb*).

**Table 3:** The GO terms of biological processes, molecular functions and cellular components in P30 *Cstb^−/−^* cerebellum.

**GO ID**	**BIOLOGICAL PROCESS**	**No. genes**	**P-value**
2376	Immune system process	28	2.72e-12
6955	Immune response	21	5.63e-12
2250	Adaptive immune response	12	3.61e-10
6952	Defense response	20	3.61e-10
50896	Response to stimulus	48	3.61e-10
2460	Adaptive immune response based on somatic recombination of immune receptors built from immunoglobulin	11	2.97e-09
2684	Positive regulation of immune system process	15	5.23e-09
50778	Positive regulation of immune response	12	1.98e-08
2449	Lymphocyte mediated immunity	10	1.98e-08
2682	Regulation of immune system process	17	2.30e-08
**GO ID**	**MOLECULAR FUNCTION**		
32403	Protein complex binding	10	7.12e-05
5515	Protein binding	35	0.0009
4872	Receptor activity	12	0.0009
5102	Receptor binding	13	0.0010
4888	Transmembrane signaling receptor activity	10	0.0010
19864	IgG binding	2	0.0011
38023	Signaling receptor activity	10	0.0011
19763	Immunoglobulin receptor activity	2	0.0011
5178	Integrin binding	4	0.0011
5025	Transforming growth factor beta receptor activity, type I	2	0.0011
**GO ID**	**CELLULAR COMPONENT**		
9986	Cell surface	15	7.03e-08
44425	Membrane part	38	7.31e-06
9897	External side of plasma membrane	9	7.92e-06
44421	Extracellular region part	16	7.92e-06
44459	Plasma membrane part	18	1.45e-05
31224	Intrinsic to membrane	34	1.90e-05
5615	Extracellular space	13	2.46e-05
16020	Membrane	43	2.53e-05
5886	Plasma membrane	28	3.31e-05
71944	Cell periphery	28	5.23e-05

### Validation of gene expression changes by real-time quantitative PCR

**Figure 1:**
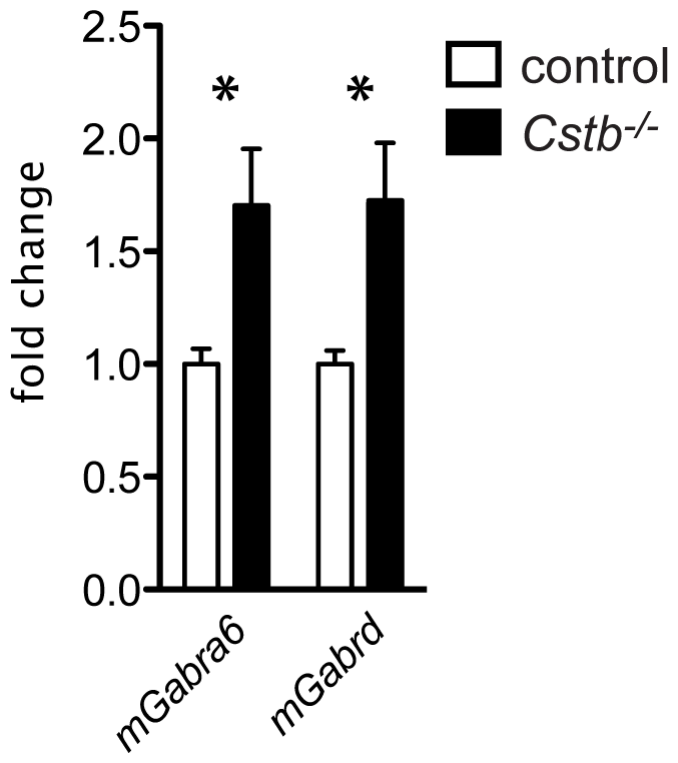
Differential expression of *Gabra6* and *Gabrd*. qPCR shows increased expression of *Gabra6* and *Gabrd* in P7 *Cstb^−/−^* cerebellum compared to the control mice. The data are expressed as a fold change relative to controls ± SE. *, p<0.05.

As gene expression profiling from P7 *Cstb^−/−^* cerebellum implied changes in neuronal functions that could be related to the hyperexcitability and the motor symptoms characteristic for EPM1, the altered GABAergic signaling pathway was selected for further characterization. The differential expression of *Gabra6* and *Gabrd* observed in P7 *Cstb^−/−^* cerebellum was first validated by qPCR. Concordant with the microarray data, increase in expression of *Gabrd* (FC = 1.70±0.2505, p = 0.0144) and *Gabra6* (FC = 1.722±0.2664, p = 0.0174) compared to the control mice was detected (Fig. 1).

### Reduced GABAergic inhibition in Cstb^−/−^ cerebellum

**Figure 2:**
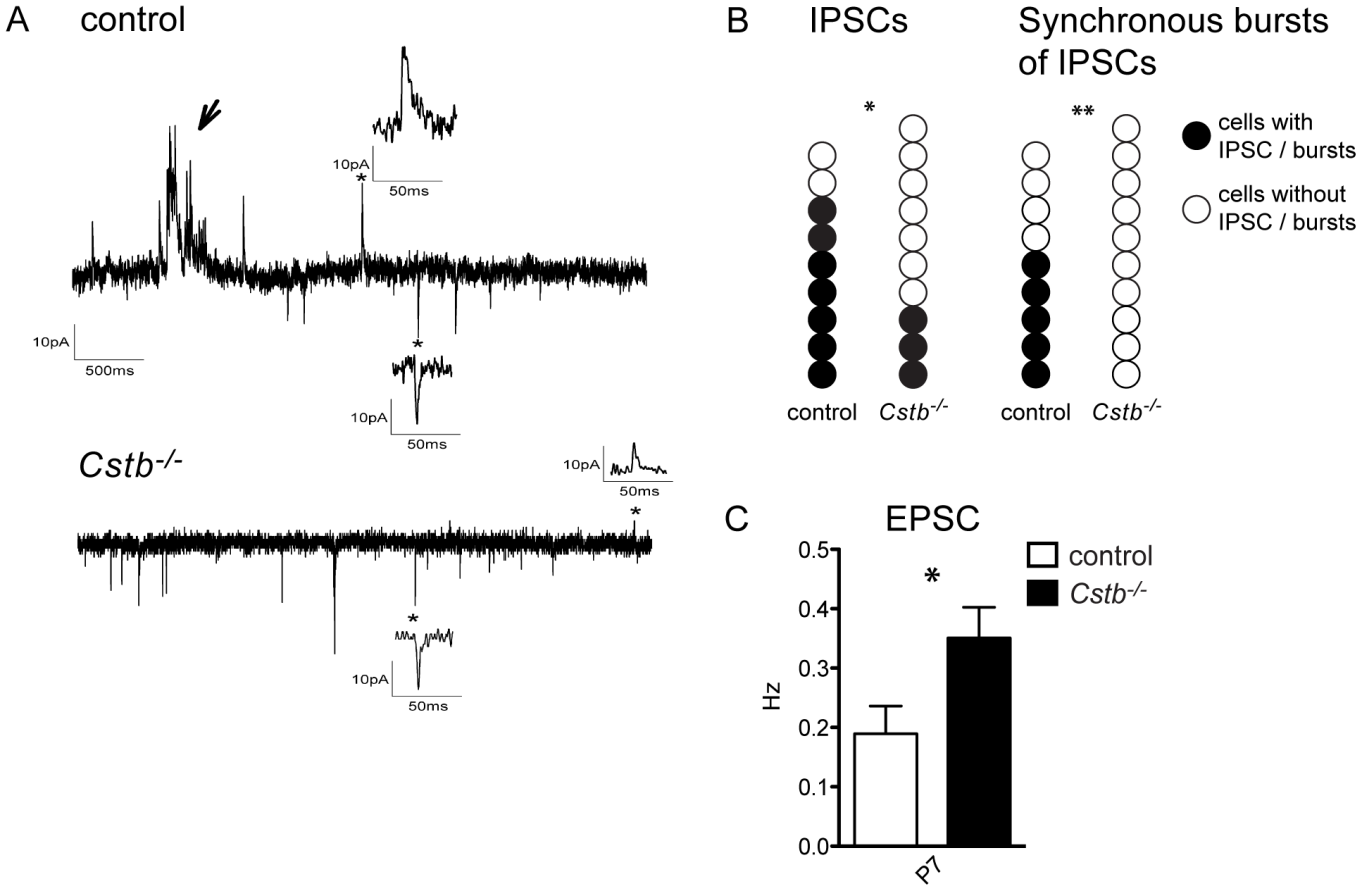
Spontaneous postsynaptic currents in Purkinje cells. **(A)** Representative traces of spontaneous EPSCs and IPSCs in control and *Cstb^−/−^* Purkinje cells. EPSCs are seen as inward currents (downward deflections) and IPSCs are seen as outward currents (upward deflections). Insets show single IPSCs and EPSCs taken from the time points indicated by asterisks. An arrow indicates a synchronous burst of IPSCs seen in control but not in *Cstb^−/−^* Purkinje cells. **(B)** The occurrence of IPSCs and synchronous burst of IPSCs on control and *Cstb^−/−^* Purkinje cells. Individual cells measured are shown as spheres. **(C)** The frequency of EPSCs was significantly higher in *Cstb^−/−^* cells compared to controls (p = 0.034). The data are expressed as mean frequency (Hz) ± SE. *, p<0.05; **, p<0.01.

Next, to study the balance between excitation and inhibition in *Cstb^−/−^* mouse cerebellum, the occurrence of spontaneous excitatory and inhibitory post-synaptic currents (EPSCs and IPSCs) was measured in the somatic area of *Cstb^−/−^* and control Purkinje cells, the output neurons from the cerebellar cortex (Fig. 2A). IPSCs could be recorded in seven out of nine control cells, whereas IPSCs were seen only in three out of ten *Cstb^−/−^* cells (p = 0.037; chi-square 4.34, d.f.  = 1) (Fig. 2B). Further, the IPSC frequency in *Cstb^−/−^* cells was lower than in control cells although this did not reach statistical significance (0.238±0.125 Hz for control vs. 0.054±0.0204 Hz for *Cstb^−/−^*, p = 0.397). Moreover, bursts of synchronous IPSCs seen in five out of nine control cells, were not observed in any of the ten *Cstb^−/−^* cells analyzed (p = 0.006; chi-square 7.54, d.f.  = 1) (Fig. 2B). The IPSC amplitude was lower in *Cstb^−/−^* Purkinje cells compared to controls (32.4±0.5 pA for control vs. 17.4±1.86 pA for *Cstb^−/−^*, p = 0.0435). No differences were seen in decay times between the genotypes (17.4±0.32 ms for control vs. 17.02±1.82 ms for *Cstb^−/−^*). EPSCs could be measured from every control and *Cstb^−/−^* cell and their frequency was significantly higher in *Cstb^−/−^* cells (0.189±0.047 Hz for control vs. 0.351±0.052 Hz for *Cstb^−/−^*, p = 0.0034) (Fig. 2C). There were no differences in EPSC amplitudes or decay times between control and *Cstb^−/−^* Purkinje cells (24.37±2.96 pA, 9.81±0.24 ms for control vs. 26.07±2.79 pA, 10.21±0.51 ms for *Cstb^−/−^*).

### No change in the interneuron number in young Cstb^−/−^ animals

In order to characterize the cellular basis for the imbalance between EPSCs and IPSCs in *Cstb^−/−^* Purkinje cells, the number of inhibitory interneurons in the cerebellar molecular layer was counted. These cells make GABAergic contacts with Purkinje cells. Stereological counting from anti-parvalbumin immunostained (P14) or Nissl-stained sections (P30) showed no significant difference in the number of interneurons in young pre-symptomatic animals (P14: 88 416±5993 for control vs. 70 368±3331 for *Cstb^−/−^*, p = 0.058; P30: 346 848±16 023 for control vs. 335 040±17 406 for *Cstb^−/−^*, p = 0.629). In line with these results, there was no difference between *Cstb^−/−^* and control mice in the amount of GABA-immunopositive cells in cerebellar primary neuron cultures (10.31%±0.88% for control vs. 10.07%±0.46% for *Cstb^−/−^*, p = 0.811).

### Reduced immunopositivity for GABAergic synaptic markers in Cstb^−/−^ mice

**Figure 3:**
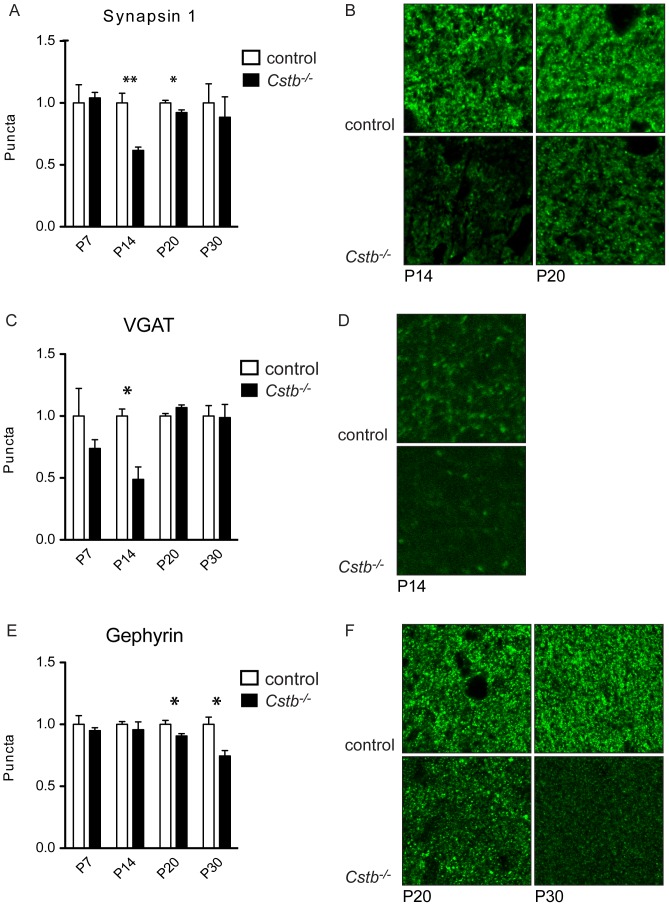
The expression of synapsin 1, VGAT and gephyrin positive synaptic puncta. **(A)** The number of synapsin 1 positive puncta in the molecular layer of *Cstb^−/−^* cerebellum was significantly lower compared to controls at P14 (p = 0.0035) and P20 (p = 0.0447). P7 and P30 animals did not show significant difference. **(B)** Molecular layer of P14 and P20 *Cstb^−/−^* cerebellum shows less synapsin 1 positive puncta compared to control. **(C)** The number of VGAT positive puncta in the molecular layer of *Cstb^−/−^* cerebellum was significantly lower compared to controls at P14 (p = 0.0115). P7, P20 and P30 animals did not show significant difference. **(D)** Molecular layer of P14 *Cstb^−/−^* cerebellum shows less VGAT positive puncta compared to control. **(E)** The number of gephyrin positive puncta in the molecular layer of *Cstb^−/−^* cerebellum was significantly lower compared to controls at P20 (p = 0.0448) and P30 (p = 0.0131). P7 and P14 animals did not show significant difference. **(F)** Molecular layer of P20 and P30 *Cstb^−/−^* cerebellum shows less gephyrin positive puncta compared to control. The data are expressed as mean amount of positive puncta relative to control ± SE. *, p<0.05; **, p<0.01.

Next, *Cstb^−/−^* and control brains were immunolabeled with synaptic markers and the number of immunopositive puncta, corresponding to the number of synaptic terminals, was counted. A significant decrease in the number of puncta positive for synapsin 1, a universal pre-synaptic marker, was observed in P14 and P20 *Cstb^−/−^* cerebellum (Fig. 3A, B). The number of puncta immunoreactive for VGAT, a marker for GABAergic pre-synaptic terminals, was significantly decreased in P14 *Cstb^−/−^* cerebellum (Fig. 3C, D), and gephyrin, a marker for GABAergic post-synaptic terminals, showed decrease in P20 and P30 *Cstb^−/−^* cerebellum (Fig. 3E, F). No differences were seen in GABA_A_ receptor α6 subunit immunopositivity by immunohistochemical or Western blot analyses in P7 or P30 *Cstb^−/−^* cerebellum (data not shown).

Since there was an increase in EPSCs in *Cstb^−/−^* Purkinje cells, excitatory synapses in the cerebellar molecular layer were also counted using markers for vesicular glutamate transporter 1 and 2 (VGLUT1, VGLUT2). No significant differences were detected between *Cstb^−/−^* and control mice, however (data not shown).

### Reduced binding of GABA_A_ receptor ligands to GABA_A_ receptors in Cstb^−/−^ mice

**Figure 4:**
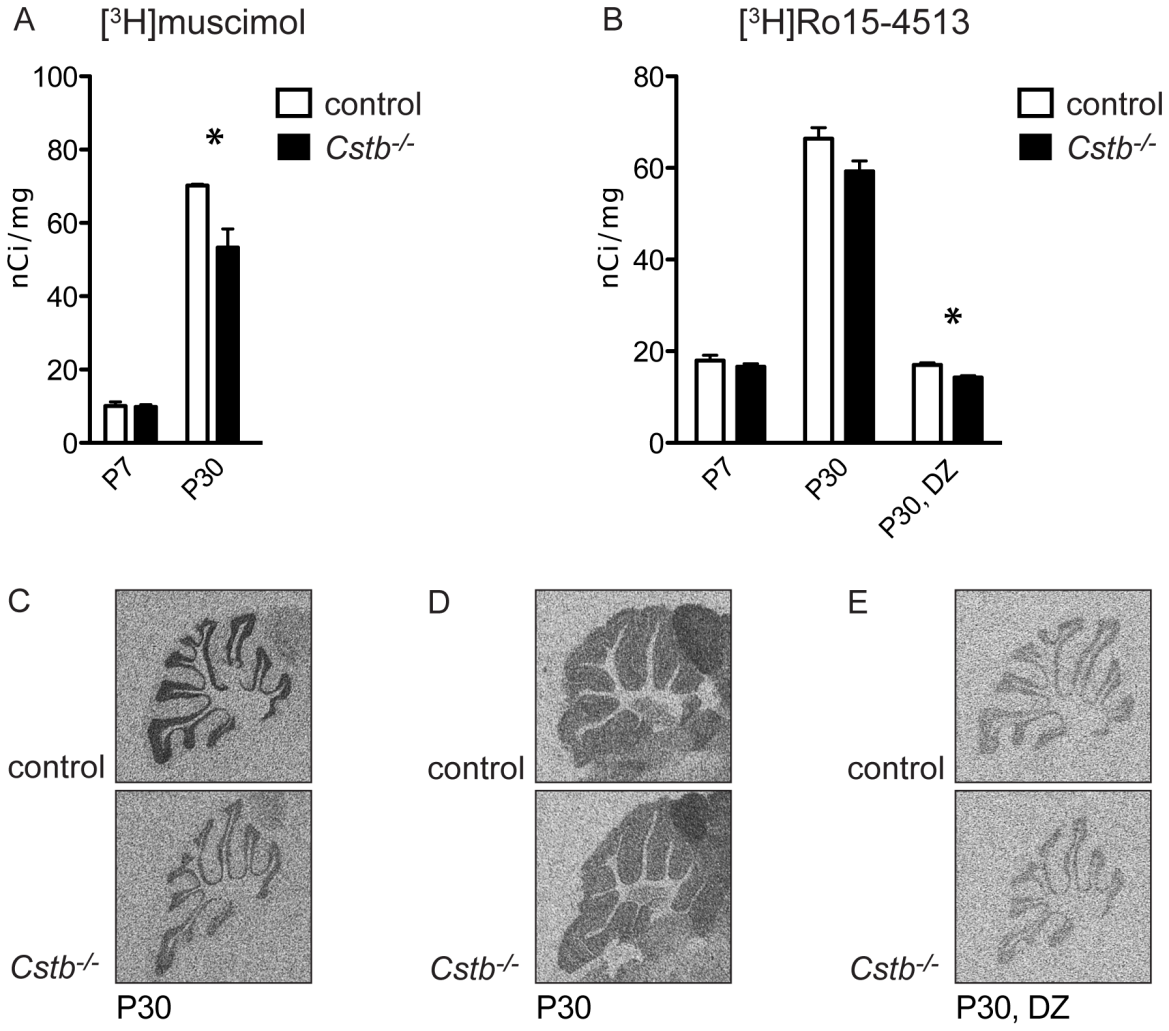
Autoradiography of GABA_A_ receptors. **(A)** Binding of [^3^H]muscimol was significantly reduced in P30 *Cstb^−/−^* cerebellum (p = 0.037). No change was seen at P7. **(B)** Binding of [^3^H]Ro15-4513 did not show significant changes at P7 or at P30. However, when diazepam (DZ) was added to reveal the diazepam-insensitive α6 subunit-dependent GABA_A_ receptor subtype, decreased binding for [^3^H]Ro15-4513 (p = 0.012) was seen. The data are expressed as mean radioactivity levels (nCi/mg) ± SE. *, p<0.05. **(C)** Representative images of [^3^H]muscimol binding to P30 *Cstb^−/−^* and control brain. **(D)** Representative images of [^3^H]Ro15-4513 binding to P30 *Cstb^−/−^* and control brain without and **(E)** in presence of diazepam.

Results from the electrophysiological, interneuron number and immunolabeling studies suggest that the lack of IPSCs in P7 *Cstb^−/−^* cerebellar slices is not due to the reduced number of inhibitory interneurons but alterations in inhibitory synapses *per se*. In order to study this in more detail and to see if the binding of specific ligands to GABA_A_ receptors is affected in *Cstb^−/−^* cerebellum, autoradiography with the GABA_A_ agonist-site ligand [^3^H]muscimol and the benzodiazepine-site ligand [^3^H]Ro15-4513 was performed in P7 and P30 *Cstb^−/−^* and control mice. The autoradiography experiments revealed a slight but significant decrease in [^3^H]muscimol binding (70.22±0.377 nCi/mg for control vs. 53.30±5.051 nCi/mg for *Cstb^−/−^*, p = 0.037) in P30 *Cstb^−/−^* mouse cerebellum (Fig. 4A, C), but not in [^3^H]Ro15-4513 binding (Fig. 4B, D). When [^3^H]Ro15-4513 was incubated together with diazepam at the concentration that blocks the benzodiazepine-sites of all other GABA_A_ receptor subtypes except the α6 subunit-containing ones in the cerebellum, there was a decreased in [^3^H]Ro15-4513 binding to the remaining α6 subunit-containing GABA_A_ receptors (17.01±0.485 nCi/mg for control vs. 14.24±0.409 nCi/mg for *Cstb^−/−^*, p = 0.012) (Fig. 4B, E).

## Discussion

This study is based on the hypothesis that the pathogenetic mechanisms of EPM1 are reflected in altered gene expression patterns. To identify molecular defects associated with CSTB deficiency, we used cerebellum as a model which shows striking pathology in *Cstb^−/−^* mice [4], [6], and is also affected in human EPM1 patients [21]. Global gene expression profiling from cerebella and cultured cerebellar granule cells revealed multiple affected biological pathways that could have a role in the pathogenesis of EPM1 not limited to cerebellum.

Several modest changes in gene expression related to synapse maturation, development and function during postnatal maturation of the brain were detected already in P7 *Cstb^−/−^* cerebellum. GABAergic signaling emerged as a pathway that showed most prominent changes manifesting, for example, as overexpression of GABA_A_ receptor subunits *Gabrd* and *Gabra6*. GABA plays a central role in controlling neuronal development and connectivity, and defective GABAergic signaling in the cerebellum of *Cstb^−/−^* mouse provides biological mechanisms for ataxia in these mice [22]. However, when extrapolated to other brain regions affected in EPM1, alterations in GABAergic signaling could also contribute to hyperexcitability, manifested as severe myoclonus and seizures. In the cerebral cortex, alterations in GABA signaling may cause seizures, and *vice versa*, seizures can alter GABA signaling [23], [24]. Many GABA-related mutations are known to cause epilepsy in early life, and for example the analysis of a conditional mutant with disrupted GABA_A_ signaling has implied a developmental period when disrupted GABA signaling may be critical for later ictogenesis and epileptogenesis in addition to having developmental consequences [25].

Another example of a synapse-related gene showing altered expression in *Cstb^−/−^* cerebellum was the downregulated *Epha7* that belongs to the ephrin receptor subfamily of the protein-tyrosine kinases, which through their surface associated ligands, ephrins, play a role in the initiation of new synaptic contacts, in dendritic spine morphology and in modulation of the mature synapses [26]. The target recognition and axon guidance factor *Slit2*, which also showed reduced expression, is known to stimulate axonal elongation and branching, and to modulate neurogenesis in developing brain and may regulate axon-axon adhesion mediated by cadherins [27]-[29]. Moreover, several genes encoding protocadherins were downregulated at P7. Synaptic protocadherins are central molecules in Ca^2+^-regulated cell-adhesion and they are expressed especially during neural development being implicated in synaptic recognition and stabilization [30].

While the microarray data in *Cstb^−/−^* cerebellum at P7 pinpointed defects in processes related to synaptogenesis and synaptic maturation with alterations in the GABAergic signaling highlighted, *Cstb^−/−^* neurons exposed upregulation of several cell cycle-related genes. Re-entry of neurons to cell cycle after differentiation would lead to apoptotic cell death and neurodegeneration, rather than cell division [31]. We did not, however, detect elevated cell death in *Cstb^−/−^* granule cell cultures, at least when the neurons were cultured for two days. We cannot, though, exclude the possibility that an activation of cell cycle related genes in *Cstb^−/−^* granule cells reflects their dysfunction eventually leading to cell death. Postmitotic neurons express also cell cycle proteins, which are involved in neuronal morphogenesis and in the regulation of pre-synaptic differentiation, such as CDC20 (upregulated in our data) through interaction with a multiprotein anaphase-promoting complex [32]-[34]. Upregulation of cell cycle genes in *Cstb^−/−^* granule neurons may also imply to functions of CSTB in the nucleus. CSTB localizes to the cytoplasm, where it associates with lysosomes, but it has also been detected in the nucleus of dividing cells [35], [36]. In the nucleus, CSTB has been shown to interact with histones and cathepsin L and to regulate cell cycle progression into the S phase [37]. It is tempting to speculate that the synaptic changes may, at least partly, be mediated by cathepsins in the cells, including the synaptic sites and the nucleus. Imbalance of cathepsin regulation in the synapses could lead to morphological and functional changes [38]. On the other hand, disturbed nuclear function of CSTB, which is at least partially mediated by cathepsin regulation, could result in consequent alterations in the transcriptional regulation of synaptic proteins [37], [39]. Synaptic functions are highly dependent on functional cytoskeleton that regulates intracellular transport and protein turnover in the synapses. *Cstb^−/−^* granule neurons revealed also significant overexpression of several genes encoding kinesins, CCN family of proteins and annexins, that have been associated in axonal transport, nuclear division, mitosis, extracellular matrix production, apoptosis and GABAergic signaling [40]–[44]. Taken together, the data from *Cstb^−/−^* granule cells propose neuron specific alterations in processes central to neuronal function and architecture and emphasizes nuclear functions of CSTB.

As the gene expression data from P7 *Cstb^−/−^* cerebellum and neurons alluded to alterations in synaptic functions, which could contribute to the neuronal hyperexcitability and characteristic motor symptoms seen in EPM1 patients (see above), we selected GABAergic signaling for more detailed characterization. At P7, mRNA levels of granule cell specific GABA_A_ receptor subtypes α6 and δ were elevated, although not highly enough to be able to detect at protein level. However, electrophysiological analyses of *Cstb^−/−^* mouse cerebellar slices showed a shift of balance towards decreased inhibition and increased excitation in the Purkinje cells. Thus, the detected upregulation of GABA_A_ receptor subunit mRNAs at P7 *Cstb^−/−^* mouse could reflect a compensatory change in gene expression to decrease the excitatory neurotransmission from granule cells to Purkinje cells, especially as the GABA_A_ receptor subunit mRNAs, which were upregulated, were those for the α6 and δ subunits that mostly form extrasynaptic receptors responsible for tonic inhibition [45]. On the other hand, at P30, we found reduced ligand binding to α6 and δ subunit-containing GABA_A_ receptors [18], [46] indicating alterations in functional post-synaptic and extrasynaptic receptors in *Cstb^−/−^* mouse cerebellum at this age. Whether the changes in GABA_A_ receptor function could be due to e.g. availability of different receptor subtypes in the membrane needs to be investigated.

A decrease in the number of GABAergic terminals leading to reduced GABA inhibition has previously been reported in cerebral cortex of aged *Cstb^−/−^* mice [47]. The loss of interneurons in aged mice can further reduce the GABA inhibition (our unpublished observation and [47]). Consistent with the mouse data, a loss of pre-synaptic GABAergic marker VGAT was also detected in the brain of a fully symptomatic EPM1 patient [47]. Our data in young mice show a significant decrease in VGAT immunoreactivity along with the reduction of synapsin 1 positive pre-synaptic as well as gephyrin positive post-synaptic terminals, indicating that there are defects in GABAergic synapses in *Cstb^−/−^* cerebellum already at the pre-symptomatic stage, before the loss of GABAergic interneurons is detectable.

In contrast to the findings at P7, changes in the expression of genes reflecting synaptic functions by GO analysis are no longer observed at P30, or they may be hidden by significant enrichment for genes and pathways which pinpoint activation of inflammatory processes. Earlier reports from *Cstb^−/−^* mice cerebella have shown microglial activation in presymptomatic mice followed by neuronal death and volume loss from two months of age onward [4], [6]. Therefore, elevated expression of inflammatory genes at P30 most likely reflect response to activated glial cells and neuroinflammation, which together with neuronal dysfunction and death has an important role in progression of the disease. Glial activation and oxidative stress [5], [6] might further promote the hyperexcitability in *Cstb^−/−^* mice, as glial derived proinflammatory chemokines and cytokines, highly expressed also in P30 *Cstb^−/−^* mice, have been found to reduce the seizure threshold and may thus contribute to recurrent excitation in epilepsy [48].

In conclusion, we provide the first evidence of gene expression changes in pre-symptomatic and young symptomatic *Cstb^−/−^* mice. Although there is no significant overlap with the differentially expresses genes at P7 and P30, GO analyses revealed alterations in several functional categories, which may contribute to EPM1. Our data indicate that pre- and post-synaptic changes in inhibitory GABAergic synapses could result in imbalance between excitation and inhibition in *Cstb^−/−^* mouse cerebellum already before the disease symptoms occur, which may be augmented by inflammatory processes in the symptomatic phase.

## Supporting Information

File S1 **Table S1.** The gene expression changes in P7 *Cstb^−/−^* cerebellum. Fold change with cutoff 1.3 and p<0.05 was used. **Table S2.** The GO terms of biological process, molecular function and cellular component in P7 *Cstb^−/−^* cerebellum. N indicates the number of genes and P the adjusted p-value for the enrichment. **Table S3.** The GO terms of biological process, molecular function and cellular component in *Cstb^−/−^* P5+2 cerebellar granule cells. N indicates the number of genes and P the adjusted p-value for the enrichment. **Table S4.** The gene expression changes in *Cstb^−/−^* P5+2 cerebellar granule cells. Fold change with cutoff 1.3 and p<0.01 was used. **Table S5.** The gene expression changes in P30 *Cstb^−/−^* cerebellum. Fold change with cutoff 1.3 and p<0.05 was used. **Table S6.** The GO terms of biological process, molecular function and cellular component in P30 *Cstb^−/−^* cerebellum. N indicates the number of genes and P is the adjusted p-value for the enrichment.
(PDF)
