# Supplementary material for: Gene Expression Alterations in the Cerebellum and Granule Neurons of Cstb−/− Mouse Are Associated with Early Synaptic Changes and Inflammation
Source: PLoS One. 2014 Feb 27;9(2):e89321. doi: 10.1371/journal.pone.0089321 (PMC3937333; doi:10.1371/journal.pone.0089321)
Supplement: File S1 — Table S1. The gene expression changes in P7 Cstb−/− cerebellum. Fold change with cutoff 1.3 and p<0.05 was used. Table S2. The GO terms of biological process, molecular function and cellular component in P7 Cstb−/− cerebellum. N indicates the number of genes and P the adjusted p-value for the enrichment. Table S3. The GO terms of biological process, molecular function and cellular component in Cstb−/− P5+2 cerebellar granule cells. N indicates the number of genes and P the adjusted p-value for the enrichment. Table S4. The gene expression changes in Cstb−/− P5+2 cerebellar granule cells. Fold change with cutoff 1.3 and p<0.01 was used. Table S5. The gene expression changes in P30 Cstb−/− cerebellum. Fold change with cutoff 1.3 and p<0.05 was used. Table S6. The GO terms of biological process, molecular function and cellular component in P30 Cstb−/− cerebellum. N indicates the number of genes and P is the adjusted p-value for the enrichment. (PDF) [file pone.0089321.s001.pdf]

Table S1. The gene expression changes in P7 *Cstb*$^{-/-}$ cerebellum. Fold change with cutoff 1.3 and $p < 0.05$ was used.

Probe Set ID	Gene Symbol	Fold change([KO]/[WT])	Unigene(Avadis)
1424343_a_at	Eif1a	-1.31	Mm.262037
1452757_s_at	Hba-a1 /// Hba-a2	-1.31	Mm.196110
1416934_at	Mtm1	-1.31	Mm.423278
1420919_at	Sgk3	-1.31	Mm.336410
1430177_at	Ube2b	-1.31	Mm.397314
1418827_at	Thex1	-1.32	Mm.207534
1452380_at	Epha7	-1.32	Mm.257266
1428361_x_at	Hba-a1 /// Hba-a2	-1.32	Mm.196110
1437434_a_at	Gpr177	-1.33	Mm.6766
1437500_at	Noc3l	-1.34	Mm.86482
1456292_a_at	Vim	-1.34	Mm.268000
1449122_at	Ubxn2b	-1.34	Mm.427021
1438729_at	Sox1	-1.35	Mm.39088
1424659_at	Slit2	-1.35	Mm.289739
1452458_s_at	Ppil5	-1.35	Mm.28847
1419123_a_at	Pdgfc	-1.36	Mm.331089
1453282_at	Cxadr	-1.36	Mm.66222
1449186_at	Bag4	-1.36	Mm.118400
1429712_at	Etohi1	-1.36	Mm.455970
1428694_at	Mir17hg	-1.37	Mm.34859
1456625_at	Aasdhppt	-1.37	Mm.33970
1428960_at	Enkur	-1.37	Mm.27658
1431057_a_at	Prss23	-1.37	Mm.250438
1428329_a_at	Ift80	-1.37	Mm.389451
1449373_at	Dnajc3a	-1.38	Mm.12616
1436921_at	Atp7a	-1.38	Mm.254297
1450677_at	Chek1	-1.38	Mm.16753
1439836_at	Asb15	-1.39	Mm.160150
1418941_at	Pcdhb22	-1.39	Mm.466998
1417184_s_at	Hbb-b1 /// Hbb-b2	-1.41	Mm.288567
1424589_s_at	Rnpc3	-1.42	Mm.316928
1441358_at	Pcdhb16	-1.43	Mm.197441
1434423_at	Gulp1	-1.45	Mm.133132
1426367_at	Cab39l	-1.45	Mm.179091
1423516_a_at	Nid2	-1.46	Mm.20348
1432052_at	Exosc1	-1.46	Mm.289086
1448021_at	Fam46c	-1.46	Mm.440167
1424083_at	Rod1	-1.46	Mm.389581
1416529_at	Emp1	-1.47	Mm.182785
1434719_at	A2m	-1.48	Mm.30151
1423821_at	Tmem168	-1.48	Mm.254845
1455715_at	Gm1976	-1.48	Mm.426514
1436473_at	Zfp248	-1.49	Mm.212572

1448933_at	Pcdhb17	-1.49	Mm.87553
1437671_x_at	Prss23	-1.51	Mm.250438
1417714_x_at	Hba-a1 /// Hba-a2	-1.58	Mm.196110
1428987_at	Dynlrb2	-1.59	Mm.23114
1454838_s_at	AW548124	-1.61	Mm.311974
1433462_a_at	Pi4k2a	-1.78	Mm.117037
1422507_at	Cstb	-4.70	Mm.6095
1422506_a_at	Cstb	-7.68	Mm.6095
1458697_at	NA	1.30	Mm.385245
1446360_at	LOC100048866	1.31	Mm.182709
1458230_at	NA	1.31	Mm.215663
1421286_a_at	Atp4a	1.31	Mm.12821
1446537_at	NA	1.31	Mm.215494
1444675_at	AL023051	1.32	Mm.181064
1424944_at	Pcp2	1.32	Mm.440882
1457763_at	Gabrd	1.32	Mm.388925
1439283_at	Osbpl9	1.33	Mm.366315
1446540_at	NA	1.33	Mm.448248
1441360_at	NA	1.34	Mm.102815
1455785_at	Kcna1	1.34	Mm.40424
1438217_at	A2bp1	1.35	Mm.370334
1418744_s_at	Tesc	1.35	Mm.273285
1446524_at	NA	1.36	Mm.213327
1446247_at	Adamts18	1.36	Mm.167471
1448686_at	Il16	1.37	Mm.10137
1455270_at	Adam11	1.38	Mm.89854
1417391_a_at	Il16	1.39	Mm.10137
1453456_at	2900084O13Rik	1.40	Mm.158938
1440357_at	Mirlet7b	1.40	Mm.33117
1445656_at	NA	1.40	Mm.432213
1459723_at	Zdhhc22	1.43	Mm.185890
1438752_at	A230058F20Rik	1.43	Mm.74679
1420354_at	Cnnm1	1.43	Mm.329864
1448107_x_at	Klk1	1.44	Mm.142722
1453060_at	Rgs8	1.48	Mm.379143
1437861_s_at	Prkce	1.48	Mm.24614
1449980_a_at	Gabrd	1.55	Mm.388925
1415837_at	Klk1	1.62	Mm.142722
1417653_at	Pvalb	1.67	Mm.2766
1417121_at	Gabra6	1.94	Mm.4915

Table S2. The GO terms of biological process, molecular function and cellular component in P7 *Cstb*$^{-/-}$ cerebellum. N indicates the number of genes and P the adjusted p-value for the enrichment.

BIOLOGICAL PROCESS

**Probe ID**	**Gene symbol**	**Gene title**	**Fold change**
**Negative chemotaxis GO:50919 (N=2; P=0.0706)**			
1424659_at	Slit2	slit homolog 2 (*Drosophila*)	-1.35
1452380_at	Epha7	Eph receptor A7	-1.32
**Positive regulation of fibroblast migration GO:10763 (N=2; P=0.0706)**			
1449186_at	Bag4	BCL2-associated athanogene 4	-1.36
1437861_s_at	Prkce	protein kinase C, epsilon	1.48
**Regulation of fibroblast migration GO:10762 (N=2; P=0.0706)**			
1449186_at	Bag4	BCL2-associated athanogene 4	-1.36
1437861_s_at	Prkce	protein kinase C, epsilon	1.48
**Central nervous system neuron development GO:21954 (N=3;P=0.0706)**			
1438729_at	Sox1	SRY-box containing gene 1	-1.35
1436921_at	Atp7a	ATPase, Cu++ transporting, alpha polypeptide	-1.38
1424659_at	Slit2	slit homolog 2 (*Drosophila*)	-1.35
**Biological adhesion GO:22610 (N=9;P=0.0706)**			
1453282_at	Cxadr	coxsackie virus and adenovirus receptor	-1.36
1424659_at	Slit2	slit homolog 2 (*Drosophila*)	-1.35
1452380_at	Epha7	Eph receptor A7	-1.32
1448933_at	Pcdhb17	protocadherin beta 17	-1.49
1449186_at	Bag4	BCL2-associated athanogene 4	-1.36
1418941_at	Pcdhb22	protocadherin beta 22	-1.39
1437861_s_at	Prkce	protein kinase C, epsilon	1.48
1441358_at	Pcdhb16	protocadherin beta 16	-1.43

1423516_a_at	Nid2	nidogen 2	-1.46
**Forebrain neuron development GO:21884 (N=2; P=0.0706)**			
1438729_at	Sox1	SRY-box containing gene 1	-1.35
1436921_at	Atp7a	ATPase, Cu++ transporting, alpha polypeptide	-1.38
**Cell adhesion GO:7155 (N=9; P=0.0706)**			
1453282_at	Cxadr	coxsackie virus and adenovirus receptor	-1.36
1424659_at	Slit2	slit homolog 2 (*Drosophila*)	-1.35
1452380_at	Epha7	Eph receptor A7	-1.32
1448933_at	Pcdhb17	protocadherin beta 17	-1.49
1449186_at	Bag4	BCL2-associated athanogene 4	-1.36
1418941_at	Pcdhb22	protocadherin beta 22	-1.39
1437861_s_at	Prkce	protein kinase C, epsilon	1.48
1441358_at	Pcdhb16	protocadherin beta 16	-1.43
1423516_a_at	Nid2	nidogen 2	-1.46
**Retinal ganglion cell axon guidance GO:31290 (N=2; P=0.0755)**			
1424659_at	Slit2	slit homolog 2 (*Drosophila*)	-1.35
1452380_at	Epha7	Eph receptor A7	-1.32
**Histone phosphorylation GO:16572 (N=2; P=0.0769)**			
1450677_at	Chek1	checkpoint kinase 1	-1.38
1430177_at	Ube2b	ubiquitin-conjugating enzyme E2B	-1.31
**Regulation of phosphorylation GO:42325 (N=8; P=0.0769)**			
1436921_at	Atp7a	ATPase, Cu++ transporting, alpha polypeptide	-1.38
1419123_a_at	Pdgfc	platelet-derived growth factor, C polypeptide	-1.36
1424659_at	Slit2	slit homolog 2 (*Drosophila*)	-1.35
1452380_at	Epha7	Eph receptor A7	-1.32
1449186_at	Bag4	BCL2-associated athanogene 4	-1.36
1449373_at	Dnajc3	DnaJ (Hsp40) homolog, subfamily C, member 3	-1.38

1437861_s_at	Prkce	protein kinase C, epsilon	1.48
1430177_at	Ube2b	ubiquitin-conjugating enzyme E2B	-1.31

MOLECULAR FUNCTION

**Chemorepellent activity GO:45499 (N=2; P=0.0067)**			
1424659_at	Slit2	slit homolog 2 (*Drosophila*)	-1.35
1452380_at	Epha7	Eph receptor A7	-1.32
**GABA receptor activity GO:16917 (N=2; P=0.0555)**			
1457763_at	Gabrd	gamma-aminobutyric acid (GABA) A receptor, subunit delta	1.32
1449980_a_at	Gabrd	gamma-aminobutyric acid (GABA) A receptor, subunit delta	1.55
1417121_at	Gabra6	gamma-aminobutyric acid (GABA) A receptor, subunit alpha 6	1.94
**ATPase activity, coupled to transmembrane movement of ions, phosphorylative mechanism GO:15662 (N=2; P=0.1391)**			
1436921_at	Atp7a	ATPase, Cu++ transporting, alpha polypeptide	-1.38
1421286_a_at	Atp4a	ATPase, H+/K+ exchanging, gastric, alpha polypeptide	1.31
**Endopeptidase activity GO:4175 (N=4; P=0.1391)**			
1448107_x_at	Klk1	kallikrein 1	1.44
1415837_at	Klk1	kallikrein 1	1.62
1446247_at	Adamts18	a disintegrin-like and metallopeptidase (reprolysin type) with thrombospondin type 1 motif, 18	1.36
1437671_x_at	Prss23	protease, serine, 23	-1.51
1431057_a_at	Prss23	protease, serine, 23	-1.37
1455270_at	Adam11	a disintegrin and metallopeptidase domain 11	1.38
**Chloride transmembrane transporter activity GO:15108 (N=2; P=0.1391)**			
1457763_at	Gabrd	gamma-aminobutyric acid (GABA) A receptor, subunit delta	1.32
1449980_a_at	Gabrd	gamma-aminobutyric acid (GABA) A receptor, subunit delta	1.55
1417121_at	Gabra6	gamma-aminobutyric acid (GABA) A receptor, subunit alpha 6	1.94
**Primary active transmembrane transporter activity GO:15399 (N=2; P=0.1391)**			

1436921_at	Atp7a	ATPase, Cu++ transporting, alpha polypeptide	-1.38
1421286_a_at	Atp4a	ATPase, H+/K+ exchanging, gastric, alpha polypeptide	1.31
**Metalloendopeptidase activity GO:4222 (N=2; P=0.1391)**			
1446247_at	Adamts18	a disintegrin-like and metallopeptidase (reprolysin type) with thrombospondin type 1 motif, 18	1.36
1455270_at	Adam11	a disintegrin and metallopeptidase domain 11	1.38
**Chloride channel activity GO:5254 (N=2; P=0.1391)**			
1457763_at	Gabrd	gamma-aminobutyric acid (GABA) A receptor, subunit delta	1.32
1449980_a_at	Gabrd	gamma-aminobutyric acid (GABA) A receptor, subunit delta	1.55
1417121_at	Gabra6	gamma-aminobutyric acid (GABA) A receptor, subunit alpha 6	1.94
**CELLULAR COMPONENT**			
**Cell body GO:44297 (N=7; P=0.0279)**			
1436921_at	Atp7a	ATPase, Cu++ transporting, alpha polypeptide	-1.38
1455785_at	Kcna1	potassium voltage-gated channel, shaker-related subfamily, member 1	1.34
1417121_at	Gabra6	gamma-aminobutyric acid (GABA) A receptor, subunit alpha 6	1.94
1433462_a_at	Pi4k2a	phosphatidylinositol 4-kinase type 2 alpha	-1.78
1453282_at	Cxadr	coxsackie virus and adenovirus receptor	-1.36
1456292_a_at	Vim	vimentin	-1.34
1452380_at	Epha7	Eph receptor A7	-1.32
**Cell projection GO:42995 (N=11; P=0.0341)**			
1417653_at	Pvalb	parvalbumin	1.67
1416934_at	Mtm1	X-linked myotubular myopathy gene 1	-1.31
1436921_at	Atp7a	ATPase, Cu++ transporting, alpha polypeptide	-1.38
1455785_at	Kcna1	potassium voltage-gated channel, shaker-related subfamily, member 1	1.34
1417121_at	Gabra6	gamma-aminobutyric acid (GABA) A receptor, subunit alpha 6	1.94
1453282_at	Cxadr	coxsackie virus and adenovirus receptor	-1.36
1452380_at	Epha7	Eph receptor A7	-1.32

1428329_a_at	Ift80	intraflagellar transport 80	-1.37
1433462_a_at	Pi4k2a	phosphatidylinositol 4-kinase type 2 alpha	-1.78
1428960_at	Enkur	enkurin, TRPC channel interacting protein	-1.37
1456292_a_at	Vim	vimentin	-1.34
**Transport vesicle GO:30133 (N=3; P=0.0341)**			
1436921_at	Atp7a	ATPase, Cu++ transporting, alpha polypeptide	-1.38
1433462_a_at	Pi4k2a	phosphatidylinositol 4-kinase type 2 alpha	-1.78
1423821_at	Tmem168	transmembrane protein 168	-1.48
**Cell periphery GO:71944 (N=19; P=0.0341)**			
1457763_at	Gabrd	gamma-aminobutyric acid (GABA) A receptor, subunit delta	1.32
1449980_a_at	Gabrd	gamma-aminobutyric acid (GABA) A receptor, subunit delta	1.55
1416934_at	Mtm1	X-linked myotubular myopathy gene 1	-1.31
1448686_at	Il16	interleukin 16	1.37
1417391_a_at	Il16	interleukin 16	1.39
1436921_at	Atp7a	ATPase, Cu++ transporting, alpha polypeptide	-1.38
1455785_at	Kcna1	potassium voltage-gated channel, shaker-related subfamily, member 1	1.34
1417121_at	Gabra6	gamma-aminobutyric acid (GABA) A receptor, subunit alpha 6	1.94
1416529_at	Emp1	epithelial membrane protein 1	-1.47
1453282_at	Cxadr	coxsackie virus and adenovirus receptor	-1.36
1452380_at	Epha7	Eph receptor A7	-1.32
1449186_at	Bag4	BCL2-associated athanogene 4	-1.36
1418941_at	Pcdhb22	protocadherin beta 22	-1.39
1441358_at	Pcdhb16	protocadherin beta 16	-1.43
1420354_at	Cnnm1	cyclin M1	1.43
1424659_at	Slit2	slit homolog 2 (*Drosophila*)	-1.35
1448933_at	Pcdhb17	protocadherin beta 17	-1.49
1433462_a_at	Pi4k2a	phosphatidylinositol 4-kinase type 2 alpha	-1.78

1421286_a_at	Atp4a	ATPase, H+/K+ exchanging, gastric, alpha polypeptide	1.31
1430177_at	Ube2b	ubiquitin-conjugating enzyme E2B	-1.31
1437861_s_at	Prkce	protein kinase C, epsilon	1.48
**Neuron projection GO:43005 (N=8; P=0.0341)**			
1417653_at	Pvalb	parvalbumin	1.67
1436921_at	Atp7a	ATPase, Cu++ transporting, alpha polypeptide	-1.38
1455785_at	Kcna1	potassium voltage-gated channel, shaker-related subfamily, member 1	1.34
1417121_at	Gabra6	gamma-aminobutyric acid (GABA) A receptor, subunit alpha 6	1.94
1433462_a_at	Pi4k2a	phosphatidylinositol 4-kinase type 2 alpha	-1.78
1453282_at	Cxadr	coxsackie virus and adenovirus receptor	-1.36
1456292_a_at	Vim	vimentin	-1.34
1452380_at	Epha7	Eph receptor A7	-1.32
**Plasma membrane GO:5886 (N=19; P=0.0341)**			
1457763_at	Gabrd	gamma-aminobutyric acid (GABA) A receptor, subunit delta	1.32
1449980_a_at	Gabrd	gamma-aminobutyric acid (GABA) A receptor, subunit delta	1.55
1416934_at	Mtm1	X-linked myotubular myopathy gene 1	-1.31
1448686_at	Il16	interleukin 16	1.37
1417391_a_at	Il16	interleukin 16	1.39
1436921_at	Atp7a	ATPase, Cu++ transporting, alpha polypeptide	-1.38
1455785_at	Kcna1	potassium voltage-gated channel, shaker-related subfamily, member 1	1.34
1417121_at	Gabra6	gamma-aminobutyric acid (GABA) A receptor, subunit alpha 6	1.94
1416529_at	Emp1	epithelial membrane protein 1	-1.47
1453282_at	Cxadr	coxsackie virus and adenovirus receptor	-1.36
1452380_at	Epha7	Eph receptor A7	-1.32
1449186_at	Bag4	BCL2-associated athanogene 4	-1.36
1418941_at	Pcdhb22	protocadherin beta 22	-1.39
1441358_at	Pcdhb16	protocadherin beta 16	-1.43

1420354_at	Cnnm1	cyclin M1	1.43
1424659_at	Slit2	slit homolog 2 (*Drosophila*)	-1.35
1448933_at	Pcdhb17	protocadherin beta 17	-1.49
1433462_a_at	Pi4k2a	phosphatidylinositol 4-kinase type 2 alpha	-1.78
1421286_a_at	Atp4a	ATPase, H+/K+ exchanging, gastric, alpha polypeptide	1.31
1430177_at	Ube2b	ubiquitin-conjugating enzyme E2B	-1.31
1437861_s_at	Prkce	protein kinase C, epsilon	1.48
**Synaptic membrane GO:97060 (N=4; P=0.0478)**			
1457763_at	Gabrd	gamma-aminobutyric acid (GABA) A receptor, subunit delta	1.32
1449980_a_at	Gabrd	gamma-aminobutyric acid (GABA) A receptor, subunit delta	1.55
1417121_at	Gabra6	gamma-aminobutyric acid (GABA) A receptor, subunit alpha 6	1.94
1433462_a_at	Pi4k2a	phosphatidylinositol 4-kinase type 2 alpha	-1.78
1452380_at	Epha7	Eph receptor A7	-1.32
**Replication fork GO:5657 (N=2; P=0.0686)**			
1450677_at	Chek1	checkpoint kinase 1	-1.38
1430177_at	Ube2b	ubiquitin-conjugating enzyme E2B	-1.31
**Neuronal cell body GO:43025 (N=5; P=0.0734)**			
1436921_at	Atp7a	ATPase, Cu++ transporting, alpha polypeptide	-1.38
1455785_at	Kcna1	potassium voltage-gated channel, shaker-related subfamily, member 1	1.34
1417121_at	Gabra6	gamma-aminobutyric acid (GABA) A receptor, subunit alpha 6	1.94
1433462_a_at	Pi4k2a	phosphatidylinositol 4-kinase type 2 alpha	-1.78
1452380_at	Epha7	Eph receptor A7	-1.32
**Presynaptic membrane GO:42734 (N=2;P=0.0760)**			
1417121_at	Gabra6	gamma-aminobutyric acid (GABA) A receptor, subunit alpha 6	1.94
1433462_a_at	Pi4k2a	phosphatidylinositol 4-kinase type 2 alpha	-1.78

Table S3. The GO terms of biological process, molecular function and cellular component in *Cstb*$^{-/-}$ P5+2 cerebellar granule cells. N indicates the number of genes and P the adjusted p-value for the enrichment.

BIOLOGICAL PROCESS

Probe ID	Gene symbol	Gene title	Fold change
**Cell division GO:51301 (N=23; P=7.85e-13)**			
1416309_at	Nusap1	nucleolar and spindle associated protein 1	5.09
1422851_at	Hmga2	high mobility group AT-hook 2	4.21
1450780_s_at	Hmga2	high mobility group AT-hook 2	4.07
1422441_x_at	Cdk4	cyclin-dependent kinase 4	1.75
1424105_a_at	Pttg1	pituitary tumor-transforming gene 1	2.86
1423775_s_at	Prc1	protein regulator of cytokinesis 1	3.17
1450920_at	Ccnb2	cyclin B2	5.05
1428481_s_at	Cdca8	cell division cycle associated 8	2.64
1434767_at	Mis18bp1	MIS18 binding protein 1	2.87
1437580_s_at	Nek2	NIMA (never in mitosis gene a)-related expressed kinase 2	2.25
1452315_at	Kif11	kinesin family member 11	2.76
1428105_at	Tpx2	TPX2, microtubule-associated protein homolog (*Xenopus laevis*)	3.67
1419943_s_at	Ccnb1	cyclin B1	5.38
1448205_at	Ccnb1	cyclin B1	4.28
1437251_at	Cdca2	cell division cycle associated 2	3.02
1439510_at	Sgol1	shugoshin-like 1 (*S. pombe*)	2.32
1415945_at	Mcm5	minichromosome maintenance deficient 5, cell division cycle 46 (*S. cerevisiae*)	2.40
1454694_a_at	Top2a	topoisomerase (DNA) II alpha	3.77
1439377_x_at	Cdc20	cell division cycle 20	3.53
1435005_at	Cenpe	centromere protein E	2.02
1417323_at	Psrc1	proline/serine-rich coiled-coil 1	2.58
1448314_at	Cdk1	cyclin-dependent kinase 1	3.61

1424046_at	Bub1	budding uninhibited by benzimidazoles 1 homolog (*S. cerevisiae*)	4.21
1418816_at	Chmp1b	charged multivesicular body protein 1B	1.69
1416961_at	Bub1b	budding uninhibited by benzimidazoles 1 homolog, beta (*S. cerevisiae*)	2.73
1447363_s_at	Bub1b	budding uninhibited by benzimidazoles 1 homolog, beta (*S. cerevisiae*)	4.77
**Cell cycle GO:7049 (N=33; P=1.52e-12)**			
1422851_at	Hmga2	high mobility group AT-hook 2	4.21
1450780_s_at	Hmga2	high mobility group AT-hook 2	4.07
1417082_at	Anp32b	acidic (leucine-rich) nuclear phosphoprotein 32 family, member B	2.03
1450920_at	Ccnb2	cyclin B2	5.05
1428481_s_at	Cdca8	cell division cycle associated 8	2.64
1434767_at	Mis18bp1	MIS18 binding protein 1	2.87
1416953_at	Ctgf	connective tissue growth factor	8.08
1452315_at	Kif11	kinesin family member 11	2.76
1438320_s_at	Mcm7	minichromosome maintenance deficient 7 (*S. cerevisiae*)	1.80
1456260_at	Rbbp4	retinoblastoma binding protein 4	-1.36
1423254_x_at	Rps27l	ribosomal protein S27-like	1.80
1435429_x_at	Rps27l	ribosomal protein S27-like	1.80
1450925_a_at	Rps27l	ribosomal protein S27-like	1.87
1439510_at	Sgol1	shugoshin-like 1 (*S. pombe*)	2.32
1460302_at	Thbs1	thrombospondin 1	6.33
1415945_at	Mcm5	minichromosome maintenance deficient 5, cell division cycle 46 (*S. cerevisiae*)	2.40
1439377_x_at	Cdc20	cell division cycle 20	3.53
1448314_at	Cdk1	cyclin-dependent kinase 1	3.61
1417323_at	Psrc1	proline/serine-rich coiled-coil 1	2.58
1418816_at	Chmp1b	charged multivesicular body protein 1B	1.69
1448213_at	Anxa1	annexin A1	6.10
1416961_at	Bub1b	budding uninhibited by benzimidazoles 1 homolog, beta (*S. cerevisiae*)	2.73

1447363_s_at	Bub1b	budding uninhibited by benzimidazoles 1 homolog, beta (*S. cerevisiae*)	4.77
1416309_at	Nusap1	nucleolar and spindle associated protein 1	5.09
1422441_x_at	Cdk4	cyclin-dependent kinase 4	1.75
1424107_at	Kif18a	kinesin family member 18A	1.50
1424105_a_at	Pttg1	pituitary tumor-transforming gene 1	2.86
1423775_s_at	Prc1	protein regulator of cytokinesis 1	3.17
1437580_s_at	Nek2	NIMA (never in mitosis gene a)-related expressed kinase 2	2.25
1419943_s_at	Ccnb1	cyclin B1	5.38
1448205_at	Ccnb1	cyclin B1	4.28
1428105_at	Tpx2	TPX2, microtubule-associated protein homolog (*Xenopus laevis*)	3.67
1448650_a_at	Pole	polymerase (DNA directed), epsilon	1.70
1437251_at	Cdca2	cell division cycle associated 2	3.02
1422430_at	Fignl1	fidgetin-like 1	2.08
1435005_at	Cenpe	centromere protein E	2.02
1424046_at	Bub1	budding uninhibited by benzimidazoles 1 homolog (*S. cerevisiae*)	4.21
1450842_a_at	Cenpa	centromere protein A	4.49
**Mitotic cell cycle GO:278 (N=24; P=1.65e-12)**			
1416309_at	Nusap1	nucleolar and spindle associated protein 1	5.09
1422851_at	Hmga2	high mobility group AT-hook 2	4.21
1450780_s_at	Hmga2	high mobility group AT-hook 2	4.07
1422441_x_at	Cdk4	cyclin-dependent kinase 4	1.75
1424107_at	Kif18a	kinesin family member 18A	1.50
1417082_at	Anp32b	acidic (leucine-rich) nuclear phosphoprotein 32 family, member B	2.03
1424105_a_at	Pttg1	pituitary tumor-transforming gene 1	2.86
1450920_at	Ccnb2	cyclin B2	5.05
1428481_s_at	Cdca8	cell division cycle associated 8	2.64
1434767_at	Mis18bp1	MIS18 binding protein 1	2.87

1437580_s_at	Nek2	NIMA (never in mitosis gene a)-related expressed kinase 2	2.25
1452315_at	Kif11	kinesin family member 11	2.76
1428105_at	Tpx2	TPX2, microtubule-associated protein homolog (Xenopus laevis)	3.67
1419943_s_at	Ccnb1	cyclin B1	5.38
1448205_at	Ccnb1	cyclin B1	4.28
1423254_x_at	Rps27l	ribosomal protein S27-like	1.80
1435429_x_at	Rps27l	ribosomal protein S27-like	1.80
1450925_a_at	Rps27l	ribosomal protein S27-like	1.87
1448650_a_at	Pole	polymerase (DNA directed), epsilon	1.70
1437251_at	Cdca2	cell division cycle associated 2	3.02
1439510_at	Sgol1	shugoshin-like 1 (*S. pombe*)	2.32
1439377_x_at	Cdc20	cell division cycle 20	3.53
1435005_at	Cenpe	centromere protein E	2.02
1417323_at	Psrc1	proline/serine-rich coiled-coil 1	2.58
1448314_at	Cdk1	cyclin-dependent kinase 1	3.61
1424046_at	Bub1	budding uninhibited by benzimidazoles 1 homolog (*S. cerevisiae*)	4.21
1450842_a_at	Cenpa	centromere protein A	4.49
1416961_at	Bub1b	budding uninhibited by benzimidazoles 1 homolog, beta (*S. cerevisiae*)	2.73
1447363_s_at	Bub1b	budding uninhibited by benzimidazoles 1 homolog, beta (*S. cerevisiae*)	4.77
**Nuclear division GO:280 (N=19; P=3.64e-12)**			
1416309_at	Nusap1	nucleolar and spindle associated protein 1	5.09
1422851_at	Hmga2	high mobility group AT-hook 2	4.21
1450780_s_at	Hmga2	high mobility group AT-hook 2	4.07
1424107_at	Kif18a	kinesin family member 18A	1.50
1424105_a_at	Pttg1	pituitary tumor-transforming gene 1	2.86
1450920_at	Ccnb2	cyclin B2	5.05
1428481_s_at	Cdca8	cell division cycle associated 8	2.64

1434767_at	Mis18bp1	MIS18 binding protein 1	2.87
1437580_s_at	Nek2	NIMA (never in mitosis gene a)-related expressed kinase 2	2.25
1452315_at	Kif11	kinesin family member 11	2.76
1419943_s_at	Ccnb1	cyclin B1	5.38
1448205_at	Ccnb1	cyclin B1	4.28
1428105_at	Tpx2	TPX2, microtubule-associated protein homolog (*Xenopus laevis*)	3.67
1437251_at	Cdca2	cell division cycle associated 2	3.02
1439510_at	Sgol1	shugoshin-like 1 (*S. pombe*)	3.53
1439377_x_at	Cdc20	cell division cycle 20	3.53
1435005_at	Cenpe	centromere protein E	2.02
1417323_at	Psrc1	proline/serine-rich coiled-coil 1	2.58
1448314_at	Cdk1	cyclin-dependent kinase 1	3.61
1424046_at	Bub1	budding uninhibited by benzimidazoles 1 homolog (*S. cerevisiae*)	4.21
1416961_at	Bub1b	budding uninhibited by benzimidazoles 1 homolog, beta (*S. cerevisiae*)	2.73
1447363_s_at	Bub1b	budding uninhibited by benzimidazoles 1 homolog, beta (*S. cerevisiae*)	4.77
**Mitosis GO:7067 (N=19; P=3.64e-12)**			
1416309_at	Nusap1	nucleolar and spindle associated protein 1	5.09
1422851_at	Hmga2	high mobility group AT-hook 2	4.21
1450780_s_at	Hmga2	high mobility group AT-hook 2	4.07
1424107_at	Kif18a	kinesin family member 18A	1.50
1424105_a_at	Pttg1	pituitary tumor-transforming gene 1	2.86
1450920_at	Ccnb2	cyclin B2	5.05
1428481_s_at	Cdca8	cell division cycle associated 8	2.64
1434767_at	Mis18bp1	MIS18 binding protein 1	2.87
1437580_s_at	Nek2	NIMA (never in mitosis gene a)-related expressed kinase 2	2.25
1452315_at	Kif11	kinesin family member 11	2.76
1419943_s_at	Ccnb1	cyclin B1	5.38

1448205_at	Ccnb1	cyclin B1	4.28
1428105_at	Tpx2	TPX2, microtubule-associated protein homolog (*Xenopus laevis*)	3.67
1437251_at	Cdca2	cell division cycle associated 2	3.02
1439510_at	Sgol1	shugoshin-like 1 (S. pombe)	2.32
1439377_x_at	Cdc20	cell division cycle 20	3.53
1435005_at	Cenpe	centromere protein E	2.02
1417323_at	Psrc1	proline/serine-rich coiled-coil 1	2.58
1448314_at	Cdk1	cyclin-dependent kinase 1	3.61
1424046_at	Bub1	budding uninhibited by benzimidazoles 1 homolog (*S. cerevisiae*)	4.21
1416961_at	Bub1b	budding uninhibited by benzimidazoles 1 homolog, beta (*S. cerevisiae*)	2.73
1447363_s_at	Bub1b	budding uninhibited by benzimidazoles 1 homolog, beta (*S. cerevisiae*)	4.77
**M phase of mitotic cell cycle GO:87 (N=19; P=4.37e-12)**			
1416309_at	Nusap1	nucleolar and spindle associated protein 1	5.09
1422851_at	Hmga2	high mobility group AT-hook 2	4.21
1450780_s_at	Hmga2	high mobility group AT-hook 2	4.07
1424107_at	Kif18a	kinesin family member 18A	1.50
1424105_a_at	Pttg1	pituitary tumor-transforming gene 1	2.86
1450920_at	Ccnb2	cyclin B2	5.05
1428481_s_at	Cdca8	cell division cycle associated 8	2.64
1434767_at	Mis18bp1	MIS18 binding protein 1	2.87
1437580_s_at	Nek2	NIMA (never in mitosis gene a)-related expressed kinase 2	2.25
1452315_at	Kif11	kinesin family member 11	2.76
1419943_s_at	Ccnb1	cyclin B1	5.38
1448205_at	Ccnb1	cyclin B1	4.28
1428105_at	Tpx2	TPX2, microtubule-associated protein homolog (*Xenopus laevis*)	3.67
1437251_at	Cdca2	cell division cycle associated 2	3.02
1439510_at	Sgol1	shugoshin-like 1 (*S. pombe*)	2.32

1439377_x_at	Cdc20	cell division cycle 20	3.53
1435005_at	Cenpe	centromere protein E	2.02
1417323_at	Psrc1	proline/serine-rich coiled-coil 1	2.58
1448314_at	Cdk1	cyclin-dependent kinase 1	3.61
1424046_at	Bub1	budding uninhibited by benzimidazoles 1 homolog (*S. cerevisiae*)	4.21
1416961_at	Bub1b	budding uninhibited by benzimidazoles 1 homolog, beta (*S. cerevisiae*)	2.73
1447363_s_at	Bub1b	budding uninhibited by benzimidazoles 1 homolog, beta (*S. cerevisiae*)	4.77
**Organelle fission GO:48285 (N=19; P=8.41e-12)**			
1416309_at	Nusap1	nucleolar and spindle associated protein 1	5.09
1422851_at	Hmga2	high mobility group AT-hook 2	4.21
1450780_s_at	Hmga2	high mobility group AT-hook 2	4.07
1424107_at	Kif18a	kinesin family member 18A	1.50
1424105_a_at	Pttg1	pituitary tumor-transforming gene 1	2.86
1450920_at	Ccnb2	cyclin B2	5.05
1428481_s_at	Cdca8	cell division cycle associated 8	2.64
1434767_at	Mis18bp1	MIS18 binding protein 1	2.87
1437580_s_at	Nek2	NIMA (never in mitosis gene a)-related expressed kinase 2	2.25
1452315_at	Kif11	kinesin family member 11	2.76
1419943_s_at	Ccnb1	cyclin B1	5.38
1448205_at	Ccnb1	cyclin B1	4.28
1428105_at	Tpx2	TPX2, microtubule-associated protein homolog (*Xenopus laevis*)	3.67
1437251_at	Cdca2	cell division cycle associated 2	3.02
1439510_at	Sgol1	shugoshin-like 1 (*S. pombe*)	2.32
1439377_x_at	Cdc20	cell division cycle 20	3.53
1435005_at	Cenpe	centromere protein E	2.02
1417323_at	Psrc1	proline/serine-rich coiled-coil 1	2.58
1448314_at	Cdk1	cyclin-dependent kinase 1	3.61

1424046_at	Bub1	budding uninhibited by benzimidazoles 1 homolog (*S. cerevisiae*)	4.21
1416961_at	Bub1b	budding uninhibited by benzimidazoles 1 homolog, beta (*S. cerevisiae*)	2.73
1447363_s_at	Bub1b	budding uninhibited by benzimidazoles 1 homolog, beta (*S. cerevisiae*)	4.77
**Cell cycle process GO:22402 (N=26; P=5.43e-11)**			
1416309_at	Nusap1	nucleolar and spindle associated protein 1	5.09
1422851_at	Hmga2	high mobility group AT-hook 2	4.21
1450780_s_at	Hmga2	high mobility group AT-hook 2	4.07
1422441_x_at	Cdk4	cyclin-dependent kinase 4	1.75
1424107_at	Kif18a	kinesin family member 18A	1.50
1417082_at	Anp32b	acidic (leucine-rich) nuclear phosphoprotein 32 family, member B	2.03
1424105_a_at	Pttg1	pituitary tumor-transforming gene 1	2.86
1450920_at	Ccnb2	cyclin B2	5.05
1428481_s_at	Cdca8	cell division cycle associated 8	2.64
1434767_at	Mis18bp1	MIS18 binding protein 1	2.87
1416953_at	Ctgf	connective tissue growth factor	8.08
1437580_s_at	Nek2	NIMA (never in mitosis gene a)-related expressed kinase 2	2.25
1452315_at	Kif11	kinesin family member 11	2.76
1428105_at	Tpx2	TPX2, microtubule-associated protein homolog (*Xenopus laevis*)	3.67
1419943_s_at	Ccnb1	cyclin B1	5.38
1448205_at	Ccnb1	cyclin B1	4.28
1423254_x_at	Rps27l	ribosomal protein S27-like	1.80
1435429_x_at	Rps27l	ribosomal protein S27-like	1.80
1450925_a_at	Rps27l	ribosomal protein S27-like	1.87
1448650_a_at	Pole	polymerase (DNA directed), epsilon	1.70
1437251_at	Cdca2	cell division cycle associated 2	3.02
1439510_at	Sgol1	shugoshin-like 1 (S. pombe)	2.32
1460302_at	Thbs1	thrombospondin 1	6.33

1439377_x_at	Cdc20	cell division cycle 20	3.53
1435005_at	Cenpe	centromere protein E	2.02
1448314_at	Cdk1	cyclin-dependent kinase 1	3.61
1417323_at	Psrc1	proline/serine-rich coiled-coil 1	2.58
1424046_at	Bub1	budding uninhibited by benzimidazoles 1 homolog (*S. cerevisiae*)	4.21
1450842_a_at	Cenpa	centromere protein A	4.49
1416961_at	Bub1b	budding uninhibited by benzimidazoles 1 homolog, beta (*S. cerevisiae*)	2.73
1447363_s_at	Bub1b	budding uninhibited by benzimidazoles 1 homolog, beta (*S. cerevisiae*)	4.77
**Cell cycle phase GO:22403 (N=23; P=8.04e-11)**			
1416309_at	Nusap1	nucleolar and spindle associated protein 1	5.09
1422851_at	Hmga2	high mobility group AT-hook 2	4.21
1450780_s_at	Hmga2	high mobility group AT-hook 2	4.07
1422441_x_at	Cdk4	cyclin-dependent kinase 4	1.75
1424107_at	Kif18a	kinesin family member 18A	1.50
1417082_at	Anp32b	acidic (leucine-rich) nuclear phosphoprotein 32 family, member B	2.03
1424105_a_at	Pttg1	pituitary tumor-transforming gene 1	2.86
1450920_at	Ccnb2	cyclin B2	5.05
1428481_s_at	Cdca8	cell division cycle associated 8	2.64
1434767_at	Mis18bp1	MIS18 binding protein 1	2.87
1437580_s_at	Nek2	NIMA (never in mitosis gene a)-related expressed kinase 2	2.25
1452315_at	Kif11	kinesin family member 11	2.76
1428105_at	Tpx2	TPX2, microtubule-associated protein homolog (*Xenopus laevis*)	3.67
1419943_s_at	Ccnb1	cyclin B1	5.38
1448205_at	Ccnb1	cyclin B1	4.28
1423254_x_at	Rps27l	ribosomal protein S27-like	1.80
1435429_x_at	Rps27l	ribosomal protein S27-like	1.80
1450925_a_at	Rps27l	ribosomal protein S27-like	1.87

1448650_a_at	Pole	polymerase (DNA directed), epsilon	1.70
1437251_at	Cdca2	cell division cycle associated 2	3.02
1439510_at	Sgol1	shugoshin-like 1 (*S. pombe*)	2.32
1439377_x_at	Cdc20	cell division cycle 20	3.53
1435005_at	Cenpe	centromere protein E	2.02
1417323_at	Psrc1	proline/serine-rich coiled-coil 1	2.58
1448314_at	Cdk1	cyclin-dependent kinase 1	3.61
1424046_at	Bub1	budding uninhibited by benzimidazoles 1 homolog (*S. cerevisiae*)	4.21
1416961_at	Bub1b	budding uninhibited by benzimidazoles 1 homolog, beta (*S. cerevisiae*)	2.73
1447363_s_at	Bub1b	budding uninhibited by benzimidazoles 1 homolog, beta (*S. cerevisiae*)	4.77
**M phase GO:279 (N=19; P=1.36e-09)**			
1416309_at	Nusap1	nucleolar and spindle associated protein 1	5.09
1422851_at	Hmga2	high mobility group AT-hook 2	4.21
1450780_s_at	Hmga2	high mobility group AT-hook 2	4.07
1424107_at	Kif18a	kinesin family member 18A	1.50
1424105_a_at	Pttg1	pituitary tumor-transforming gene 1	2.86
1450920_at	Ccnb2	cyclin B2	5.05
1428481_s_at	Cdca8	cell division cycle associated 8	2.64
1434767_at	Mis18bp1	MIS18 binding protein 1	2.87
1437580_s_at	Nek2	NIMA (never in mitosis gene a)-related expressed kinase 2	2.25
1452315_at	Kif11	kinesin family member 11	2.76
1419943_s_at	Ccnb1	cyclin B1	5.38
1448205_at	Ccnb1	cyclin B1	4.28
1428105_at	Tpx2	TPX2, microtubule-associated protein homolog (*Xenopus laevis*)	3.67
1437251_at	Cdca2	cell division cycle associated 2	3.02
1439510_at	Sgol1	shugoshin-like 1 (*S. pombe*)	2.32
1439377_x_at	Cdc20	cell division cycle 20	3.53

1435005_at	Cenpe	centromere protein E	2.02
1417323_at	Psrc1	proline/serine-rich coiled-coil 1	2.58
1448314_at	Cdk1	cyclin-dependent kinase 1	3.61
1424046_at	Bub1	budding uninhibited by benzimidazoles 1 homolog (*S. cerevisiae*)	4.21
1416961_at	Bub1b	budding uninhibited by benzimidazoles 1 homolog, beta (*S. cerevisiae*)	2.73
1447363_s_at	Bub1b	budding uninhibited by benzimidazoles 1 homolog, beta (*S. cerevisiae*)	4.77

MOLECULAR FUNCTION

**Protein binding GO:5515 (N=62; P=4.16e-06)**			
1417065_at	Egr1	early growth response 1	3.98
1458054_at	Ext1	exostoses (multiple) 1	-2.05
1422851_at	Hmga2	high mobility group AT-hook 2	4.21
1450780_s_at	Hmga2	high mobility group AT-hook 2	4.07
1425567_a_at	Anxa5	annexin A5	2.19
1422507_at	Cstb	cystatin B	-3.62
1422506_a_at	Cstb	cystatin B	-4.23
1423607_at	Lum	lumican	3.84
1456733_x_at	Serpinh1	serine (or cysteine) peptidase inhibitor, clade H, member 1	2.59
1450920_at	Ccnb2	cyclin B2	5.05
1425811_a_at	Csrp1	cysteine and glycine-rich protein 1	2.42
1417583_a_at	Utp3	UTP3, small subunit (SSU) processome component, homolog (*S. cerevisiae*)	1.46
1448228_at	Lox	lysyl oxidase	5.68
1428481_s_at	Cdca8	cell division cycle associated 8	2.64
1435695_a_at	Ggct	gamma-glutamyl cyclotransferase	2.07
1434767_at	Mis18bp1	MIS18 binding protein 1	2.87
1416953_at	Ctgf	connective tissue growth factor	8.08
1452315_at	Kif11	kinesin family member 11	2.76

1438650_x_at	Gja1	gap junction protein, alpha 1	1.88
1438320_s_at	Mcm7	minichromosome maintenance deficient 7 (*S. cerevisiae*)	1.80
1456260_at	Rbbp4	retinoblastoma binding protein 4	-1.36
1448259_at	Fstl1	follistatin-like 1	3.65
1450981_at	Cnn2	calponin 2	3.74
1419091_a_at	Anxa2	annexin A2	2.50
1448594_at	Wisp1	WNT1 inducible signaling pathway protein 1	1.85
1454694_a_at	Top2a	topoisomerase (DNA) II alpha	3.77
1460302_at	Thbs1	thrombospondin 1	6.33
1439377_x_at	Cdc20	cell division cycle 20	3.53
1448314_at	Cdk1	cyclin-dependent kinase 1	3.61
1417323_at	Psrc1	proline/serine-rich coiled-coil 1	2.58
1418816_at	Chmp1b	charged multivesicular body protein 1B	1.69
1416440_at	Cd164	CD164 antigen	1.81
1448213_at	Anxa1	annexin A1	6.10
1416961_at	Bub1b	budding uninhibited by benzimidazoles 1 homolog, beta (*S. cerevisiae*)	2.73
1447363_s_at	Bub1b	budding uninhibited by benzimidazoles 1 homolog, beta (*S. cerevisiae*)	4.77
1452968_at	Cthrc1	collagen triple helix repeat containing 1	4.74
1425028_a_at	Tpm2	tropomyosin 2, beta	3.48
1428585_at	Actn1	actinin, alpha 1	2.13
1437165_a_at	Pcolce	procollagen C-endopeptidase enhancer protein	4.80
1448433_a_at	Pcolce	procollagen C-endopeptidase enhancer protein	2.19
1416309_at	Nusap1	nucleolar and spindle associated protein 1	5.09
1422441_x_at	Cdk4	cyclin-dependent kinase 4	1.75
1424107_at	Kif18a	kinesin family member 18A	1.50
1424105_a_at	Pttg1	pituitary tumor-transforming gene 1	2.86
1448103_s_at	Nono	non-POU-domain-containing, octamer binding protein	1.36

1423775_s_at	Prc1	protein regulator of cytokinesis 1	3.17
1448238_at	2700060E02Rik	RIKEN cDNA 2700060E02 gene	1.37
1452598_at	Gins1	GINS complex subunit 1 (Psf1 homolog)	2.16
1451194_at	Aldob	aldolase B, fructose-bisphosphate	-1.36
1425964_x_at	Hspb1	heat shock protein 1	3.11
1425528_at	Prrx1	paired related homeobox 1	1.96
1437580_s_at	Nek2	NIMA (never in mitosis gene a)-related expressed kinase 2	2.25
1423516_a_at	Nid2	nidogen 2	2.51
1428105_at	Tpx2	TPX2, microtubule-associated protein homolog (*Xenopus laevis*)	3.67
1419943_s_at	Ccnb1	cyclin B1	5.38
1448205_at	Ccnb1	cyclin B1	4.28
1423110_at	Col1a2	collagen, type I, alpha 2	10.63
1450857_a_at	Col1a2	collagen, type I, alpha 2	6.37
1438115_a_at	Slc9a3r1	solute carrier family 9 (sodium/hydrogen exchanger), member 3 regulator 1	2.12
1416064_a_at	Hspa5	heat shock protein 5	1.73
1416039_x_at	Cyr61	cysteine rich protein 61	3.72
1457823_at	Cyr61	cysteine rich protein 61	2.20
1435005_at	Cenpe	centromere protein E	2.02
1427883_a_at	Col3a1	collagen, type III, alpha 1	11.26
1419149_at	Serpine1	serine (or cysteine) peptidase inhibitor, clade E, member 1	4.39
1417394_at	Klf4	Kruppel-like factor 4 (gut)	2.37
1416416_x_at	Gstm1	glutathione S-transferase, mu 1	1.47
1450842_a_at	Cenpa	centromere protein A	4.49
1420478_at	Nap1l1	nucleosome assembly protein 1-like 1	1.59
**Binding GO:5488 (N=83; P=2.33e-05)**			
1456733_x_at	Serpinh1	serine (or cysteine) peptidase inhibitor, clade H, member 1	2.59
1425811_a_at	Csrp1	cysteine and glycine-rich protein 1	2.42

1454862_at	Phldb2	pleckstrin homology-like domain, family B, member 2	3.53
1428481_s_at	Cdca8	cell division cycle associated 8	2.64
1434767_at	Mis18bp1	MIS18 binding protein 1	2.87
1438650_x_at	Gja1	gap junction protein, alpha 1	1.88
1423254_x_at	Rps27l	ribosomal protein S27-like	1.80
1435429_x_at	Rps27l	ribosomal protein S27-like	1.80
1450925_a_at	Rps27l	ribosomal protein S27-like	1.87
1450981_at	Cnn2	calponin 2	3.74
1460302_at	Thbs1	thrombospondin 1	6.33
1415945_at	Mcm5	minichromosome maintenance deficient 5, cell division cycle 46 (*S. cerevisiae*)	2.40
1439377_x_at	Cdc20	cell division cycle 20	3.53
1417323_at	Psrc1	proline/serine-rich coiled-coil 1	2.58
1437626_at	Zfp36l2	zinc finger protein 36, C3H type-like 2	2.38
1418816_at	Chmp1b	charged multivesicular body protein 1B	1.69
1416440_at	Cd164	CD164 antigen	1.81
1448213_at	Anxa1	annexin A1	6.10
1416961_at	Bub1b	budding uninhibited by benzimidazoles 1 homolog, beta (*S. cerevisiae*)	2.73
1447363_s_at	Bub1b	budding uninhibited by benzimidazoles 1 homolog, beta (*S. cerevisiae*)	4.77
1452968_at	Cthrc1	collagen triple helix repeat containing 1	4.74
1425028_a_at	Tpm2	tropomyosin 2, beta	3.48
1428585_at	Actn1	actinin, alpha 1	2.13
1422441_x_at	Cdk4	cyclin-dependent kinase 4	1.75
1448103_s_at	Nono	non-POU-domain-containing, octamer binding protein	1.36
1448238_at	2700060E02Rik	RIKEN cDNA 2700060E02 gene	1.37
1451194_at	Aldob	aldolase B, fructose-bisphosphate	-1.36
1434173_s_at	D19Bwg1357e	DNA segment, Chr 19, Brigham & Women's Genetics 1357 expressed	1.33
1425964_x_at	Hspb1	heat shock protein 1	3.11

1425528_at	Prrx1	paired related homeobox 1	1.96
1437580_s_at	Nek2	NIMA (never in mitosis gene a)-related expressed kinase 2	2.25
1450156_a_at	Hmmr	hyaluronan mediated motility receptor (RHAMM)	2.46
1436155_at	Nmnat2	nicotinamide nucleotide adenylyltransferase 2	-1.62
1456444_at	Fbxo41	F-box protein 41	-1.41
1416064_a_at	Hspa5	heat shock protein 5	1.73
1435005_at	Cenpe	centromere protein E	2.02
1415754_at	Polr2f	polymerase (RNA) II (DNA directed) polypeptide F	1.34
1448627_s_at	Pbk	PDZ binding kinase	2.43
1416416_x_at	Gstm1	glutathione S-transferase, mu 1	1.47
1417394_at	Klf4	Kruppel-like factor 4 (gut)	2.37
1420478_at	Nap1l1	nucleosome assembly protein 1-like 1	1.59
1417065_at	Egr1	early growth response 1	3.98
1417777_at	Ptgr1	prostaglandin reductase 1	3.98
1422851_at	Hmga2	high mobility group AT-hook 2	4.21
1450780_s_at	Hmga2	high mobility group AT-hook 2	4.07
1458054_at	Ext1	exostoses (multiple) 1	-2.05
1425567_a_at	Anxa5	annexin A5	2.19
1422507_at	Cstb	cystatin B	-3.62
1422506_a_at	Cstb	cystatin B	-4.23
1423607_at	Lum	lumican	3.84
1450920_at	Ccnb2	cyclin B2	5.05
1448228_at	Lox	lysyl oxidase	5.68
1417583_a_at	Utp3	UTP3, small subunit (SSU) processome component, homolog (*S. cerevisiae*)	1.46
1421115_a_at	Zdhhc16	zinc finger, DHHC domain containing 16	1.36
1435695_a_at	Ggct	gamma-glutamyl cyclotransferase	2.07
1416953_at	Ctgf	connective tissue growth factor	8.08

1452315_at	Kif11	kinesin family member 11	2.76
1448259_at	Fstl1	follistatin-like 1	3.65
1456260_at	Rbbp4	retinoblastoma binding protein 4	-1.36
1438320_s_at	Mcm7	minichromosome maintenance deficient 7 (*S. cerevisiae*)	1.80
1454694_a_at	Top2a	topoisomerase (DNA) II alpha	3.77
1448594_at	Wisp1	WNT1 inducible signaling pathway protein 1	1.85
1419091_a_at	Anxa2	annexin A2	2.50
1416454_s_at	Acta2	actin, alpha 2, smooth muscle, aorta	4.75
1448314_at	Cdk1	cyclin-dependent kinase 1	3.61
1449335_at	Timp3	tissue inhibitor of metalloproteinase 3	3.85
1419088_at	Timp3	tissue inhibitor of metalloproteinase 3	3.80
1419089_at	Timp3	tissue inhibitor of metalloproteinase 3	2.73
1423813_at	Kif22	kinesin family member 22	2.13
1416309_at	Nusap1	nucleolar and spindle associated protein 1	5.09
1437165_a_at	Pcolce	procollagen C-endopeptidase enhancer protein	4.80
1448433_a_at	Pcolce	procollagen C-endopeptidase enhancer protein	2.19
1424107_at	Kif18a	kinesin family member 18A	1.50
1424105_a_at	Pttg1	pituitary tumor-transforming gene 1	2.86
1423775_s_at	Prc1	protein regulator of cytokinesis 1	3.17
1416431_at	Tubb6	tubulin, beta 6 class V	2.24
1452598_at	Gins1	GINS complex subunit 1 (Psf1 homolog)	2.16
1423516_a_at	Nid2	nidogen 2	2.51
1449181_at	Fech	Ferrochelatase	1.37
1428105_at	Tpx2	TPX2, microtubule-associated protein homolog (*Xenopus laevis*)	3.67
1419943_s_at	Ccnb1	cyclin B1	5.38
1448205_at	Ccnb1	cyclin B1	4.28
1423110_at	Col1a2	collagen, type I, alpha 2	10.63

1450857_a_at	Col1a2	collagen, type I, alpha 2	6.37
1448650_a_at	Pole	polymerase (DNA directed), epsilon	1.70
1438115_a_at	Slc9a3r1	solute carrier family 9 (sodium/hydrogen exchanger), member 3 regulator 1	2.12
1422430_at	Fignl1	fidgetin-like 1	2.08
1437889_x_at	Bgn	biglycan	1.93
1416405_at	Bgn	biglycan	3.00
1416039_x_at	Cyr61	cysteine rich protein 61	3.72
1457823_at	Cyr61	cysteine rich protein 61	2.20
1427883_a_at	Col3a1	collagen, type III, alpha 1	11.26
1424046_at	Bub1	budding uninhibited by benzimidazoles 1 homolog (*S. cerevisiae*)	4.21
1419149_at	Serpine1	serine (or cysteine) peptidase inhibitor, clade E, member 1	4.39
1450842_a_at	Cenpa	centromere protein A	4.49
**Enzyme binding GO:19899 (N=20; P=0.0003)**			
1417065_at	Egr1	early growth response 1	3.98
1422851_at	Hmga2	high mobility group AT-hook 2	4.21
1450780_s_at	Hmga2	high mobility group AT-hook 2	4.07
1422507_at	Cstb	cystatin B	-3.62
1422506_a_at	Cstb	cystatin B	-4.23
1423775_s_at	Prc1	protein regulator of cytokinesis 1	3.17
1448238_at	2700060E02Rik	RIKEN cDNA 2700060E02 gene	1.37
1451194_at	Aldob	aldolase B, fructose-bisphosphate	-1.36
1450920_at	Ccnb2	cyclin B2	5.05
1425964_x_at	Hspb1	heat shock protein 1	3.11
1437580_s_at	Nek2	NIMA (never in mitosis gene a)-related expressed kinase 2	2.25
1452315_at	Kif11	kinesin family member 11	2.76
1428105_at	Tpx2	TPX2, microtubule-associated protein homolog (*Xenopus laevis*)	3.67
1419943_s_at	Ccnb1	cyclin B1	5.38

1448205_at	Ccnb1	cyclin B1	4.28
1456260_at	Rbbp4	retinoblastoma binding protein 4	-1.36
1438115_a_at	Slc9a3r1	solute carrier family 9 (sodium/hydrogen exchanger), member 3 regulator 1	2.12
1416064_a_at	Hspa5	heat shock protein 5	1.73
1454694_a_at	Top2a	topoisomerase (DNA) II alpha	3.77
1419091_a_at	Anxa2	annexin A2	2.50
1439377_x_at	Cdc20	cell division cycle 20	3.53
1435005_at	Cenpe	centromere protein E	2.02
1419149_at	Serpine1	serine (or cysteine) peptidase inhibitor, clade E, member 1	4.39
**Peptidase regulator activity GO:61134 (N=8; P=0.0003)**			
1423254_x_at	Rps27l	ribosomal protein S27-like	1.80
1435429_x_at	Rps27l	ribosomal protein S27-like	1.80
1450925_a_at	Rps27l	ribosomal protein S27-like	1.87
1437165_a_at	Pcolce	procollagen C-endopeptidase enhancer protein	4.80
1448433_a_at	Pcolce	procollagen C-endopeptidase enhancer protein	2.19
1416064_a_at	Hspa5	heat shock protein 5	1.73
1422507_at	Cstb	cystatin B	-3.62
1422506_a_at	Cstb	cystatin B	-4.23
1424105_a_at	Pttg1	pituitary tumor-transforming gene 1	2.86
1456733_x_at	Serpinh1	serine (or cysteine) peptidase inhibitor, clade H, member 1	2.59
1449335_at	Timp3	tissue inhibitor of metalloproteinase 3	3.85
1419088_at	Timp3	tissue inhibitor of metalloproteinase 3	3.80
1419089_at	Timp3	tissue inhibitor of metalloproteinase 3	2.73
1419149_at	Serpine1	serine (or cysteine) peptidase inhibitor, clade E, member 1	4.39
**Enzyme inhibitor activity GO:4857(N=9; P=0.0005)**			
1416064_a_at	Hspa5	heat shock protein 5	1.73
1419091_a_at	Anxa2	annexin A2	2.50

1422507_at	Cstb	cystatin B	-3.62
1422506_a_at	Cstb	cystatin B	-4.23
1424105_a_at	Pttg1	pituitary tumor-transforming gene 1	2.86
1456733_x_at	Serpinh1	serine (or cysteine) peptidase inhibitor, clade H, member 1	2.59
1419149_at	Serpine1	serine (or cysteine) peptidase inhibitor, clade E, member 1	4.39
1449335_at	Timp3	tissue inhibitor of metalloproteinase 3	3.85
1419088_at	Timp3	tissue inhibitor of metalloproteinase 3	3.80
1419089_at	Timp3	tissue inhibitor of metalloproteinase 3	2.73
1425964_x_at	Hspb1	heat shock protein 1	3.11
1448213_at	Anxa1	annexin A1	6.10
**Glycosaminoglycan binding GO:5539 (N=7; P=0.0005)**			
1448259_at	Fstl1	follistatin-like 1	3.65
1437165_a_at	Pcolce	procollagen C-endopeptidase enhancer protein	4.80
1448433_a_at	Pcolce	procollagen C-endopeptidase enhancer protein	2.19
1437889_x_at	Bgn	biglycan	1.93
1416405_at	Bgn	Biglycan	3.00
1416953_at	Ctgf	connective tissue growth factor	8.08
1460302_at	Thbs1	thrombospondin 1	6.33
1416039_x_at	Cyr61	cysteine rich protein 61	3.72
1457823_at	Cyr61	cysteine rich protein 61	2.20
1450156_a_at	Hmmr	hyaluronan mediated motility receptor (RHAMM)	2.46
**Growth factor binding GO:19838 (N=6; P=0.0007)**			
1423110_at	Col1a2	collagen, type I, alpha 2	10.63
1450857_a_at	Col1a2	collagen, type I, alpha 2	6.37
1448594_at	Wisp1	WNT1 inducible signaling pathway protein 1	1.85
1416953_at	Ctgf	connective tissue growth factor	8.08
1460302_at	Thbs1	thrombospondin 1	6.33

1416039_x_at	Cyr61	cysteine rich protein 61	3.72
1457823_at	Cyr61	cysteine rich protein 61	2.20
1427883_a_at	Col3a1	collagen, type III, alpha 1	11.26
**Carbohydrate derivative binding GO:97367 (N=7; P=0.0008)**			
1448259_at	Fstl1	follistatin-like 1	3.65
1437165_a_at	Pcolce	procollagen C-endopeptidase enhancer protein	4.80
1448433_a_at	Pcolce	procollagen C-endopeptidase enhancer protein	2.19
1437889_x_at	Bgn	biglycan	1.93
1416405_at	Bgn	Biglycan	3.00
1416953_at	Ctgf	connective tissue growth factor	8.08
1460302_at	Thbs1	thrombospondin 1	6.33
1416039_x_at	Cyr61	cysteine rich protein 61	3.72
1457823_at	Cyr61	cysteine rich protein 61	2.20
1450156_a_at	Hmmr	hyaluronan mediated motility receptor (RHAMM)	2.46
**Integrin binding GO:5178 (N=5; P=0.0008)**			
1428585_at	Actn1	actinin, alpha 1	2.13
1416953_at	Ctgf	connective tissue growth factor	8.08
1460302_at	Thbs1	thrombospondin 1	6.33
1416039_x_at	Cyr61	cysteine rich protein 61	3.72
1457823_at	Cyr61	cysteine rich protein 61	2.20
1427883_a_at	Col3a1	collagen, type III, alpha 1	11.26
**Endopeptidase inhibitor activity GO:4866 (N=6; P=0.0014)**			
1456733_x_at	Serpinh1	serine (or cysteine) peptidase inhibitor, clade H, member 1	2.59
1419149_at	Serpine1	serine (or cysteine) peptidase inhibitor, clade E, member 1	4.39
1449335_at	Timp3	tissue inhibitor of metalloproteinase 3	3.85
1419088_at	Timp3	tissue inhibitor of metalloproteinase 3	3.80
1419089_at	Timp3	tissue inhibitor of metalloproteinase 3	2.73

1416064_a_at	Hspa5	heat shock protein 5	1.73
1422507_at	Cstb	cystatin B	-3.62
1422506_a_at	Cstb	cystatin B	-4.23
1424105_a_at	Pttg1	pituitary tumor-transforming gene 1	2.86

CELLULAR COMPONENT

**Intracellular non-membrane-bounded organelle GO:43232 (N=45; P=5.62e-09)**			
1422851_at	Hmga2	high mobility group AT-hook 2	4.21
1450780_s_at	Hmga2	high mobility group AT-hook 2	4.07
1422507_at	Cstb	cystatin B	-3.62
1422506_a_at	Cstb	cystatin B	-4.23
1450920_at	Ccnb2	cyclin B2	5.05
1425811_a_at	Csrp1	cysteine and glycine-rich protein 1	2.42
1417583_a_at	Utp3	UTP3, small subunit (SSU) processome component, homolog (*S. cerevisiae*)	1.46
1454862_at	Phldb2	pleckstrin homology-like domain, family B, member 2	3.53
1428481_s_at	Cdca8	cell division cycle associated 8	2.64
1434767_at	Mis18bp1	MIS18 binding protein 1	2.87
1416719_a_at	Rps10	ribosomal protein S10	1.48
1438723_a_at	Rps10	ribosomal protein S10	1.49
1452315_at	Kif11	kinesin family member 11	2.76
1438650_x_at	Gja1	gap junction protein, alpha 1	1.88
1423254_x_at	Rps27l	ribosomal protein S27-like	1.80
1435429_x_at	Rps27l	ribosomal protein S27-like	1.80
1450925_a_at	Rps27l	ribosomal protein S27-like	1.87
1450981_at	Cnn2	calponin 2	3.74
1439510_at	Sgol1	shugoshin-like 1 (*S. pombe*)	2.32
1419091_a_at	Anxa2	annexin A2	2.50

1454694_a_at	Top2a	topoisomerase (DNA) II alpha	3.77
1416454_s_at	Acta2	actin, alpha 2, smooth muscle, aorta	4.75
1439377_x_at	Cdc20	cell division cycle 20	3.53
1448314_at	Cdk1	cyclin-dependent kinase 1	3.61
1417323_at	Psrc1	proline/serine-rich coiled-coil 1	2.58
1423813_at	Kif22	kinesin family member 22	2.13
1448213_at	Anxa1	annexin A1	6.10
1416961_at	Bub1b	budding uninhibited by benzimidazoles 1 homolog, beta (*S. cerevisiae*)	2.73
1447363_s_at	Bub1b	budding uninhibited by benzimidazoles 1 homolog, beta (*S. cerevisiae*)	4.77
1425028_a_at	Tpm2	tropomyosin 2, beta	3.48
1428585_at	Actn1	actinin, alpha 1	2.13
1416309_at	Nusap1	nucleolar and spindle associated protein 1	5.09
1422441_x_at	Cdk4	cyclin-dependent kinase 4	1.75
1424107_at	Kif18a	kinesin family member 18A	1.50
1423775_s_at	Prc1	protein regulator of cytokinesis 1	3.17
1448238_at	2700060E02Rik	RIKEN cDNA 2700060E02 gene	1.37
1416431_at	Tubb6	tubulin, beta 6 class V	2.24
1451194_at	Aldob	aldolase B, fructose-bisphosphate	-1.36
1434173_s_at	D19Bwg1357e	DNA segment, Chr 19, Brigham & Women's Genetics 1357 expressed	1.33
1425964_x_at	Hspb1	heat shock protein 1	3.11
1425528_at	Prrx1	paired related homeobox 1	1.96
1437580_s_at	Nek2	NIMA (never in mitosis gene a)-related expressed kinase 2	2.25
1428105_at	Tpx2	TPX2, microtubule-associated protein homolog (*Xenopus laevis*)	3.67
1419943_s_at	Ccnb1	cyclin B1	5.38
1448205_at	Ccnb1	cyclin B1	4.28
1448650_a_at	Pole	polymerase (DNA directed), epsilon	1.70
1438115_a_at	Slc9a3r1	solute carrier family 9 (sodium/hydrogen exchanger), member 3 regulator 1	2.12

1435005_at	Cenpe	centromere protein E	2.02
1415754_at	Polr2f	polymerase (RNA) II (DNA directed) polypeptide F	1.34
1424046_at	Bub1	budding uninhibited by benzimidazoles 1 homolog (*S. cerevisiae*)	4.21
1417394_at	Klf4	Kruppel-like factor 4 (gut)	2.37
1450842_a_at	Cenpa	centromere protein A	4.49
**Non-membrane-bounded organelle GO:43228 (N=45; P=5.62e-09)**			
1422851_at	Hmga2	high mobility group AT-hook 2	4.21
1450780_s_at	Hmga2	high mobility group AT-hook 2	4.07
1422507_at	Cstb	cystatin B	-3.62
1422506_a_at	Cstb	cystatin B	-4.23
1450920_at	Ccnb2	cyclin B2	5.05
1425811_a_at	Csrp1	cysteine and glycine-rich protein 1	2.42
1417583_a_at	Utp3	UTP3, small subunit (SSU) processome component, homolog (*S. cerevisiae*)	1.46
1454862_at	Phldb2	pleckstrin homology-like domain, family B, member 2	3.53
1428481_s_at	Cdca8	cell division cycle associated 8	2.64
1434767_at	Mis18bp1	MIS18 binding protein 1	2.87
1416719_a_at	Rps10	ribosomal protein S10	1.48
1438723_a_at	Rps10	ribosomal protein S10	1.49
1452315_at	Kif11	kinesin family member 11	2.76
1438650_x_at	Gja1	gap junction protein, alpha 1	1.88
1423254_x_at	Rps27l	ribosomal protein S27-like	1.80
1435429_x_at	Rps27l	ribosomal protein S27-like	1.80
1450925_a_at	Rps27l	ribosomal protein S27-like	1.87
1450981_at	Cnn2	calponin 2	3.74
1439510_at	Sgol1	shugoshin-like 1 (*S. pombe*)	2.32
1419091_a_at	Anxa2	annexin A2	2.50
1454694_a_at	Top2a	topoisomerase (DNA) II alpha	3.77

1416454_s_at	Acta2	actin, alpha 2, smooth muscle, aorta	4.75
1439377_x_at	Cdc20	cell division cycle 20	3.53
1448314_at	Cdk1	cyclin-dependent kinase 1	3.61
1417323_at	Psrc1	proline/serine-rich coiled-coil 1	2.58
1423813_at	Kif22	kinesin family member 22	2.13
1448213_at	Anxa1	annexin A1	6.10
1416961_at	Bub1b	budding uninhibited by benzimidazoles 1 homolog, beta (*S. cerevisiae*)	2.73
1447363_s_at	Bub1b	budding uninhibited by benzimidazoles 1 homolog, beta (*S. cerevisiae*)	4.77
1425028_a_at	Tpm2	tropomyosin 2, beta	3.48
1428585_at	Actn1	actinin, alpha 1	2.13
1416309_at	Nusap1	nucleolar and spindle associated protein 1	5.09
1422441_x_at	Cdk4	cyclin-dependent kinase 4	1.75
1424107_at	Kif18a	kinesin family member 18A	1.50
1423775_s_at	Prc1	protein regulator of cytokinesis 1	3.17
1448238_at	2700060E02Rik	RIKEN cDNA 2700060E02 gene	1.37
1416431_at	Tubb6	tubulin, beta 6 class V	2.24
1451194_at	Aldob	aldolase B, fructose-bisphosphate	-1.36
1434173_s_at	D19Bwg1357e	DNA segment, Chr 19, Brigham & Women's Genetics 1357 expressed	1.33
1425964_x_at	Hspb1	heat shock protein 1	3.11
1425528_at	Prrx1	paired related homeobox 1	1.96
1437580_s_at	Nek2	NIMA (never in mitosis gene a)-related expressed kinase 2	2.25
1428105_at	Tpx2	TPX2, microtubule-associated protein homolog (*Xenopus laevis*)	3.67
1419943_s_at	Ccnb1	cyclin B1	5.38
1448205_at	Ccnb1	cyclin B1	4.28
1448650_a_at	Pole	polymerase (DNA directed), epsilon	1.70
1438115_a_at	Slc9a3r1	solute carrier family 9 (sodium/hydrogen exchanger), member 3 regulator 1	2.12
1435005_at	Cenpe	centromere protein E	2.02

1415754_at	Polr2f	polymerase (RNA) II (DNA directed) polypeptide F	1.34
1424046_at	Bub1	budding uninhibited by benzimidazoles 1 homolog (*S. cerevisiae*)	4.21
1417394_at	Klf4	Kruppel-like factor 4 (gut)	2.37
1450842_a_at	Cenpa	centromere protein A	4.49
**Spindle GO:5819 (N=12; P=8.37e-09)**			
1428105_at	Tpx2	TPX2, microtubule-associated protein homolog (*Xenopus laevis*)	3.67
1419943_s_at	Ccnb1	cyclin B1	5.38
1448205_at	Ccnb1	cyclin B1	4.28
1416309_at	Nusap1	nucleolar and spindle associated protein 1	5.09
1439510_at	Sgol1	shugoshin-like 1 (*S. pombe*)	2.32
1424107_at	Kif18a	kinesin family member 18A	1.50
1423775_s_at	Prc1	protein regulator of cytokinesis 1	3.17
1448314_at	Cdk1	cyclin-dependent kinase 1	3.61
1417323_at	Psrc1	proline/serine-rich coiled-coil 1	2.58
1428481_s_at	Cdca8	cell division cycle associated 8	2.64
1437580_s_at	Nek2	NIMA (never in mitosis gene a)-related expressed kinase 2	2.25
1423813_at	Kif22	kinesin family member 22	2.13
1452315_at	Kif11	kinesin family member 11	2.76
**Condensed nuclear chromosome, centromeric region GO:780 (N=6; P=9.52e-09)**			
1424046_at	Bub1	budding uninhibited by benzimidazoles 1 homolog (*S. cerevisiae*)	4.21
1419943_s_at	Ccnb1	cyclin B1	5.38
1448205_at	Ccnb1	cyclin B1	4.28
1450842_a_at	Cenpa	centromere protein A	4.49
1439510_at	Sgol1	shugoshin-like 1 (*S. pombe*)	2.32
1435005_at	Cenpe	centromere protein E	2.02
1416961_at	Bub1b	budding uninhibited by benzimidazoles 1 homolog, beta (*S. cerevisiae*)	2.73
1447363_s_at	Bub1b	budding uninhibited by benzimidazoles 1 homolog, beta (*S. cerevisiae*)	4.77

**Extracellular matrix GO:31012 (N=15; P=2.22e-07)**			
1452968_at	Cthrc1	collagen triple helix repeat containing 1	4.74
1423110_at	Col1a2	collagen, type I, alpha 2	10.63
1450857_a_at	Col1a2	collagen, type I, alpha 2	6.37
1437165_a_at	Pcolce	procollagen C-endopeptidase enhancer protein	4.80
1448433_a_at	Pcolce	procollagen C-endopeptidase enhancer protein	2.19
1437889_x_at	Bgn	biglycan	1.93
1416405_at	Bgn	Biglycan	3.00
1419091_a_at	Anxa2	annexin A2	2.50
1427883_a_at	Col3a1	collagen, type III, alpha 1	11.26
1423607_at	Lum	lumican	3.84
1448592_at	Crtap	cartilage associated protein	1.88
1419149_at	Serpine1	serine (or cysteine) peptidase inhibitor, clade E, member 1	4.39
1449335_at	Timp3	tissue inhibitor of metalloproteinase 3	3.85
1419088_at	Timp3	tissue inhibitor of metalloproteinase 3	3.80
1419089_at	Timp3	tissue inhibitor of metalloproteinase 3	2.73
1424131_at	Col6a3	collagen, type VI, alpha 3	3.79
1424051_at	Col4a2	collagen, type IV, alpha 2	3.41
1448228_at	Lox	lysyl oxidase	5.68
1416953_at	Ctgf	connective tissue growth factor	8.08
1423516_a_at	Nid2	nidogen 2	2.51
**Proteinaceous extracellular matrix GO:5578 (N=14; P=3.40e-07)**			
1452968_at	Cthrc1	collagen triple helix repeat containing 1	4.74
1423110_at	Col1a2	collagen, type I, alpha 2	10.63
1450857_a_at	Col1a2	collagen, type I, alpha 2	6.37
1437165_a_at	Pcolce	procollagen C-endopeptidase enhancer protein	4.80
1448433_a_at	Pcolce	procollagen C-endopeptidase enhancer protein	2.19

1437889_x_at	Bgn	biglycan	1.93
1416405_at	Bgn	biglycan	3.00
1419091_a_at	Anxa2	annexin A2	2.50
1427883_a_at	Col3a1	collagen, type III, alpha 1	11.26
1423607_at	Lum	lumican	3.84
1448592_at	Crtap	cartilage associated protein	1.88
1449335_at	Timp3	tissue inhibitor of metalloproteinase 3	3.85
1419088_at	Timp3	tissue inhibitor of metalloproteinase 3	3.80
1419089_at	Timp3	tissue inhibitor of metalloproteinase 3	2.73
1424131_at	Col6a3	collagen, type VI, alpha 3	3.79
1424051_at	Col4a2	collagen, type IV, alpha 2	3.41
1448228_at	Lox	lysyl oxidase	5.68
1416953_at	Ctgf	connective tissue growth factor	8.08
1423516_a_at	Nid2	nidogen 2	2.51
**Extracellular matrix part GO:44420 (N=11; P=3.71e-07)**			
1452968_at	Cthrc1	collagen triple helix repeat containing 1	4.74
1423110_at	Col1a2	collagen, type I, alpha 2	10.63
1450857_a_at	Col1a2	collagen, type I, alpha 2	6.37
1437165_a_at	Pcolce	procollagen C-endopeptidase enhancer protein	4.80
1448433_a_at	Pcolce	procollagen C-endopeptidase enhancer protein	2.19
1419091_a_at	Anxa2	annexin A2	2.50
1427883_a_at	Col3a1	collagen, type III, alpha 1	11.26
1423607_at	Lum	lumican	3.84
1424131_at	Col6a3	collagen, type VI, alpha 3	3.79
1424051_at	Col4a2	collagen, type IV, alpha 2	3.41
1449335_at	Timp3	tissue inhibitor of metalloproteinase 3	3.85
1419088_at	Timp3	tissue inhibitor of metalloproteinase 3	3.80

1419089_at	Timp3	tissue inhibitor of metalloproteinase 3	2.73
1448228_at	Lox	lysyl oxidase	5.68
1423516_a_at	Nid2	nidogen 2	2.51
**Condensed chromosome, centromeric region GO:779 (N=6; P=5.23e-07)**			
1424046_at	Bub1	budding uninhibited by benzimidazoles 1 homolog (*S. cerevisiae*)	4.21
1419943_s_at	Ccnb1	cyclin B1	5.38
1448205_at	Ccnb1	cyclin B1	4.28
1450842_a_at	Cenpa	centromere protein A	4.49
1439510_at	Sgol1	shugoshin-like 1 (*S. pombe*)	2.32
1435005_at	Cenpe	centromere protein E	2.02
1416961_at	Bub1b	budding uninhibited by benzimidazoles 1 homolog, beta (*S. cerevisiae*)	2.73
1447363_s_at	Bub1b	budding uninhibited by benzimidazoles 1 homolog, beta (*S. cerevisiae*)	4.77
**Condensed nuclear chromosome kinetochore GO:778 (N=4; P=7.38e-07)**			
1419943_s_at	Ccnb1	cyclin B1	5.38
1448205_at	Ccnb1	cyclin B1	4.28
1450842_a_at	Cenpa	centromere protein A	4.49
1435005_at	Cenpe	centromere protein E	2.02
1416961_at	Bub1b	budding uninhibited by benzimidazoles 1 homolog, beta (*S. cerevisiae*)	2.73
1447363_s_at	Bub1b	budding uninhibited by benzimidazoles 1 homolog, beta (*S. cerevisiae*)	4.77
**Collagen GO:5581 (N=8; P=7.38e-07)**			
1423607_at	Lum	lumican	3.84
1452968_at	Cthrc1	collagen triple helix repeat containing 1	4.74
1423110_at	Col1a2	collagen, type I, alpha 2	10.63
1450857_a_at	Col1a2	collagen, type I, alpha 2	6.37
1424051_at	Col4a2	collagen, type IV, alpha 2	3.41
1424131_at	Col6a3	collagen, type VI, alpha 3	3.79
1437165_a_at	Pcolce	procollagen C-endopeptidase enhancer protein	4.80

1448433_a_at	Pcolce	procollagen C-endopeptidase enhancer protein	2.19
1448228_at	Lox	lysyl oxidase	5.68
1427883_a_at	Col3a1	collagen, type III, alpha 1	11.26

Table S4. The gene expression changes in *Cstb*$^{-/-}$ P5+2 cerebellar granule cells. Fold change with cutoff 1.3 and $p < 0.01$ was used.

Probe Set ID	Gene Symbol	Fold change([KO]/[WT])	Unigene(Avadis)
1451194_at	Aldob	-1.36	Mm.218862
1456260_at	Rbbp4	-1.36	Mm.12145
1456444_at	Fbxo41	-1.41	Mm.38777
1446643_at	5330409N07Rik	-1.47	Mm.215279
1436155_at	Nmnat2	-1.62	Mm.40548
1439660_at	Hivep3	-1.72	Mm.302758
1442122_at	AI451458	-1.73	Mm.32470
1440570_at	NA	-1.85	Mm.209705
1458054_at	Ext1	-2.05	Mm.309395
1442945_at	2010109K09Rik	-2.33	Mm.45647
1422507_at	Cstb	-3.62	Mm.6095
1422506_a_at	Cstb	-4.23	Mm.6095
1434173_s_at	D19Bwg1357e	1.33	Mm.261027
1415754_at	Polr2f	1.34	Mm.279861
1460201_a_at	Rpl24	1.34	Mm.458082
1424242_at	Bphl	1.34	Mm.140243
1448967_at	Nipsnap3a	1.36	Mm.38244
1448103_s_at	Nono	1.36	Mm.280069
1421115_a_at	Zdhhc16	1.36	Mm.20387
1428331_at	2210016F16Rik	1.36	Mm.296610
1448238_at	2700060E02Rik	1.37	Mm.21932
1449181_at	Fech	1.37	Mm.1070
1424169_at	Tax1bp3	1.41	Mm.371656
1455693_x_at	Rps6	1.46	Mm.390690
1417583_a_at	Utp3	1.46	Mm.88546
1416416_x_at	Gstm1	1.47	Mm.37199
1455043_at	Tnpo1	1.48	Mm.173286
1416719_a_at	Rps10	1.48	Mm.275810
1438723_a_at	Rps10	1.49	Mm.275810
1424107_at	Kif18a	1.50	Mm.274086
1452226_at	Rcc2	1.53	Mm.473710
1417773_at	Nans	1.58	Mm.249349
1420478_at	Nap1l1	1.59	Mm.290407
1448501_at	Tspan6	1.64	Mm.46701
1448319_at	Akr1b3	1.68	Mm.389126
1418816_at	Chmp1b	1.69	Mm.73777
1448650_a_at	Pole	1.70	Mm.35061
1416167_at	Prdx4	1.71	Mm.247542
1417886_at	1810009A15Rik	1.72	Mm.27503
1416064_a_at	Hspa5	1.73	Mm.330160
1422441_x_at	Cdk4	1.75	Mm.6839
1424574_at	Tmed5	1.77	Mm.363960
1438320_s_at	Mcm7	1.80	Mm.378965

1435429_x_at	Rps27l	1.80	Mm.30120
1423254_x_at	Rps27l	1.80	Mm.30120
1416440_at	Cd164	1.81	Mm.269815
1433685_a_at	6430706D22Rik	1.85	Mm.384762
1448594_at	Wisp1	1.85	Mm.10222
1450925_a_at	Rps27l	1.87	Mm.30120
1438650_x_at	Gja1	1.88	Mm.378921
1448592_at	Crtap	1.88	Mm.20904
1423809_at	Tcf19	1.91	Mm.11434
1437889_x_at	Bgn	1.93	Mm.2608
1448889_at	Slc38a4	1.94	Mm.250980
1425528_at	Prrx1	1.96	Mm.288642
1423110_at	Col1a2	10.63	Mm.277792
1427883_a_at	Col3a1	11.26	Mm.249555
1435005_at	Cenpe	2.02	Mm.161470
1417082_at	Anp32b	2.03	Mm.263913
1435695_a_at	A030007L17Rik	2.07	Mm.294708
1422430_at	Fignl1	2.08	Mm.236114
1438115_a_at	Slc9a3r1	2.12	Mm.27842
1428585_at	Actn1	2.13	Mm.253564
1423813_at	Kif22	2.13	Mm.458209
1452598_at	Gins1	2.16	Mm.271603
1426858_at	Inhbb	2.16	Mm.3092
1417777_at	Ltb4dh	2.18	Mm.34497
1448433_a_at	Pcolce	2.19	Mm.262345
1425567_a_at	Anxa5	2.19	Mm.1620
1457823_at	Cyr61	2.20	Mm.1231
1416431_at	Tubb6	2.24	Mm.181860
1437580_s_at	Nek2	2.25	Mm.33773
1439510_at	Sgol1	2.32	Mm.153202
1417394_at	Klf4	2.37	Mm.473692
1437626_at	Zfp36l2	2.38	Mm.259321
1415945_at	Mcm5	2.40	Mm.5048
1425811_a_at	Csrp1	2.42	Mm.196484
1448627_s_at	Pbk	2.43	Mm.24337
1415860_at	Kpna2	2.46	Mm.12508
1450156_a_at	Hmmr	2.46	Mm.116997
1424292_at	Depdc1a	2.47	Mm.371655
1419091_a_at	Anxa2	2.50	Mm.238343
1438434_at	Arhgap11a	2.50	Mm.473800
1423516_a_at	Nid2	2.51	Mm.20348
1417323_at	Psrc1	2.58	Mm.389499
1456733_x_at	Serpinh1	2.59	Mm.22708
1428481_s_at	Cdca8	2.64	Mm.28038
1419153_at	2810417H13Rik	2.65	Mm.351273
1416961_at	Bub1b	2.73	Mm.29133

1419089_at	Timp3	2.73	Mm.4871
1452315_at	Kif11	2.76	Mm.42203
1424105_a_at	Pttg1	2.86	Mm.6856
1434767_at	C79407	2.87	Mm.21144
1449060_at	Kif2c	2.88	Mm.247651
1416405_at	Bgn	3.00	Mm.2608
1437251_at	Cdca2	3.02	Mm.33831
1431004_at	LOC100047339	3.09	Mm.116714
1425964_x_at	Hspb1	3.11	Mm.473688
1423775_s_at	Prc1	3.17	Mm.227274
1424051_at	Col4a2	3.41	Mm.181021
1425028_a_at	Tpm2	3.48	Mm.646
1439377_x_at	Cdc20	3.53	Mm.289747
1454862_at	Phldb2	3.59	Mm.211477
1448314_at	Cdc2a	3.61	Mm.281367
1448259_at	Fstl1	3.65	Mm.182434
1428105_at	Tpx2	3.67	Mm.407737
1416039_x_at	Cyr61	3.72	Mm.1231
1450981_at	Cnn2	3.74	Mm.157770
1454694_a_at	Top2a	3.77	Mm.4237
1424131_at	Col6a3	3.79	Mm.7562
1419088_at	Timp3	3.80	Mm.4871
1423607_at	Lum	3.84	Mm.18888
1449335_at	Timp3	3.85	Mm.4871
1417065_at	Egr1	3.98	Mm.181959
1450780_s_at	Hmga2	4.07	Mm.157190
1416529_at	Emp1	4.17	Mm.182785
1422851_at	Hmga2	4.21	Mm.157190
1424046_at	Bub1	4.21	Mm.2185
1417457_at	Cks2	4.27	Mm.443327
1448205_at	Ccnb1 /// Ccnb1-rs1	4.28	Mm.260114
1417458_s_at	Cks2	4.35	Mm.443327
1419149_at	Serpine1	4.39	Mm.250422
1450842_a_at	Cenpa	4.49	Mm.290563
1453593_at	Vgll3	4.51	Mm.25670
1450377_at	Thbs1	4.65	Mm.4159
1452968_at	Cthrc1	4.74	Mm.41556
1416454_s_at	Acta2	4.75	Mm.213025
1447363_s_at	Bub1b	4.77	Mm.29133
1437165_a_at	Pcolce	4.80	Mm.262345
1450920_at	Ccnb2	5.05	Mm.22592
1416309_at	Nusap1	5.09	Mm.290015
1423505_at	Tagln	5.25	Mm.283283
1419943_s_at	Ccnb1	5.38	Mm.380027
1448228_at	Lox	5.68	Mm.172
1448213_at	Anxa1	6.10	Mm.248360

1460302_at	Thbs1	6.33	Mm.4159
1450857_a_at	Col1a2	6.37	Mm.277792
1421811_at	Thbs1	7.35	Mm.4159
1416953_at	Ctgf	8.08	Mm.393058
1448194_a_at	H19	8.36	Mm.14802

Table S5. The gene expression changes in P30 *Cstb*$^{-/-}$ cerebellum. Fold change with cutoff 1.3 and $p < 0.05$ was used.

Probe Set ID	Gene Symbol	Fold change([KO]/ [WT])	Unigene(Avadis)
1432014_at	6530409C15Rik	-1.34	Mm.425312
1433344_at	4930448K20Rik	-1.39	Mm.195665
1452533_at	Ryr3	-1.54	Mm.436657
1422506_a_at	Cstb	-6.48	Mm.6095
1422507_at	Cstb	-6.82	Mm.6095
1427301_at	Cd48	1.30	Mm.1738
1452961_at	Tril	1.32	Mm.471774
1455400_at	Ddah1	1.32	Mm.234247
1460604_at	Cybrd1	1.32	Mm.45435
1420502_at	Sat1	1.33	Mm.2734
1448139_at	Mlc1	1.33	Mm.32780
1448148_at	Grn	1.33	Mm.1568
1420911_a_at	Mfge8	1.33	Mm.1451
1427256_at	Vcan	1.33	Mm.158700
1422788_at	Slc43a3	1.33	Mm.290729
1427643_at	Gm16499	1.34	Mm.422727
1448118_a_at	Ctsd	1.34	Mm.231395
1434891_at	Ptgfrn	1.34	Mm.24807
1433678_at	Pld4	1.34	Mm.203915
1424534_at	Mmd2	1.34	Mm.48712
1437312_at	Bmpr1b	1.36	Mm.39089
1437347_at	Ednrb	1.36	Mm.229532
1449514_at	Grk5	1.37	Mm.279400
1438629_x_at	Grn	1.38	Mm.1568
1422615_at	Map4k4	1.38	Mm.19073
1438654_x_at	Mmd2	1.38	Mm.48712
1456567_x_at	Grn	1.41	Mm.1568
1437390_x_at	Stx1a	1.41	Mm.6225
1424944_at	Pcp2	1.43	Mm.440882
1417483_at	Nfkbiz	1.44	Mm.247272
1457148_at	Csmd2	1.45	Mm.442507
1420361_at	Slc11a1	1.45	Mm.2913
1447745_at	Aqp4	1.47	Mm.250786
1436779_at	Cybb	1.49	Mm.200362
1416066_at	Cd9	1.50	Mm.210676
1417141_at	Igtp	1.50	Mm.33902
1448162_at	Vcam1	1.50	Mm.76649
1426025_s_at	Laptm5	1.52	Mm.271868
1418204_s_at	Aif1	1.53	Mm.10747
1433575_at	Sox4	1.54	Mm.240627
1442082_at	C3ar1	1.55	Mm.2408
1420895_at	Tgfbr1	1.56	Mm.197552
1448475_at	Olfml3	1.58	Mm.211535

1456685_at	Nsg2	1.59	Mm.3304
1426278_at	Ifi27	1.60	Mm.271275
1448748_at	Plek	1.62	Mm.98232
1427746_x_at	H2-K1	1.62	Mm.439675
1443814_x_at	Ctsh	1.64	Mm.2277
1438658_a_at	Edg3	1.72	Mm.136736
1418365_at	Ctsh	1.74	Mm.2277
1448124_at	Gusb	1.81	Mm.3317
1419483_at	C3ar1	1.84	Mm.2408
1419872_at	Csf1r	1.87	Mm.22574
1431056_a_at	Lpl	1.95	Mm.1514
1418340_at	Fcer1g	2.00	Mm.22673
1450678_at	Itgb2	2.02	Mm.1137
1417266_at	Ccl6	2.15	Mm.137
1452428_a_at	B2m	2.17	Mm.163
1418021_at	C4b	2.23	Mm.472690
1426808_at	Lgals3	2.24	Mm.248615
1437874_s_at	Hexb	2.26	Mm.27816
1448620_at	Fcgr3	2.30	Mm.22119
1449289_a_at	B2m	2.32	Mm.163
1436314_at	Scyl2	2.33	Mm.27651
1417268_at	Cd14	2.66	Mm.3460
1449164_at	Cd68	2.75	Mm.15819
1423760_at	Cd44	2.76	Mm.423621
1422903_at	Ly86	3.13	Mm.2639
1434376_at	Cd44	3.17	Mm.423621
1440142_s_at	Gfap	3.35	Mm.1239
1449401_at	C1qc	3.38	Mm.439732
1417381_at	C1qa	3.56	Mm.439957
1427076_at	Mpeg1	3.80	Mm.3999
1415904_at	Lpl	3.90	Mm.1514
1417063_at	C1qb	4.18	Mm.2570
1460218_at	Cd52	4.23	Mm.24130
1437726_x_at	C1qb	4.26	Mm.2570
1426508_at	Gfap	4.45	Mm.1239
1450792_at	Tyrobp	4.61	Mm.46301
1448859_at	Cxcl13	5.09	Mm.10116
1426509_s_at	Gfap	5.15	Mm.1239
1417851_at	Cxcl13	51.92	Mm.10116

Table S6. The GO terms of biological process, molecular function and cellular component in P30 *Cstb*$^{-/-}$ cerebellum. N indicates the number of genes and P is the adjusted p-value for the enrichment.

BIOLOGICAL PROCESSES

Probe ID	Gene symbol	Gene title	Fold change
**Immune system process GO:2376 (N=28; P=2.72e-12)**			
1422903_at	Ly86	lymphocyte antigen 86	3.13
1419483_at	C3ar1	complement component 3a receptor 1	1.84
1442082_at	C3ar1	complement component 3a receptor 1	1.55
1417266_at	Ccl6	chemokine (C-C motif) ligand 6	2.15
1417268_at	Cd14	CD14 antigen	2.66
1427746_x_at	H2-K1	histocompatibility 2, K1, K region	1.62
1437347_at	Ednrb	endothelin receptor type B	1.36
1450792_at	Tyrobp	TYRO protein tyrosine kinase binding protein	4.61
1452428_a_at	B2m	beta-2 microglobulin	2.17
1449289_a_at	B2m	beta-2 microglobulin	2.32
1450678_at	Itgb2	integrin beta 2	2.02
1449401_at	C1qc	complement component 1, q subcomponent, C chain	3.38
1452961_at	Tril	TLR4 interactor with leucine-rich repeats	1.32
1418340_at	Fcer1g	Fc receptor, IgE, high affinity I, gamma polypeptide	2.00
1436779_at	Cybb	cytochrome b-245, beta polypeptide	1.49
1420361_at	Slc11a1	solute carrier family 11 (proton-coupled divalent metal ion transporters), member 1	1.45
1418365_at	Ctsh	cathepsin H	1.74
1443814_x_at	Ctsh	cathepsin H	1.64
1417381_at	C1qa	complement component 1, q subcomponent, alpha polypeptide	3.56
1420895_at	Tgfbr1	transforming growth factor, beta receptor I	1.56
1417063_at	C1qb	complement component 1, q subcomponent, beta polypeptide	4.18

1437726_x_at	C1qb	complement component 1, q subcomponent, beta polypeptide	4.26
1418204_s_at	Aif1	allograft inflammatory factor 1	1.53
1433575_at	Sox4	SRY-box containing gene 4	1.54
1418021_at	C4b	complement component 4B (Chido blood group)	2.23
1448620_at	Fcgr3	Fc receptor, IgG, low affinity III	2.30
1419872_at	Csf1r	colony stimulating factor 1 receptor	1.87
1448162_at	Vcam1	vascular cell adhesion molecule 1	1.50
1434376_at	Cd44	CD44 antigen	3.17
1423760_at	Cd44	CD44 antigen	2.76
1417851_at	Cxcl13	chemokine (C-X-C motif) ligand 13	51.92
1448859_at	Cxcl13	chemokine (C-X-C motif) ligand 13	5.09
1447745_at	Aqp4	aquaporin 4	1.47
1427301_at	Cd48	CD48 antigen	1.30
**Immune response GO:6955 (N=21; P=5.63e-12)**			
1422903_at	Ly86	lymphocyte antigen 86	3.13
1419483_at	C3ar1	complement component 3a receptor 1	1.84
1442082_at	C3ar1	complement component 3a receptor 1	1.55
1417266_at	Ccl6	chemokine (C-C motif) ligand 6	2.15
1417268_at	Cd14	CD14 antigen	2.66
1427746_x_at	H2-K1	histocompatibility 2, K1, K region	1.62
1450792_at	Tyrobp	TYRO protein tyrosine kinase binding protein	4.61
1452428_a_at	B2m	beta-2 microglobulin	2.17
1449289_a_at	B2m	beta-2 microglobulin	2.32
1449401_at	C1qc	complement component 1, q subcomponent, C chain	3.38
1452961_at	Tril	TLR4 interactor with leucine-rich repeats	1.32
1418340_at	Fcer1g	Fc receptor, IgE, high affinity I, gamma polypeptide	2.00
1436779_at	Cybb	cytochrome b-245, beta polypeptide	1.49

1420361_at	Slc11a1	solute carrier family 11 (proton-coupled divalent metal ion transporters), member 1	1.45
1418365_at	Ctsh	cathepsin H	1.74
1443814_x_at	Ctsh	cathepsin H	1.64
1417381_at	C1qa	complement component 1, q subcomponent, alpha polypeptide	3.56
1417063_at	C1qb	complement component 1, q subcomponent, beta polypeptide	4.18
1437726_x_at	C1qb	complement component 1, q subcomponent, beta polypeptide	4.26
1418021_at	C4b	complement component 4B (Chido blood group)	2.23
1448620_at	Fcgr3	Fc receptor, IgG, low affinity III	2.30
1419872_at	Csf1r	colony stimulating factor 1 receptor	1.87
1434376_at	Cd44	CD44 antigen	3.17
1423760_at	Cd44	CD44 antigen	2.76
1417851_at	Cxcl13	chemokine (C-X-C motif) ligand 13	51.92
1448859_at	Cxcl13	chemokine (C-X-C motif) ligand 13	5.09
1447745_at	Aqp4	aquaporin 4	1.47
**Adaptive immune response GO:2250 (N=12; P=3.61e-10)**			
1419483_at	C3ar1	complement component 3a receptor 1	1.84
1442082_at	C3ar1	complement component 3a receptor 1	1.55
1417063_at	C1qb	complement component 1, q subcomponent, beta polypeptide	4.18
1437726_x_at	C1qb	complement component 1, q subcomponent, beta polypeptide	4.26
1427746_x_at	H2-K1	histocompatibility 2, K1, K region	1.62
1418021_at	C4b	complement component 4B (Chido blood group)	2.23
1448620_at	Fcgr3	Fc receptor, IgG, low affinity III	2.30
1452428_a_at	B2m	beta-2 microglobulin	2.17
1449289_a_at	B2m	beta-2 microglobulin	2.32
1434376_at	Cd44	CD44 antigen	3.17
1423760_at	Cd44	CD44 antigen	2.76
1449401_at	C1qc	complement component 1, q subcomponent, C chain	3.38

1420361_at	Slc11a1	solute carrier family 11 (proton-coupled divalent metal ion transporters), member 1	1.45
1418340_at	Fcer1g	Fc receptor, IgE, high affinity I, gamma polypeptide	2.00
1418365_at	Ctsh	cathepsin H	1.74
1443814_x_at	Ctsh	cathepsin H	1.64
1417381_at	C1qa	complement component 1, q subcomponent, alpha polypeptide	3.56
**Defense response GO:6952 (N=20; P=3.61e-10)**			
1422903_at	Ly86	lymphocyte antigen 86	3.13
1438658_a_at	S1pr3	sphingosine-1-phosphate receptor 3	1.72
1417268_at	Cd14	CD14 antigen	2.66
1427746_x_at	H2-K1	histocompatibility 2, K1, K region	1.62
1452428_a_at	B2m	beta-2 microglobulin	2.17
1449289_a_at	B2m	beta-2 microglobulin	2.32
1450678_at	Itgb2	integrin beta 2	2.02
1449401_at	C1qc	complement component 1, q subcomponent, C chain	3.38
1452961_at	Tril	TLR4 interactor with leucine-rich repeats	1.32
1436779_at	Cybb	cytochrome b-245, beta polypeptide	1.49
1420361_at	Slc11a1	solute carrier family 11 (proton-coupled divalent metal ion transporters), member 1	1.45
1418340_at	Fcer1g	Fc receptor, IgE, high affinity I, gamma polypeptide	2.00
1417381_at	C1qa	complement component 1, q subcomponent, alpha polypeptide	3.56
1417483_at	Nfkbiz	nuclear factor of kappa light polypeptide gene enhancer in B cells inhibitor, zeta	1.44
1417063_at	C1qb	complement component 1, q subcomponent, beta polypeptide	4.18
1437726_x_at	C1qb	complement component 1, q subcomponent, beta polypeptide	4.26
1418021_at	C4b	complement component 4B (Chido blood group)	2.23
1448620_at	Fcgr3	Fc receptor, IgG, low affinity III	2.30
1419872_at	Csf1r	colony stimulating factor 1 receptor	1.87
1434376_at	Cd44	CD44 antigen	3.17
1423760_at	Cd44	CD44 antigen	2.76

1417851_at	Cxcl13	chemokine (C-X-C motif) ligand 13	51.92
1448859_at	Cxcl13	chemokine (C-X-C motif) ligand 13	5.09
1447745_at	Aqp4	aquaporin 4	1.47
**Response to stimulus GO:50896 (N=48; P=3.61e-10)**			
1419483_at	C3ar1	complement component 3a receptor 1	1.84
1442082_at	C3ar1	complement component 3a receptor 1	1.55
1438658_a_at	S1pr3	sphingosine-1-phosphate receptor 3	1.72
1448118_a_at	Ctsd	cathepsin D	1.34
1456567_x_at	Grn	granulin	1.41
1448148_at	Grn	granulin	1.33
1438629_x_at	Grn	granulin	1.38
1417268_at	Cd14	CD14 antigen	2.66
1437347_at	Ednrb	endothelin receptor type B	1.36
1422507_at	Cstb	cystatin B	-6.82
1422506_a_at	Cstb	cystatin B	-6.48
1450792_at	Tyrobp	TYRO protein tyrosine kinase binding protein	4.61
1452428_a_at	B2m	beta-2 microglobulin	2.17
1449289_a_at	B2m	beta-2 microglobulin	2.32
1426278_at	Ifi27l2a	interferon, alpha-inducible protein 27 like 2A	1.60
1422615_at	Map4k4	mitogen-activated protein kinase kinase kinase kinase 4	1.38
1449401_at	C1qc	complement component 1, q subcomponent, C chain	3.38
1437874_s_at	Hexb	hexosaminidase B	2.26
1417141_at	Igtp	interferon gamma induced GTPase	1.50
1436779_at	Cybb	cytochrome b-245, beta polypeptide	1.49
1420361_at	Slc11a1	solute carrier family 11 (proton-coupled divalent metal ion transporters), member 1	1.45
1424944_at	Pcp2	Purkinje cell protein 2 (L7)	1.43
1448139_at	Mlc1	megalencephalic leukoencephalopathy with subcortical cysts 1 homolog (human)	1.33

1418204_s_at	Aif1	allograft inflammatory factor 1	1.53
1433575_at	Sox4	SRY-box containing gene 4	1.54
1418021_at	C4b	complement component 4B (Chido blood group)	2.23
1448162_at	Vcam1	vascular cell adhesion molecule 1	1.50
1436314_at	Scyl2	SCY1-like 2 (*S. cerevisiae*)	2.33
1456685_at	Nsg2	neuron specific gene family member 2	1.59
1422903_at	Ly86	lymphocyte antigen 86	3.13
1460604_at	Cybrd1	cytochrome b reductase 1	1.32
1417266_at	Ccl6	chemokine (C-C motif) ligand 6	2.15
1427746_x_at	H2-K1	histocompatibility 2, K1, K region	1.62
1449164_at	Cd68	CD68 antigen	2.75
1450678_at	Itgb2	integrin beta 2	2.02
1452961_at	Tril	TLR4 interactor with leucine-rich repeats	1.32
1418340_at	Fcer1g	Fc receptor, IgE, high affinity I, gamma polypeptide	2.00
1449514_at	Grk5	G protein-coupled receptor kinase 5	1.37
1418365_at	Ctsh	cathepsin H	1.74
1443814_x_at	Ctsh	cathepsin H	1.64
1417381_at	C1qa	complement component 1, q subcomponent, alpha polypeptide	3.56
1420895_at	Tgfbr1	transforming growth factor, beta receptor I	1.56
1417483_at	Nfkbiz	nuclear factor of kappa light polypeptide gene enhancer in B cells inhibitor, zeta	1.44
1437312_at	Bmpr1b	bone morphogenetic protein receptor, type 1B	1.36
1417063_at	C1qb	complement component 1, q subcomponent, beta polypeptide	4.18
1437726_x_at	C1qb	complement component 1, q subcomponent, beta polypeptide	4.26
1426509_s_at	Gfap	glial fibrillary acidic protein	5.15
1440142_s_at	Gfap	glial fibrillary acidic protein	3.35
1426508_at	Gfap	glial fibrillary acidic protein	4.45
1448620_at	Fcgr3	Fc receptor, IgG, low affinity III	2.30

1419872_at	Csf1r	colony stimulating factor 1 receptor	1.87
1434376_at	Cd44	CD44 antigen	3.17
1423760_at	Cd44	CD44 antigen	2.76
1417851_at	Cxcl13	chemokine (C-X-C motif) ligand 13	51.92
1448859_at	Cxcl13	chemokine (C-X-C motif) ligand 13	5.09
1447745_at	Aqp4	aquaporin 4	1.47
1437390_x_at	Stx1a	syntaxin 1A (brain)	1.41
1448748_at	Plek	pleckstrin	1.62
1427301_at	Cd48	CD48 antigen	1.30
**Adaptive immune response based on somatic recombination of immune receptors built from immunoglobulin superfamily domains GO:2460 (N=11; P=2.97e-09)**			
1419483_at	C3ar1	complement component 3a receptor 1	1.84
1442082_at	C3ar1	complement component 3a receptor 1	1.55
1417063_at	C1qb	complement component 1, q subcomponent, beta polypeptide	4.18
1437726_x_at	C1qb	complement component 1, q subcomponent, beta polypeptide	4.26
1427746_x_at	H2-K1	histocompatibility 2, K1, K region	1.62
1418021_at	C4b	complement component 4B (Chido blood group)	2.23
1448620_at	Fcgr3	Fc receptor, IgG, low affinity III	2.30
1452428_a_at	B2m	beta-2 microglobulin	2.17
1449289_a_at	B2m	beta-2 microglobulin	2.32
1449401_at	C1qc	complement component 1, q subcomponent, C chain	3.38
1420361_at	Slc11a1	solute carrier family 11 (proton-coupled divalent metal ion transporters), member 1	1.45
1418340_at	Fcer1g	Fc receptor, IgE, high affinity I, gamma polypeptide	2.00
1418365_at	Ctsh	cathepsin H	1.74
1443814_x_at	Ctsh	cathepsin H	1.64
1417381_at	C1qa	complement component 1, q subcomponent, alpha polypeptide	3.56
**Positive regulation of immune system process GO:2684 (N=15; P=5.23e-09)**			
1419483_at	C3ar1	complement component 3a receptor 1	1.84

1442082_at	C3ar1	complement component 3a receptor 1	1.55
1417063_at	C1qb	complement component 1, q subcomponent, beta polypeptide	4.18
1437726_x_at	C1qb	complement component 1, q subcomponent, beta polypeptide	4.26
1418204_s_at	Aif1	allograft inflammatory factor 1	1.53
1427746_x_at	H2-K1	histocompatibility 2, K1, K region	1.62
1418021_at	C4b	complement component 4B (Chido blood group)	2.23
1448620_at	Fcgr3	Fc receptor, IgG, low affinity III	2.30
1452428_a_at	B2m	beta-2 microglobulin	2.17
1449289_a_at	B2m	beta-2 microglobulin	2.32
1448162_at	Vcam1	vascular cell adhesion molecule 1	1.50
1434376_at	Cd44	CD44 antigen	3.17
1423760_at	Cd44	CD44 antigen	2.76
1449401_at	C1qc	complement component 1, q subcomponent, C chain	3.38
1452961_at	Tril	TLR4 interactor with leucine-rich repeats	1.32
1420361_at	Slc11a1	solute carrier family 11 (proton-coupled divalent metal ion transporters), member 1	1.45
1418340_at	Fcer1g	Fc receptor, IgE, high affinity I, gamma polypeptide	2.00
1417851_at	Cxcl13	chemokine (C-X-C motif) ligand 13	51.92
1448859_at	Cxcl13	chemokine (C-X-C motif) ligand 13	5.09
1417381_at	C1qa	complement component 1, q subcomponent, alpha polypeptide	3.56
**Positive regulation of immune response GO:50778 (N=12; P=1.98e-08)**			
1419483_at	C3ar1	complement component 3a receptor 1	1.84
1442082_at	C3ar1	complement component 3a receptor 1	1.55
1417063_at	C1qb	complement component 1, q subcomponent, beta polypeptide	4.18
1437726_x_at	C1qb	complement component 1, q subcomponent, beta polypeptide	4.26
1427746_x_at	H2-K1	histocompatibility 2, K1, K region	1.62
1418021_at	C4b	complement component 4B (Chido blood group)	2.23
1448620_at	Fcgr3	Fc receptor, IgG, low affinity III	2.30

1452428_a_at	B2m	beta-2 microglobulin	2.17
1449289_a_at	B2m	beta-2 microglobulin	2.32
1434376_at	Cd44	CD44 antigen	3.17
1423760_at	Cd44	CD44 antigen	2.76
1452961_at	Tril	TLR4 interactor with leucine-rich repeats	1.32
1449401_at	C1qc	complement component 1, q subcomponent, C chain	3.38
1420361_at	Slc11a1	solute carrier family 11 (proton-coupled divalent metal ion transporters), member 1	1.45
1418340_at	Fcer1g	Fc receptor, IgE, high affinity I, gamma polypeptide	2.00
1417381_at	C1qa	complement component 1, q subcomponent, alpha polypeptide	3.56
**Lymphocyte mediated immunity GO:2449 (N=10; P=1.98e-08)**			
1417063_at	C1qb	complement component 1, q subcomponent, beta polypeptide	4.18
1437726_x_at	C1qb	complement component 1, q subcomponent, beta polypeptide	4.26
1427746_x_at	H2-K1	histocompatibility 2, K1, K region	1.62
1418021_at	C4b	complement component 4B (Chido blood group)	2.23
1448620_at	Fcgr3	Fc receptor, IgG, low affinity III	2.30
1452428_a_at	B2m	beta-2 microglobulin	2.17
1449289_a_at	B2m	beta-2 microglobulin	2.32
1449401_at	C1qc	complement component 1, q subcomponent, C chain	3.38
1420361_at	Slc11a1	solute carrier family 11 (proton-coupled divalent metal ion transporters), member 1	1.45
1418340_at	Fcer1g	Fc receptor, IgE, high affinity I, gamma polypeptide	2.00
1418365_at	Ctsh	cathepsin H	1.74
1443814_x_at	Ctsh	cathepsin H	1.64
1417381_at	C1qa	complement component 1, q subcomponent, alpha polypeptide	3.56
**Regulation of immune system process GO:2682 (N=17; P=2.30e-08)**			
1419483_at	C3ar1	complement component 3a receptor 1	1.84
1442082_at	C3ar1	complement component 3a receptor 1	1.55
1427746_x_at	H2-K1	histocompatibility 2, K1, K region	1.62

1452428_a_at	B2m	beta-2 microglobulin	2.17
1449289_a_at	B2m	beta-2 microglobulin	2.32
1449401_at	C1qc	complement component 1, q subcomponent, C chain	3.38
1452961_at	Tril	TLR4 interactor with leucine-rich repeats	1.32
1420361_at	Slc11a1	solute carrier family 11 (proton-coupled divalent metal ion transporters), member 1	1.45
1418340_at	Fcer1g	Fc receptor, IgE, high affinity I, gamma polypeptide	2.00
1418365_at	Ctsh	cathepsin H	1.74
1443814_x_at	Ctsh	cathepsin H	1.64
1417381_at	C1qa	complement component 1, q subcomponent, alpha polypeptide	3.56
1417063_at	C1qb	complement component 1, q subcomponent, beta polypeptide	4.18
1437726_x_at	C1qb	complement component 1, q subcomponent, beta polypeptide	4.26
1418204_s_at	Aif1	allograft inflammatory factor 1	1.53
1418021_at	C4b	complement component 4B (Chido blood group)	2.23
1448620_at	Fcgr3	Fc receptor, IgG, low affinity III	2.30
1448162_at	Vcam1	vascular cell adhesion molecule 1	1.50
1419872_at	Csf1r	colony stimulating factor 1 receptor	1.87
1434376_at	Cd44	CD44 antigen	3.17
1423760_at	Cd44	CD44 antigen	2.76
1417851_at	Cxcl13	chemokine (C-X-C motif) ligand 13	51.92
1448859_at	Cxcl13	chemokine (C-X-C motif) ligand 13	5.09

MOLECULAR FUNCTION

**Protein complex binding GO:32403 (N=10; P=7.12e-05)**			
1448139_at	Mlc1	megalencephalic leukoencephalopathy with subcortical cysts 1 homolog (human)	1.33
1448620_at	Fcgr3	Fc receptor, IgG, low affinity III	2.30
1426509_s_at	Gfap	glial fibrillary acidic protein	5.15
1440142_s_at	Gfap	glial fibrillary acidic protein	3.35

1426508_at	Gfap	glial fibrillary acidic protein	4.45
1448162_at	Vcam1	vascular cell adhesion molecule 1	1.50
1450678_at	Itgb2	integrin beta 2	2.02
1416066_at	Cd9	CD9 antigen	1.50
1420911_a_at	Mfge8	milk fat globule-EGF factor 8 protein	1.33
1418340_at	Fcer1g	Fc receptor, IgE, high affinity I, gamma polypeptide	2.00
1418365_at	Ctsh	cathepsin H	1.74
1443814_x_at	Ctsh	cathepsin H	1.64
1420895_at	Tgfbr1	transforming growth factor, beta receptor I	1.56
**Protein binding GO:5515 (N=35; P=0.0009)**			
1419483_at	C3ar1	complement component 3a receptor 1	1.84
1442082_at	C3ar1	complement component 3a receptor 1	1.55
1422507_at	Cstb	cystatin B	-6.82
1422506_a_at	Cstb	cystatin B	-6.48
1433678_at	Pld4	phospholipase D family, member 4	1.34
1437347_at	Ednrb	endothelin receptor type B	1.36
1450792_at	Tyrobp	TYRO protein tyrosine kinase binding protein	4.61
1452428_a_at	B2m	beta-2 microglobulin	2.17
1449289_a_at	B2m	beta-2 microglobulin	2.32
1437874_s_at	Hexb	hexosaminidase B	2.26
1436779_at	Cybb	cytochrome b-245, beta polypeptide	1.49
1420361_at	Slc11a1	solute carrier family 11 (proton-coupled divalent metal ion transporters), member 1	1.45
1426808_at	Lgals3	lectin, galactose binding, soluble 3	2.24
1448139_at	Mlc1	megalencephalic leukoencephalopathy with subcortical cysts 1 homolog (human)	1.33
1418204_s_at	Aif1	allograft inflammatory factor 1	1.53
1448162_at	Vcam1	vascular cell adhesion molecule 1	1.50
1436314_at	Scyl2	SCY1-like 2 (*S. cerevisiae*)	2.33

1456685_at	Nsg2	neuron specific gene family member 2	1.59
1420911_a_at	Mfge8	milk fat globule-EGF factor 8 protein	1.33
1452533_at	Ryr3	ryanodine receptor 3	-1.54
1417266_at	Ccl6	chemokine (C-C motif) ligand 6	2.15
1450678_at	Itgb2	integrin beta 2	2.02
1418340_at	Fcer1g	Fc receptor, IgE, high affinity I, gamma polypeptide	2.00
1418365_at	Ctsh	cathepsin H	1.74
1443814_x_at	Ctsh	cathepsin H	1.64
1417381_at	C1qa	complement component 1, q subcomponent, alpha polypeptide	3.56
1420895_at	Tgfbr1	transforming growth factor, beta receptor I	1.56
1437312_at	Bmpr1b	bone morphogenetic protein receptor, type 1B	1.36
1417063_at	C1qb	complement component 1, q subcomponent, beta polypeptide	4.18
1437726_x_at	C1qb	complement component 1, q subcomponent, beta polypeptide	4.26
1415904_at	Lpl	lipoprotein lipase	3.90
1431056_a_at	Lpl	lipoprotein lipase	1.95
1426509_s_at	Gfap	glial fibrillary acidic protein	5.15
1440142_s_at	Gfap	glial fibrillary acidic protein	3.35
1426508_at	Gfap	glial fibrillary acidic protein	4.45
1448620_at	Fcgr3	Fc receptor, IgG, low affinity III	2.30
1419872_at	Csf1r	colony stimulating factor 1 receptor	1.87
1434376_at	Cd44	CD44 antigen	3.17
1423760_at	Cd44	CD44 antigen	2.76
1416066_at	Cd9	CD9 antigen	1.50
1417851_at	Cxcl13	chemokine (C-X-C motif) ligand 13	51.92
1448859_at	Cxcl13	chemokine (C-X-C motif) ligand 13	5.09
1437390_x_at	Stx1a	syntaxin 1A (brain)	1.41
1448748_at	Plek	pleckstrin	1.62

1427301_at	Cd48	CD48 antigen	1.30
**Receptor activity GO:4872 (N=12; P=0.0009)**			
1419483_at	C3ar1	complement component 3a receptor 1	1.84
1442082_at	C3ar1	complement component 3a receptor 1	1.55
1438658_a_at	S1pr3	sphingosine-1-phosphate receptor 3	1.72
1437312_at	Bmpr1b	bone morphogenetic protein receptor, type 1B	1.36
1437347_at	Ednrb	endothelin receptor type B	1.36
1448620_at	Fcgr3	Fc receptor, IgG, low affinity III	2.30
1419872_at	Csf1r	colony stimulating factor 1 receptor	1.87
1434376_at	Cd44	CD44 antigen	3.17
1423760_at	Cd44	CD44 antigen	2.76
1450678_at	Itgb2	integrin beta 2	2.02
1418340_at	Fcer1g	Fc receptor, IgE, high affinity I, gamma polypeptide	2.00
1418365_at	Ctsh	cathepsin H	1.74
1443814_x_at	Ctsh	cathepsin H	1.64
1427301_at	Cd48	CD48 antigen	1.30
1420895_at	Tgfbr1	transforming growth factor, beta receptor I	1.56
**Receptor binding GO:5102 (N=13; P=0.0010)**			
1415904_at	Lpl	lipoprotein lipase	3.90
1431056_a_at	Lpl	lipoprotein lipase	1.95
1417266_at	Ccl6	chemokine (C-C motif) ligand 6	2.15
1437347_at	Ednrb	endothelin receptor type B	1.36
1426509_s_at	Gfap	glial fibrillary acidic protein	5.15
1440142_s_at	Gfap	glial fibrillary acidic protein	3.35
1426508_at	Gfap	glial fibrillary acidic protein	4.45
1448162_at	Vcam1	vascular cell adhesion molecule 1	1.50
1434376_at	Cd44	CD44 antigen	3.17

1423760_at	Cd44	CD44 antigen	2.76
1450678_at	Itgb2	integrin beta 2	2.02
1416066_at	Cd9	CD9 antigen	1.50
1436314_at	Scyl2	SCY1-like 2 (*S. cerevisiae*)	2.33
1456685_at	Nsg2	neuron specific gene family member 2	1.59
1420911_a_at	Mfge8	milk fat globule-EGF factor 8 protein	1.33
1417851_at	Cxcl13	chemokine (C-X-C motif) ligand 13	51.92
1448859_at	Cxcl13	chemokine (C-X-C motif) ligand 13	5.09
1420895_at	Tgfbr1	transforming growth factor, beta receptor I	1.56
**Transmembrane signaling receptor activity GO:4888 (N=10; P=0.0010)**			
1419483_at	C3ar1	complement component 3a receptor 1	1.84
1442082_at	C3ar1	complement component 3a receptor 1	1.55
1438658_a_at	S1pr3	sphingosine-1-phosphate receptor 3	1.72
1437312_at	Bmpr1b	bone morphogenetic protein receptor, type 1B	1.36
1437347_at	Ednrb	endothelin receptor type B	1.36
1448620_at	Fcgr3	Fc receptor, IgG, low affinity III	2.30
1419872_at	Csf1r	colony stimulating factor 1 receptor	1.87
1434376_at	Cd44	CD44 antigen	3.17
1423760_at	Cd44	CD44 antigen	2.76
1418340_at	Fcer1g	Fc receptor, IgE, high affinity I, gamma polypeptide	2.00
1418365_at	Ctsh	cathepsin H	1.74
1443814_x_at	Ctsh	cathepsin H	1.64
1420895_at	Tgfbr1	transforming growth factor, beta receptor I	1.56
**IgG binding GO:19864 (N=2; P=0.0011)**			
1418340_at	Fcer1g	Fc receptor, IgE, high affinity I, gamma polypeptide	2.00
1448620_at	Fcgr3	Fc receptor, IgG, low affinity III	2.30
**Signaling receptor activity GO:38023 (N=10; P=0.0011)**			

1419483_at	C3ar1	complement component 3a receptor 1	1.84
1442082_at	C3ar1	complement component 3a receptor 1	1.55
1438658_a_at	S1pr3	sphingosine-1-phosphate receptor 3	1.72
1437312_at	Bmpr1b	bone morphogenetic protein receptor, type 1B	1.36
1437347_at	Ednrb	endothelin receptor type B	1.36
1448620_at	Fcgr3	Fc receptor, IgG, low affinity III	2.30
1419872_at	Csf1r	colony stimulating factor 1 receptor	1.87
1434376_at	Cd44	CD44 antigen	3.17
1423760_at	Cd44	CD44 antigen	2.76
1418340_at	Fcer1g	Fc receptor, IgE, high affinity I, gamma polypeptide	2.00
1418365_at	Ctsh	cathepsin H	1.74
1443814_x_at	Ctsh	cathepsin H	1.64
1420895_at	Tgfbr1	transforming growth factor, beta receptor I	1.56
**Immunoglobulin receptor activity GO:19763 (N=2; P=0.0011)**			
1418340_at	Fcer1g	Fc receptor, IgE, high affinity I, gamma polypeptide	2.00
1448620_at	Fcgr3	Fc receptor, IgG, low affinity III	2.30
**Integrin binding GO:5178 (N=4;P=0.0011)**			
1448162_at	Vcam1	vascular cell adhesion molecule 1	1.50
1416066_at	Cd9	CD9 antigen	1.50
1420911_a_at	Mfge8	milk fat globule-EGF factor 8 protein	1.33
1426509_s_at	Gfap	glial fibrillary acidic protein	5.15
1440142_s_at	Gfap	glial fibrillary acidic protein	3.35
1426508_at	Gfap	glial fibrillary acidic protein	4.45
**Transforming growth factor beta receptor activity, type I GO:5025 (N=2; P=0.0011)**			
1437312_at	Bmpr1b	bone morphogenetic protein receptor, type 1B	1.36
1420895_at	Tgfbr1	transforming growth factor, beta receptor I	1.56

CELLULAR COMPONENT

**Cell surface GO:9986 (N=15; P=7.03e-08)**			
1456567_x_at	Grn	granulin	1.41
1448148_at	Grn	granulin	1.33
1438629_x_at	Grn	granulin	1.38
1415904_at	Lpl	lipoprotein lipase	3.90
1431056_a_at	Lpl	lipoprotein lipase	1.95
1417268_at	Cd14	CD14 antigen	2.66
1427746_x_at	H2-K1	histocompatibility 2, K1, K region	1.62
1452428_a_at	B2m	beta-2 microglobulin	2.17
1449289_a_at	B2m	beta-2 microglobulin	2.32
1419872_at	Csf1r	colony stimulating factor 1 receptor	1.87
1448162_at	Vcam1	vascular cell adhesion molecule 1	1.50
1434376_at	Cd44	CD44 antigen	3.17
1423760_at	Cd44	CD44 antigen	2.76
1450678_at	Itgb2	integrin beta 2	2.02
1416066_at	Cd9	CD9 antigen	1.50
1420911_a_at	Mfge8	milk fat globule-EGF factor 8 protein	1.33
1420361_at	Slc11a1	solute carrier family 11 (proton-coupled divalent metal ion transporters), member 1	1.45
1418340_at	Fcer1g	Fc receptor, IgE, high affinity I, gamma polypeptide	2.00
1447745_at	Aqp4	aquaporin 4	1.47
1427301_at	Cd48	CD48 antigen	1.30
**Membrane part GO:44425 (N=38; P=7.31e-06)**			
1419483_at	C3ar1	complement component 3a receptor 1	1.84
1442082_at	C3ar1	complement component 3a receptor 1	1.55
1438658_a_at	S1pr3	sphingosine-1-phosphate receptor 3	1.72
1417268_at	Cd14	CD14 antigen	2.66

1437347_at	Ednrb	endothelin receptor type B	1.36
1433678_at	Pld4	phospholipase D family, member 4	1.34
1450792_at	Tyrobp	TYRO protein tyrosine kinase binding protein	4.61
1452428_a_at	B2m	beta-2 microglobulin	2.17
1449289_a_at	B2m	beta-2 microglobulin	2.32
1426278_at	Ifi27l2a	interferon, alpha-inducible protein 27 like 2A	1.60
1436779_at	Cybb	cytochrome b-245, beta polypeptide	1.49
1420361_at	Slc11a1	solute carrier family 11 (proton-coupled divalent metal ion transporters), member 1	1.45
1422788_at	Slc43a3	solute carrier family 43, member 3	1.33
1448139_at	Mlc1	megalencephalic leukoencephalopathy with subcortical cysts 1 homolog (human)	1.33
1418204_s_at	Aif1	allograft inflammatory factor 1	1.53
1434891_at	Ptgfrn	prostaglandin F2 receptor negative regulator	1.34
1448162_at	Vcam1	vascular cell adhesion molecule 1	1.50
1420911_a_at	Mfge8	milk fat globule-EGF factor 8 protein	1.33
1456685_at	Nsg2	neuron specific gene family member 2	1.59
1460218_at	Cd52	CD52 antigen	4.23
1460604_at	Cybrd1	cytochrome b reductase 1	1.32
1452533_at	Ryr3	ryanodine receptor 3	-1.54
1427746_x_at	H2-K1	histocompatibility 2, K1, K region	1.62
1449164_at	Cd68	CD68 antigen	2.75
1450678_at	Itgb2	integrin beta 2	2.02
1452961_at	Tril	TLR4 interactor with leucine-rich repeats	1.32
1418340_at	Fcer1g	Fc receptor, IgE, high affinity I, gamma polypeptide	2.00
1438654_x_at	Mmd2	monocyte to macrophage differentiation-associated 2	1.38
1424534_at	Mmd2	monocyte to macrophage differentiation-associated 2	1.34
1420895_at	Tgfbr1	transforming growth factor, beta receptor I	1.56
1437312_at	Bmpr1b	bone morphogenetic protein receptor, type 1B	1.36

1415904_at	Lpl	lipoprotein lipase	3.90
1431056_a_at	Lpl	lipoprotein lipase	1.95
1426025_s_at	Laptm5	lysosomal-associated protein transmembrane 5	1.52
1427076_at	Mpeg1	macrophage expressed gene 1	3.80
1419872_at	Csf1r	colony stimulating factor 1 receptor	1.87
1434376_at	Cd44	CD44 antigen	3.17
1423760_at	Cd44	CD44 antigen	2.76
1416066_at	Cd9	CD9 antigen	1.50
1447745_at	Aqp4	aquaporin 4	1.47
1437390_x_at	Stx1a	syntaxin 1A (brain)	1.41
1448748_at	Plek	pleckstrin	1.62
1427301_at	Cd48	CD48 antigen	1.30
**External side of plasma membrane GO:9897 (N=9; P=7.31e-06)**			
1448162_at	Vcam1	vascular cell adhesion molecule 1	1.50
1434376_at	Cd44	CD44 antigen	3.17
1423760_at	Cd44	CD44 antigen	2.76
1416066_at	Cd9	CD9 antigen	1.50
1420911_a_at	Mfge8	milk fat globule-EGF factor 8 protein	1.33
1418340_at	Fcer1g	Fc receptor, IgE, high affinity I, gamma polypeptide	2.00
1427746_x_at	H2-K1	histocompatibility 2, K1, K region	1.62
1452428_a_at	B2m	beta-2 microglobulin	2.17
1449289_a_at	B2m	beta-2 microglobulin	2.32
1447745_at	Aqp4	aquaporin 4	1.47
1427301_at	Cd48	CD48 antigen	1.30
**Extracellular region part GO:44421 (N=16; P=7.92e-06)**			
1427256_at	Vcan	versican	1.33
1448118_a_at	Ctsd	cathepsin D	1.34

1456567_x_at	Grn	granulin	1.41
1448148_at	Grn	granulin	1.33
1438629_x_at	Grn	granulin	1.38
1417266_at	Ccl6	chemokine (C-C motif) ligand 6	2.15
1417268_at	Cd14	CD14 antigen	2.66
1452428_a_at	B2m	beta-2 microglobulin	2.17
1449289_a_at	B2m	beta-2 microglobulin	2.32
1449401_at	C1qc	complement component 1, q subcomponent, C chain	3.38
1418365_at	Ctsh	cathepsin H	1.74
1443814_x_at	Ctsh	cathepsin H	1.64
1417381_at	C1qa	complement component 1, q subcomponent, alpha polypeptide	3.56
1426808_at	Lgals3	lectin, galactose binding, soluble 3	2.24
1417063_at	C1qb	complement component 1, q subcomponent, beta polypeptide	4.18
1437726_x_at	C1qb	complement component 1, q subcomponent, beta polypeptide	4.26
1415904_at	Lpl	lipoprotein lipase	3.90
1431056_a_at	Lpl	lipoprotein lipase	1.95
1418021_at	C4b	complement component 4B (Chido blood group)	2.23
1448162_at	Vcam1	vascular cell adhesion molecule 1	1.50
1420911_a_at	Mfge8	milk fat globule-EGF factor 8 protein	1.33
1417851_at	Cxcl13	chemokine (C-X-C motif) ligand 13	51.92
1448859_at	Cxcl13	chemokine (C-X-C motif) ligand 13	5.09
**Plasma membrane part GO:44459 (N=18; P=1.45e-05)**			
1438658_a_at	S1pr3	sphingosine-1-phosphate receptor 3	1.72
1460604_at	Cybrd1	cytochrome b reductase 1	1.32
1427746_x_at	H2-K1	histocompatibility 2, K1, K region	1.62
1452428_a_at	B2m	beta-2 microglobulin	2.17
1449289_a_at	B2m	beta-2 microglobulin	2.32

1450678_at	Itgb2	integrin beta 2	2.02
1436779_at	Cybb	cytochrome b-245, beta polypeptide	1.49
1418340_at	Fcer1g	Fc receptor, IgE, high affinity I, gamma polypeptide	2.00
1420895_at	Tgfbr1	transforming growth factor, beta receptor I	1.56
1448139_at	Mlc1	megalencephalic leukoencephalopathy with subcortical cysts 1 homolog (human)	1.33
1418204_s_at	Aif1	allograft inflammatory factor 1	1.53
1426025_s_at	Laptm5	lysosomal-associated protein transmembrane 5	1.52
1448162_at	Vcam1	vascular cell adhesion molecule 1	1.50
1434376_at	Cd44	CD44 antigen	3.17
1423760_at	Cd44	CD44 antigen	2.76
1416066_at	Cd9	CD9 antigen	1.50
1420911_a_at	Mfge8	milk fat globule-EGF factor 8 protein	1.33
1447745_at	Aqp4	aquaporin 4	1.47
1448748_at	Plek	pleckstrin	1.62
1427301_at	Cd48	CD48 antigen	1.30
**Intrinsic to membrane GO:31224 (N=34; P=1.90e-05)**			
1419483_at	C3ar1	complement component 3a receptor 1	1.84
1442082_at	C3ar1	complement component 3a receptor 1	1.55
1438658_a_at	S1pr3	sphingosine-1-phosphate receptor 3	1.72
1417268_at	Cd14	CD14 antigen	2.66
1437347_at	Ednrb	endothelin receptor type B	1.36
1433678_at	Pld4	phospholipase D family, member 4	1.34
1450792_at	Tyrobp	TYRO protein tyrosine kinase binding protein	4.61
1426278_at	Ifi27l2a	interferon, alpha-inducible protein 27 like 2A	1.60
1436779_at	Cybb	cytochrome b-245, beta polypeptide	1.49
1420361_at	Slc11a1	solute carrier family 11 (proton-coupled divalent metal ion transporters), member 1	1.45
1422788_at	Slc43a3	solute carrier family 43, member 3	1.33

1448139_at	Mlc1	megalencephalic leukoencephalopathy with subcortical cysts 1 homolog (human)	1.33
1434891_at	Ptgfrn	prostaglandin F2 receptor negative regulator	1.34
1448162_at	Vcam1	vascular cell adhesion molecule 1	1.50
1456685_at	Nsg2	neuron specific gene family member 2	1.59
1460218_at	Cd52	CD52 antigen	4.23
1460604_at	Cybrd1	cytochrome b reductase 1	1.32
1452533_at	Ryr3	ryanodine receptor 3	-1.54
1427746_x_at	H2-K1	histocompatibility 2, K1, K region	1.62
1449164_at	Cd68	CD68 antigen	2.75
1450678_at	Itgb2	integrin beta 2	2.02
1452961_at	Tril	TLR4 interactor with leucine-rich repeats	1.32
1418340_at	Fcer1g	Fc receptor, IgE, high affinity I, gamma polypeptide	2.00
1438654_x_at	Mmd2	monocyte to macrophage differentiation-associated 2	1.38
1424534_at	Mmd2	monocyte to macrophage differentiation-associated 2	1.34
1420895_at	Tgfbr1	transforming growth factor, beta receptor I	1.56
1437312_at	Bmpr1b	bone morphogenetic protein receptor, type 1B	1.36
1415904_at	Lpl	lipoprotein lipase	3.90
1431056_a_at	Lpl	lipoprotein lipase	1.95
1426025_s_at	Laptm5	lysosomal-associated protein transmembrane 5	1.52
1427076_at	Mpeg1	macrophage expressed gene 1	3.80
1419872_at	Csf1r	colony stimulating factor 1 receptor	1.87
1434376_at	Cd44	CD44 antigen	3.17
1423760_at	Cd44	CD44 antigen	2.76
1416066_at	Cd9	CD9 antigen	1.50
1447745_at	Aqp4	aquaporin 4	1.47
1437390_x_at	Stx1a	syntaxin 1A (brain)	1.41
1427301_at	Cd48	CD48 antigen	1.30

**Extracellular space GO:5615 (N=13; P=2.46e-05)**			
1427256_at	Vcan	versican	1.33
1448118_a_at	Ctsd	cathepsin D	1.34
1456567_x_at	Grn	granulin	1.41
1448148_at	Grn	granulin	1.33
1438629_x_at	Grn	granulin	1.38
1415904_at	Lpl	lipoprotein lipase	3.90
1431056_a_at	Lpl	lipoprotein lipase	1.95
1417063_at	C1qb	complement component 1, q subcomponent, beta polypeptide	4.18
1437726_x_at	C1qb	complement component 1, q subcomponent, beta polypeptide	4.26
1417266_at	Ccl6	chemokine (C-C motif) ligand 6	2.15
1417268_at	Cd14	CD14 antigen	2.66
1418021_at	C4b	complement component 4B (Chido blood group)	2.23
1452428_a_at	B2m	beta-2 microglobulin	2.17
1449289_a_at	B2m	beta-2 microglobulin	2.32
1448162_at	Vcam1	vascular cell adhesion molecule 1	1.50
1420911_a_at	Mfge8	milk fat globule-EGF factor 8 protein	1.33
1418365_at	Ctsh	cathepsin H	1.74
1443814_x_at	Ctsh	cathepsin H	1.64
1417851_at	Cxcl13	chemokine (C-X-C motif) ligand 13	51.92
1448859_at	Cxcl13	chemokine (C-X-C motif) ligand 13	5.09
**Membrane GO:16020 (N=43; P=2.53e-05)**			
1419483_at	C3ar1	complement component 3a receptor 1	1.84
1442082_at	C3ar1	complement component 3a receptor 1	1.55
1438658_a_at	S1pr3	sphingosine-1-phosphate receptor 3	1.72
1417268_at	Cd14	CD14 antigen	2.66
1437347_at	Ednrb	endothelin receptor type B	1.36

1433678_at	Pld4	phospholipase D family, member 4	1.34
1450792_at	Tyrobp	TYRO protein tyrosine kinase binding protein	4.61
1452428_a_at	B2m	beta-2 microglobulin	2.17
1449289_a_at	B2m	beta-2 microglobulin	2.32
1426278_at	Ifi27l2a	interferon, alpha-inducible protein 27 like 2A	1.60
1437874_s_at	Hexb	hexosaminidase B	2.26
1436779_at	Cybb	cytochrome b-245, beta polypeptide	1.49
1420361_at	Slc11a1	solute carrier family 11 (proton-coupled divalent metal ion transporters), member 1	1.45
1422788_at	Slc43a3	solute carrier family 43, member 3	1.33
1426808_at	Lgals3	lectin, galactose binding, soluble 3	2.24
1448139_at	Mlc1	megalencephalic leukoencephalopathy with subcortical cysts 1 homolog (human)	1.33
1418204_s_at	Aif1	allograft inflammatory factor 1	1.53
1434891_at	Ptgfrn	prostaglandin F2 receptor negative regulator	1.34
1448162_at	Vcam1	vascular cell adhesion molecule 1	1.50
1436314_at	Scyl2	SCY1-like 2 (*S. cerevisiae*)	2.33
1420911_a_at	Mfge8	milk fat globule-EGF factor 8 protein	1.33
1456685_at	Nsg2	neuron specific gene family member 2	1.59
1460218_at	Cd52	CD52 antigen	4.23
1460604_at	Cybrd1	cytochrome b reductase 1	1.32
1452533_at	Ryr3	ryanodine receptor 3	-1.54
1427746_x_at	H2-K1	histocompatibility 2, K1, K region	1.62
1449164_at	Cd68	CD68 antigen	2.75
1450678_at	Itgb2	integrin beta 2	2.02
1452961_at	Tril	TLR4 interactor with leucine-rich repeats	1.32
1418340_at	Fcer1g	Fc receptor, IgE, high affinity I, gamma polypeptide	2.00
1449514_at	Grk5	G protein-coupled receptor kinase 5	1.37
1438654_x_at	Mmd2	monocyte to macrophage differentiation-associated 2	1.38

1424534_at	Mmd2	monocyte to macrophage differentiation-associated 2	1.34
1420895_at	Tgfbr1	transforming growth factor, beta receptor I	1.56
1437312_at	Bmpr1b	bone morphogenetic protein receptor, type 1B	1.36
1415904_at	Lpl	lipoprotein lipase	3.90
1431056_a_at	Lpl	lipoprotein lipase	1.95
1426025_s_at	Laptm5	lysosomal-associated protein transmembrane 5	1.52
1427076_at	Mpeg1	macrophage expressed gene 1	3.80
1426509_s_at	Gfap	glial fibrillary acidic protein	5.15
1440142_s_at	Gfap	glial fibrillary acidic protein	3.35
1426508_at	Gfap	glial fibrillary acidic protein	4.45
1419872_at	Csf1r	colony stimulating factor 1 receptor	1.87
1434376_at	Cd44	CD44 antigen	3.17
1423760_at	Cd44	CD44 antigen	2.76
1416066_at	Cd9	CD9 antigen	1.50
1447745_at	Aqp4	aquaporin 4	1.47
1437390_x_at	Stx1a	syntaxin 1A (brain)	1.41
1448748_at	Plek	pleckstrin	1.62
1427301_at	Cd48	CD48 antigen	1.30
**Plasma membrane GO:5886 (N=28; P=3.31e-05)**			
1419483_at	C3ar1	complement component 3a receptor 1	1.84
1442082_at	C3ar1	complement component 3a receptor 1	1.55
1438658_a_at	S1pr3	sphingosine-1-phosphate receptor 3	1.72
1460604_at	Cybrd1	cytochrome b reductase 1	1.32
1417268_at	Cd14	CD14 antigen	2.66
1427746_x_at	H2-K1	histocompatibility 2, K1, K region	1.62
1437347_at	Ednrb	endothelin receptor type B	1.36
1452428_a_at	B2m	beta-2 microglobulin	2.17

1449289_a_at	B2m	beta-2 microglobulin	2.32
1449164_at	Cd68	CD68 antigen	2.75
1450678_at	Itgb2	integrin beta 2	2.02
1449514_at	Grk5	G protein-coupled receptor kinase 5	1.37
1418340_at	Fcer1g	Fc receptor, IgE, high affinity I, gamma polypeptide	2.00
1436779_at	Cybb	cytochrome b-245, beta polypeptide	1.49
1420361_at	Slc11a1	solute carrier family 11 (proton-coupled divalent metal ion transporters), member 1	1.45
1420895_at	Tgfbr1	transforming growth factor, beta receptor I	1.56
1415904_at	Lpl	lipoprotein lipase	3.90
1431056_a_at	Lpl	lipoprotein lipase	1.95
1448139_at	Mlc1	megalencephalic leukoencephalopathy with subcortical cysts 1 homolog (human)	1.33
1418204_s_at	Aif1	allograft inflammatory factor 1	1.53
1426025_s_at	Laptm5	lysosomal-associated protein transmembrane 5	1.52
1419872_at	Csf1r	colony stimulating factor 1 receptor	1.87
1448162_at	Vcam1	vascular cell adhesion molecule 1	1.50
1434376_at	Cd44	CD44 antigen	3.17
1423760_at	Cd44	CD44 antigen	2.76
1416066_at	Cd9	CD9 antigen	1.50
1420911_a_at	Mfge8	milk fat globule-EGF factor 8 protein	1.33
1447745_at	Aqp4	aquaporin 4	1.47
1437390_x_at	Stx1a	syntaxin 1A (brain)	1.41
1460218_at	Cd52	CD52 antigen	4.23
1427301_at	Cd48	CD48 antigen	1.30
1448748_at	Plek	pleckstrin	1.62
**Cell periphery GO:71944 (N=28; P=5.23e-05)**			
1419483_at	C3ar1	complement component 3a receptor 1	1.84
1442082_at	C3ar1	complement component 3a receptor 1	1.55

1438658_a_at	S1pr3	sphingosine-1-phosphate receptor 3	1.72
1460604_at	Cybrd1	cytochrome b reductase 1	1.32
1417268_at	Cd14	CD14 antigen	2.66
1427746_x_at	H2-K1	histocompatibility 2, K1, K region	1.62
1437347_at	Ednrb	endothelin receptor type B	1.36
1452428_a_at	B2m	beta-2 microglobulin	2.17
1449289_a_at	B2m	beta-2 microglobulin	2.32
1449164_at	Cd68	CD68 antigen	2.75
1450678_at	Itgb2	integrin beta 2	2.02
1449514_at	Grk5	G protein-coupled receptor kinase 5	1.37
1418340_at	Fcer1g	Fc receptor, IgE, high affinity I, gamma polypeptide	2.00
1436779_at	Cybb	cytochrome b-245, beta polypeptide	1.49
1420361_at	Slc11a1	solute carrier family 11 (proton-coupled divalent metal ion transporters), member 1	1.45
1420895_at	Tgfbr1	transforming growth factor, beta receptor I	1.56
1415904_at	Lpl	lipoprotein lipase	3.90
1431056_a_at	Lpl	lipoprotein lipase	1.95
1448139_at	Mlc1	megalencephalic leukoencephalopathy with subcortical cysts 1 homolog (human)	1.33
1418204_s_at	Aif1	allograft inflammatory factor 1	1.53
1426025_s_at	Laptm5	lysosomal-associated protein transmembrane 5	1.52
1419872_at	Csf1r	colony stimulating factor 1 receptor	1.87
1448162_at	Vcam1	vascular cell adhesion molecule 1	1.50
1434376_at	Cd44	CD44 antigen	3.17
1423760_at	Cd44	CD44 antigen	2.76
1416066_at	Cd9	CD9 antigen	1.50
1420911_a_at	Mfge8	milk fat globule-EGF factor 8 protein	1.33
1447745_at	Aqp4	aquaporin 4	1.47
1437390_x_at	Stx1a	syntaxin 1A (brain)	1.41

1460218_at	Cd52	CD52 antigen	4.23
1427301_at	Cd48	CD48 antigen	1.30
1448748_at	Plek	pleckstrin	1.62
